# Single nucleotide polymorphism (SNP) analysis reveals ancestry and genetic diversity of cultivated and wild grapevines in Croatia

**DOI:** 10.1186/s12870-024-05675-4

**Published:** 2024-10-17

**Authors:** Luka Marinov, Gabriele Magris, Gabriele Di Gaspero, Michele Morgante, Edi Maletić, Marijan Bubola, Ivan Pejić, Goran Zdunić

**Affiliations:** 1 Institute for Adriatic Crops and Karst Reclamation, Split, Croatia; 2 Centre of Excellence for Biodiversity and Molecular Plant Breeding, Zagreb, Croatia; 3 Istituto di Genomica Applicata, Udine, Italy; 4 Department of Agricultural, Food, Environmental and Animal Sciences, University of Udine, Udine, Italy; 5 Fondazione per la Ricerca Genomica ed Epigenomica, Udine, Italy; 6 University of Zagreb, Faculty of Agriculture, Zagreb, Croatia; 7 Institute of Agriculture and Tourism, Poreč, Croatia

**Keywords:** SNP, *V. vinifera*, Genealogical relationships, GBS, GWAS

## Abstract

**Background:**

Croatia is a geographically small country with a remarkable diversity of cultivated and spontaneous grapevines. Local germplasm has been characterised by microsatellite markers, but a detailed analysis based on single nucleotide polymorphisms (SNPs) is still lacking. Here we characterize the genetic diversity of 149 accessions from three germplasm repositories and four natural sites using 516,101 SNPs to identify complete parent-offspring trios and their relations with spontaneous populations, offering a proof-of-concept for the use of reduced-representation genome sequencing in population genetics and genome-wide association studies (GWAS).

**Results:**

Principal component analysis revealed a clear discontinuity between cultivated (*V. vinifera* subsp. *sativa*) and spontaneous grapevines, supporting the notion that the latter represent local populations of the wild progenitor (*V. vinifera* subsp. *sylvestris*). ADMIXTURE identified three ancestry components. Two *sativa* components are alternatively predominant in cultivars grown either in northern Adriatic Croatia and Continental Croatia or in Dalmatia (i.e. central and southern Adriatic Croatia). A *sylvestris* component, which is predominant in accessions from spontaneous populations, is a minor ancestry component in cultivated accessions. TREEMIX provided evidence of unidirectional migration from the vineyards to natural sites, suggesting that gene flow has gone preferentially from the introduced domesticated germplasm into local wild populations rather than vice versa. Identity-by-descent analysis indicated an extensive kinship network, including 14 complete parent-offspring trios, involving only cultivated accessions, six full-sibling relationships and invalidated a presumed pedigree of one of the most important varieties in Croatia, ‘Plavac Mali’. Despite this strong population structure, significant association was found between 143 SNPs and berry skin colour and between 2 SNPs and leaf hairiness, across two previously known genomic regions.

**Conclusions:**

The clear genetic separation between Croatian cultivars and *sylvestris* ruled out the hypothesis that those cultivars originated from local domestication events. On the other hand, the evidence of a crop-to-wild gene flow signals the need for an urgent adoption of conservation strategies that preserve the residual genetic integrity of wild relatives. The use of this reduced-representation genome sequencing protocol in grapevine enables an accurate pedigree reconstruction and can be recommended for GWAS experiments.

**Supplementary Information:**

The online version contains supplementary material available at 10.1186/s12870-024-05675-4.

## Background

Grapevine (*Vitis vinifera* subsp. *sativa*) is an economically important fruit crop in the Mediterranean Basin and the predominantly cultivated species worldwide. The wild form *V. vinifera* subsp. *sylvestris* naturally occurs only in Europe and Western Asia. The process of domestication, which took place approximately 11,000 years ago [1] and was followed by selection, breeding, migration and wild introgression, has resulted in the cultivated germplasm being more diverse and more heterozygous than the present-day populations of its wild progenitor [2, 3]. Despite being irrelevant for human consumption, the subspecies *sylvestris* represents an important source of resistance genes against pathogens [4–6], resilience traits against abiotic stresses [7, 8] and beneficial endophytic microbial communities [9].

The exact events that led to grapevine domestication are still controversial [1, 10, 11], in particular whether domestication genes were selected once or twice. What appears to be undisputable is the evidence that domesticated grapevines underwent introgression events from *sylvestris*, as they were moved from the cradle of domestication along migration and trading routes. Introgression from *sylvestris* populations in Western Europe has left a discernible footprint on the genomes of Western wine grapes [1, 10, 12, 13]. This may have helped grapevines to diversify and adapt to new environments.

The diversity in cultivated grapes and the variation in natural populations originate from a combination of evolutionary forces, anthropic activities and historical circumstances [1, 3, 14, 15], at global and local geographical scales. Yield, flavour, taste and berry colour were the major targets of artificial selection. Using a large set of 2,096 genotypes, early genetic analyses defined three distinct groups, corresponding to three main ecogeographical *proles*, resulting from selection for either wine making or fresh consumption and from adaptation to different climates in the Mediterranean Sea basin, mainland Europe and Central Asia [14]. The genotyping of national germplasm collections and locally grown cultivars [16–24] and the sequencing of archaeological pips [25] have clarified that a few ancient cultivars, either due to their value of cultivation or simply as a result of founder effects, have generated many descendants within a few generations of sexual reproduction, before the resulting heterozygous genotypes were fixed by vegetative propagation. DNA paternity testing has revealed kinships that would never be predicted upon resemblance and descent groups of highly related cultivars that now dominate the market of varietal wines. In the wild compartment, studies have warned that there is genetic erosion and inbreeding, probably due to anthropogenic environmental impact, small effective size of natural populations and geographical isolation [2, 3].

Within this global scenario, it is important to preserve and characterize local cultivars and wild populations, even in a cultivated species where breeding through crossing and selection has played a minor role in the last few centuries. At least 127 cultivars are reported in Croatia [16, 17]. Many of them are cultivated over limited areas. Some of them are only conserved in germplasm repositories. Several spontaneous populations have been documented in natural sites [26]. So far, genetic analysis suggested loose links between European cultivars and Croatian *sylvestris* [27, 28]. Phenotypic diversity is quite large countrywide. Wine grape cultivars are predominant in terms of numbers and cultivated land, while only a few table grape cultivars are grown for fresh consumption. A few complete parent-offspring trios and several parent-offspring relationships were identified using SSR markers [16]. Nonetheless, Mendelian inconsistencies were frequent in genuine pedigrees due to the hidden presence of non-amplifying PCR alleles and the somatic hypervariability of microsatellite DNA. Bacilieri and colleagues found that Croatian cultivars tend to cluster with the group of Balkan and Eastern Europe wine cultivars [14]. However, a small number of Croatian cultivars were included in their analysis and the origin of the vast majority of minor cultivars is still unknown. Several historical cultivars have a tight genetic relationship with cultivars grown in Italy on the western and northern shores of the Adriatic Sea basin, opposite to Croatian shores. Numerous synonymies of important genotypes have been discovered in the two countries such as ‘Tribidrag’ and ‘Primitivo’, ‘Maraština’ and ‘Malvasia Bianca Lunga’, and ‘Verdić’ and ‘Glera’ [29]. Common cultivars such as ‘Bombino Bianco’, ‘Heunisch Weiss’ (synonym of ‘Belina Starohrvatska’) and ‘Blank Blauer’ (synonym of ‘Bljuzgavac’ and’ ‘Vulpea’) were shown to be important progenitors in both countries [16, 24, 29, 30].

We have recently used a targeted sequencing method known as Single Primer Enrichment Technology (SPET), which was designed to sample 50,000 genic regions, to generate a half million SNPs from Croatian grapevine germplasm [29]. Although whole-genome resequencing (WGS) is rapidly becoming available at an affordable cost [1], we used this SNP dataset to show that reduced-representation genome sequencing is a cost-effective alternative for pedigree reconstruction, population genetic analysis and genome-wide association studies (GWAS). We reveal with the Croatian case-study that national and transnational grapevine diversity is hierarchically interconnected in Europe, with significant implications for germplasm exploitation in viticulture and for GWAS design.

## Materials and methods

### Plant material

Genotypic data of 149 grapevine accessions, representing unique genotypes, were obtained from previous work [29]. Of these, 108 accessions are traditional cultivars (*Vitis vinifera* L. subsp. *sativa*) grown in Croatia. The plant material is maintained at three Croatian germplasm repositories: Institute of Adriatic Crops and Karst Reclamation, Split; Institute of Agriculture and Tourism, Poreč; University of Zagreb. The remaining 41 accessions, which putatively belonged to the wild grape subspecies (*Vitis vinifera* L. subsp. *sylvestris* Gmelin), are growing spontaneously in natural habitats at three locations in Croatia (Modro jezero, Paklenica, Psunj) and one location past the State border in Bosnia and Herzegovina (Cerovica). Modro jezero is a small lake at the bottom of a sinkhole located in Dalmatian mainland, with ample fluctuations in water depth. Its south-eastern banks, characterized by steep gravel slopes, are adjacent to the residential area of the town of Imotski. Paklenica is also located in Dalmatia, about 170 km North West of Modro jezero. Paklenica National Park is a coastal mountain area characterized by a deep canyon through which the homonymous river flows, flanked by banks with lush vegetation, where spontaneous grapevines were found. Psunj is a mountain in Continental Croatia about 190 km North East of Paklenica and represents a forest habitat. Cerovica is the southernmost location in a karst mountain area characterized by a lack of natural water reservoirs, approximately 100 Km South East of Modro jezero. Comprehensive information is provided in Table S1.

### Genetic diversity and identification of population structure

Analyses were conducted using 516,101 SNPs from the dataset of this article and 224,695 SNPs in common between the dataset of this article and a WGS dataset from Magris and colleagues [12], which included a diversity panel of *sativa* genotypes and *sylvestris* samples from the ‘Ketsch’ population in Germany (from now on WGS diversity panel). Principal component analysis (PCA) was performed using SNPrelate. Analysis of population structure was performed using the ADMIXTURE software [31]. The best K value was determined by the lowest cross-validation error for each K value (2–15) to determine the most appropriate number of ancestry components. Split and migration events between groups and *f3* test were calculated using the TREEMIX software [32]. The groups of cultivated accessions for TREEMIX analysis were generated using the output of ADMIXTURE K = 3 groups. Accessions with one ancestry component higher than 0.85 were assigned to either of the two *sativa* groups. The remaining ones were assigned to a *sativa* admixed group. The TREEMIX groups of spontaneous grapevines corresponded, the first one, to all individuals from the four sampling locations but one individual and, the other one, to the outlier individual from the Modro jezero population (Sy10). The variance of relatedness between groups explained by each model was calculated using RADpipe [GitHub RADpipe repository, 10.5281/zenodo.17809].

### Parentage analysis and kinship network

Only cultivated accessions were considered for parentage analysis. Thresholds of identity-by-state (IBS) ratio and genotypic distance were used to estimate pairwise identity-by-descent (IBD) in each genomic window of 200 Kb of non-repetitive DNA. IBS ratio and genotypic distance was calculated as described by Magris and colleagues [12]. Parentage analysis was performed based on the IBD cumulative length and segment length distribution of the IBD0, IBD1 and IBD2 windows. The following parameters were used to determine parent-offspring relationship: IBD0 < 15%, IBD1 > 60% of genome length, longest IBD0 segment length < 3.8 Mbp.

For each hypothetical trio, we considered biallelic SNPs that were informative in all 3 individuals. If P1×P2→F1 were the individuals under comparison in the reported direction of the relationship and “a” and “b” were the reference and alternative alleles, the following genotypic combinations were compatible with the hypothesis: aa×aa→aa, aa×ab→aa, aa×ab→ab, aa×bb→ab, ab×aa→aa, ab×aa→ab, ab×ab→aa, ab×ab→ab, ab×ab→bb, ab×bb→ab, ab×bb→bb, bb×aa→ab, bb×ab→ab, bb×ab→bb, bb×bb→bb. On the contrary, the following genotypic combinations were incompatible with the hypothesis: aa×aa→ab, aa×aa→bb, aa×ab→bb, aa×bb→aa, aa×bb→bb, ab×aa→bb, ab×bb→aa, bb×aa→aa, bb×aa→bb, bb×ab→aa, bb×bb→aa, bb×bb→ab. Shortly, alleles in F1 that were not shared with P1 had to be shared with P2, and vice versa. Sites that did not meet this requirement rejected the hypothesis. As hemizygous DNA is present in each parental genome and some heterozygous sites can be erroneously called homozygous due to insufficient read coverage, a background noise of unmatching sites is expected in the offspring, which we modelled in terms of number and chromosomal distribution using known genuine trios. The false rate of Mendelian inconsistencies was identified in a percentage of 0.8% of all informative sites. The parental combination for ‘Karstičevica’ showed a slightly higher false rate of 1.17% Mendelian inconsistencies likely due to fewer informative sites in both parents compared to other comparisons, but the distribution of unmatching SNPs across the genome met the requirement of being randomly distributed. Full-sibling relationships were inferred from the comparison of the resolved trios. The information on parent-offspring pairs, complete parent-offspring trios and full-siblings was summarized graphically with a kinship network. The network was generated in R using the igraph package [33, 34].

### Phenotypic data and GWAS

Phenotyping was performed in a common garden experiment using a reduced panel of 84 accessions, from which highly consanguineous individuals were removed. Two-bud cuttings of 70 cultivated and 14 spontaneous accessions were self-rooted in a perlite-based medium. The rooted cuttings were transplanted into 5-litre potted medium, consisting of a mixture of natural soil, humus, quartz sand and perlite, and grown in a greenhouse at the Institute for Adriatic Crops and Karst Reclamation, Split, Croatia, without supplemental lighting and heating. Each genotype was represented by six replicates. Vines were pruned back to two buds during winter dormancy. Visual scoring of leaf hairiness was performed during the second annual cycle of vegetative growth based on OIV descriptors [35]. The list of accessions with the corresponding OIV descriptors are provided in Table S2. The density of prostrate hairs between the main veins on the lower side of the leaf blade (OIV 084) was visually assessed using 5 categorical values. Ten mature leaves from the middle third of six shoots per genotype were examined, as described in 2nd Edition of the OIV Descriptor List for Grape Varieties and *Vitis* Species [35]. In order to have a positive control for GWAS, ensuring that sample size, population structure and kinship were neither limiting factors and nor sources of false positives, we also scored berry colour. GWAS was performed using the Efficient Mixed-Model Association eXpedited (EMMAX) algorithm [36, 37] and 306,965 SNPs with a MAF higher than 0.01. A kinship matrix was used for correcting for kinship. The P-value of individual SNPs was adjusted for multiple testing using the Benjamini-Hochberg correction. Statistically significant thresholds were determined using the Bonferroni method with α = 0.05. Q-Q plots and Manhattan plots were generated using the qqman library in R. Linkage disequilibrium was calculated using PLINK [38].

## Results

### Genetic diversity, population structure and ancestry components

**Fig. 1:**
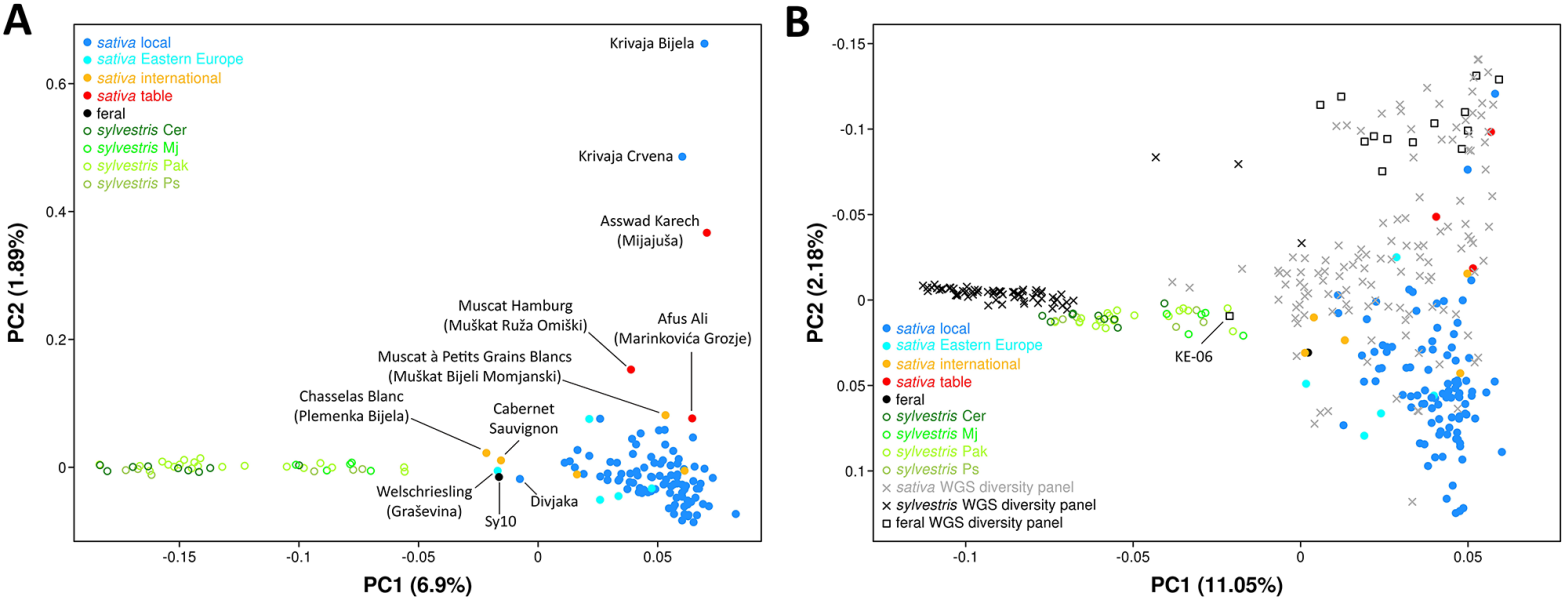
Principal component analysis (PCA) of Croatian germplasm alone (**A**) or including a WGS diversity panel (**B**). The bidimensional plots show the first two components (eigenvalues) of the PCA

The first two components of the PCA explained 8.8% of the genotypic variation in 149 accessions (Fig. 1A). The first component showed a clear separation between Croatian *sylvestris* and locally grown *sativa* along the x-axis of the bidimensional plot. One spontaneous accession (Sy10) from the Modro jezero population laid in an intermediate position along PC1 nearby widespread cultivars with high European *sylvestris* ancestry such as ‘Chasselas Blanc’ and ‘Welschriesling’ (locally grown in Croatia under the synonymous of ‘Plemenka Bijela’ and ‘Graševina’, respectively), ‘Cabernet Sauvignon’, and the Dalmatian cultivar ‘Divjaka’. It is therefore possible that Sy10 is either an abandoned local cultivar or a seedling that was involuntarily introduced into the anthropic environment of Modro jezero or a feral accession that was derived from crop-to-wild gene flow in a once wild population. The cultivated germplasm in Croatia laid in a confined part of the bidimensional space that is delimited by well-known grapevines, which are cultivated in Croatia and other European countries. The few exceptions are represented by three table grapes ‘Krivaja Bijela’, ‘Krivaja Crvena’ and ‘Mijajuša’, the latter of which is a synonym of the Levantine cultivar ‘Asswad Karech’, a prolific progenitor of many table grapes [39], and is known in Greece with the synonym ‘Xeromachairouda’ [40]. In order to rescale the diversity in Croatia relative to the global grapevine diversity, we performed a PCA using 224,695 common variant sites between the sample set of this article and a WGS diversity panel. The bidimensional plot in Fig. 1B shows that grapevine diversity in Croatia is relatively rich. Diversity and diversification in local *sylvestris* appear to be relatively small and low compared to other European wild populations. Croatian natural populations may contain a mixture of genuine *sylvestris* and *sylvestris*-*sativa* admixed individuals. A number of individuals from all but one natural site laid, indeed, midway in the bidimensional space between the *sylvestris* and *sativa* distributions and colocalized with KE-06, a known *sylvestris*-*sativa* hybrid from a German natural site.

**Fig. 2:**
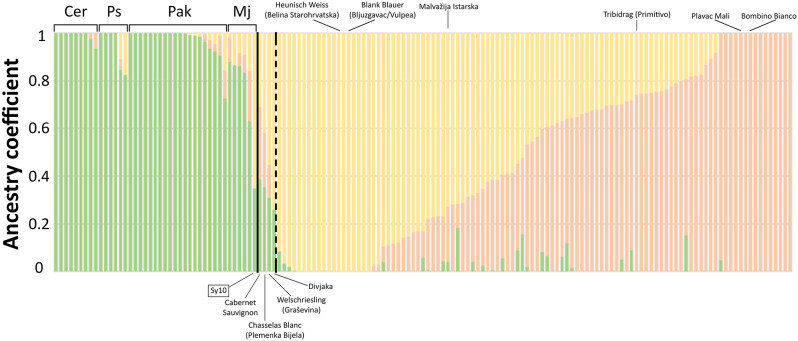
Population structure based on K = 3 ancestry components. Green bars represent *sylvestris* ancestry. Yellow bars represent Heunisch Weiss/Blank Blauer *sativa* ancestry. Orange bars represent Plavac Mali/Bombino Bianco *sativa* ancestry. Cer, Ps, Pak and Mj indicate natural sites. Spontaneous accessions are ordered by decreasing *sylvestris* ancestry coefficients within each natural site. Cultivars with > 0.2 *sylvestris* ancestry are ordered between the vertical solid and dashed lines. The remaining cultivars (right to the dashed line) are ordered by increasing Plavac Mali/Bombino Bianco *sativa* ancestry

To substantiate the findings inferred from the PCA, we analysed population structure using a model-based clustering approach that simultaneously estimates population allele frequencies along with ancestry proportions, implemented in the ADMIXTURE software [31]. The cross-validation error indicated that three ancestry components (K = 3) contributed most likely to the genome composition of the analysed germplasm (Table S3). According to ADMIXTURE, one ancestry component, which we refer hereafter to as *sylvestris*, is predominant in natural sites and is present as a minor contributor (< 0.2) in a few local cultivars (Fig. 2). ADMIXTURE confirmed that ‘Cabernet Sauvignon’, ‘Chasselas Blanc’, ‘Welschriesling’ (widely grown in Continental Croatia), and ‘Divjaka’, which is grown exclusively in the Pelješac peninsula, contain a relevant fraction of this ancestry component. Natural sites are home to individuals with pure *sylvestris* ancestry but also to individuals with varying proportions of *sylvestris*-*sativa* admixed ancestry. In all but one of the cases, *sylvestris* ancestry was largely predominant. The *sylvestris* ancestry component contributed for a fraction from 0.63 to 1 in each individual genome, with a mean of 0.95 and a median of 1 considering the whole sample set. The most relevant exception is represented by the individual Sy10 from Modro jezero that showed a predominant 0.65 *sativa* ancestry component and is part of the peculiarity of the Modro jezero spontaneous population, wherein all individuals show some degree of admixture. The two ancestry components that, cumulatively, are predominant in the individual genomes of all cultivars, which we refer hereafter to as *sativa*, contribute almost equally to the global composition of the cultivated germplasm analysed in this article (Fig. 2). One *sativa* ancestry component is associated with cultivars typically grown in Continental Croatia and northern Adriatic Croatia (Istrian peninsula and coastal/insular Kvarner, Fig. S1) and accounts for the entirety of ancestry in the cultivars ‘Heunisch Weiss’ and ‘Blank Blauer’. The other *sativa* ancestry component is associated with cultivars typically grown in Dalmatia (i.e. central and southern Adriatic Croatia, Fig. S1) and accounts for the entire ancestry in the cultivars ‘Plavac Mali’ and ‘Bombino Bianco’. The majority of Croatian cultivars showed admixed proportions of these two *sativa* ancestry components. Among the most remarkable ones, ‘Malvazija Istarska’ showed approximately three quarters of Continental/Istrian *sativa* ancestry and one quarter of Dalmatian *sativa* ancestry. ‘Tribidrag’ showed the opposite proportion. ‘Tribidrag’ is widely grown on the Eastern shores of the Adriatic Sea under the synonyms of ‘Pribidrag’ and ‘Crljenak Kaštelanski’ in Croatia and ‘Kratošija in Montenegro. The same genotype is associated with the prime name ‘Primitivo’ in the South-Eastern part of the Italian peninsula on Adriatic and Ionian shores and it has been introduced into Californian viticulture with the synonym of ‘Zinfandel’.

### Origin of admixed ancestries and genealogical relationships

PCA and ADMIXTURE suggested that spontaneous populations in natural sites are in part composed of admixed individuals. The results of ADMIXTURE also conveyed a sense of strong kinship influencing population structure in the cultivated germplasm. To address these points, we investigated the introgression events that have generated admixed individuals, using the algorithm developed by Pickrell and Pritchard and implemented in the TREEMIX software [32]. We also searched for complete parent-offspring trios in all possible combinations and permutations of the analysed accessions.

**Fig. 3:**
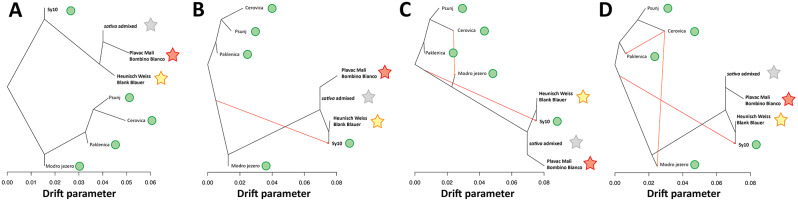
Split and migration events between groups. Green dots highlight spontaneous populations and accessions. Stars highlight cultivated groups. Yellow stars represent accessions grouped by ≥ 0.85 Heunisch Weiss/Blank Blauer ancestry. Orange stars represent accessions grouped by ≥ 0.85 Plavac Mali/Bombino Bianco ancestry. Arrows indicate migrations. Panels report: (**A**) no migration event, (**B**) one migration event, (**C**) two migration events and (**D**) three migration events

Figure 3 shows the TREEMIX models using a block size of 200 SNPs that explained from 98.3 to 99.6% of the variance of relatedness among groups, with increasing numbers of migration events. Tree topologies confirm that the spontaneous populations in more remote forest environments in Continental Croatia and in Bosnia Herzegovina are the least related ones to local cultivars. The combination of evidence from ADMIXTURE and TREEMIX suggests that the spontaneous populations in Dalmatia from the sites that were most exposed to the effects of anthropization on the banks of Modro jezero and in the Paklenica National Park show stronger signatures of *sativa* introgression. The *f3* test confirmed that the spontaneous populations of Modro jezero and Paklenica National Park are compatible with being the result of admixture between more genuine *sylvestris* populations, similar to those found at Cerovica and Psunj, and cultivated germplasm (Table S4). To better estimate the extent of *sativa* introgression in individuals of spontaneous populations, we searched for the presence in putatively *sylvestris* individuals of the most common white grape *sativa* haplotype that impairs anthocyanin biosynthesis in berries as a consequence of two nearby recessive causal mutations in a *MybA* array, hereafter referred to as white haplotype. ‘Bombino Bianco’ is homozygous for this haplotype all the way through the lower arm of chromosome 2, wherein the *MybA* array is located (Fig. S2-S9). We generated plots of haplotype sharing between all accessions and ‘Bombino Bianco’ across 50 genomic windows along chromosome 2 (Fig. S10-S12). The white haplotype around the *MybA* array showed a frequency of 0.78 in the cultivated germplasm of the present article (Fig. S10-S11 and Table S5). We found this *sativa* haplotype in all four surveyed spontaneous populations in Croatia (Fig. S12 Table S5). The white haplotype is, indeed, present with a frequency of 0.06 in the individuals of the population of Cerovica, 0.17 in the populations of Modro jezero and Psunj, and 0.40 in the population of Paklenica. The individuals of the spontaneous populations that share this haplotype with *sativa* share a segment of some million nucleotides that extends from upstream the *MybA* array down, in several cases, to the lower end of the chromosome. This condition can only be explained by a relatively recent gene flow going from the vineyards to the wild populations and not in the opposite direction.

**Fig. 4:**
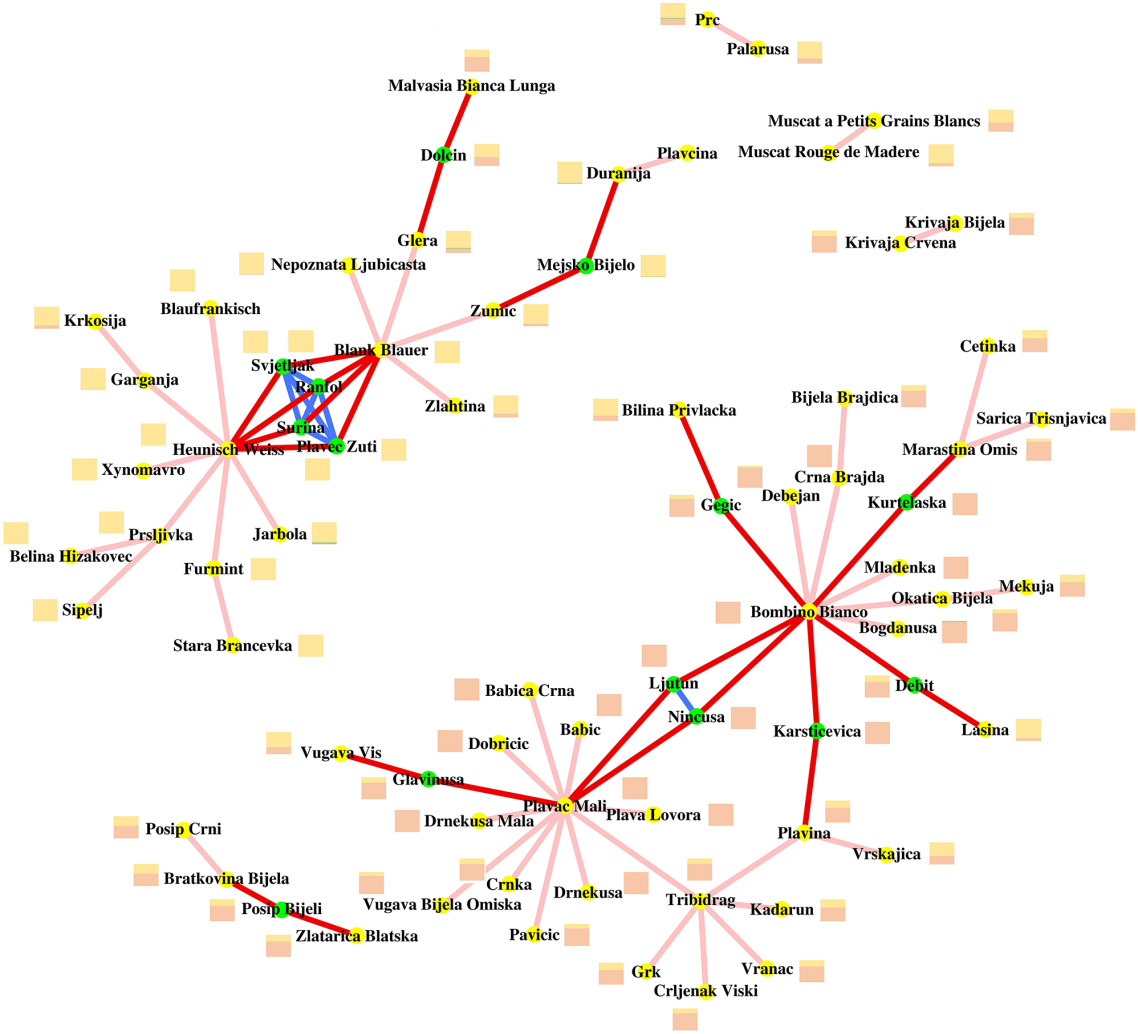
Network map of kinship relationships. Red lines indicate oriented parent-offspring relationships in the direction of the cultivar identified with the green dot. Pink lines indicate unoriented parent-offspring relationships (unknown direction). Blue lines connect full-siblings. The histogram next to each cultivar indicates the ancestry proportions shown in Fig. 2

Fourteen complete parent-offspring trios were identified in the dataset of this article (Fig. S13-S26). One of them is new and the others confirm previous reports (Table 1). ‘Heunisch Weiss’, ‘Blank Blauer’, ‘Plavac Mali’ and ‘Bombino Bianco’ were recurrent parents in 11 out of 14 trios. The pair ‘Heunisch Weiss’ and ‘Blank Blauer’ has generated four cultivars. One of these full-siblings is the cultivar ‘Surina’, which is reported here for the first time along with ‘Svjetljak’, ‘Ranfol’, and ‘Plavec žuti’. The comparison based on IBD segment length of four full-siblings is graphically shown in Fig. S27-S32. The pair ‘Plavac Mali’ and ‘Bombino Bianco’ generated two cultivars, i.e. the full-siblings ‘Ninčuša’ and ‘Ljutun’ shown in Fig. S33. A total of 72 parent-offspring pairwise relationships were identified in the dataset of this article (Table S6). All these relationships are summarised graphically in the kinship network of Fig. 4.

**Table 1:** List of complete parent-offspring trios

Offspring	Parent 1	Parent 2	Mendelian errors	Informative sites	Previous reference
Ljutun	Bombino Bianco	Plavac Mali	0.66%	387,242	[16]
Ninčuša	Bombino Bianco	Plavac Mali	0.67%	378,126	[21]
Gegić	Bombino Bianco	Bilina Privlačka	0.66%	430,842	[16]
Karstičevica	Bombino Bianco	Plavina	1.17%	266,891	[16]
Debit	Bombino Bianco	Lasina	0.64%	427,447	[21]
Kurtelaška	Bombino Bianco	Maraština Omiš	0.53%	411,318	[21]
Ranfol	Heunisch Weiss	Blank Blauer	0.70%	285,861	[21]
Surina	Heunisch Weiss	Blank Blauer	0.66%	287,812	this paper
Plavec žuti	Heunisch Weiss	Blank Blauer	0.71%	287,823	[16]
Svjetljak	Heunisch Weiss	Blank Blauer	0.71%	287,881	[16]
Mejsko Bijelo	Duranija	Žumić	0.73%	225,938	[16]
Glavinuša	Vugava Vis	Plavac Mali	0.55%	397,147	[21]
Dolcin	Malvasia Bianca Lunga	Glera	0.51%	481,631	[48, 49]
Pošip Bijeli	Zlatarica Blatska	Bratkovina Blatska	0.66%	428,187	[50]

As a result of the presence of complete trios and many other pairwise unoriented parent-offspring relationships, 23 cultivars are linked to ‘Heunisch Weiss’ and ‘Blank Blauer’ through a chain of parent-offspring relationships and 36 cultivars are linked to ‘Plavac Mali’ and ‘Bombino Bianco’. The two kinship groups, one named after its founders and referred to as Heunisch Weiss/Blank Blauer and the other one referred to as Plavac Mali/Bombino Bianco, represent collectively 56% of the analyzed cultivars. Consanguinity is furtherly exacerbated within each group and between groups by two other factors. Within each group, progenies deriving from minor progenitors tend to originate from genotypes that share the same major ancestry component with the major progenitors in the same group (Fig. 4), with very rare exceptions represented by ‘Debit’, ‘Dolcin’, ‘Gegić’ and ‘Glavinuša’. This seems to suggest that kinship and regional ancestry distribution acted in the same direction in generating homogeneous groups of local cultivars. Between groups, the four major founders are themselves consanguineous to some extent. ‘Heunisch Weiss’, ‘Blank Blauer’, ‘Plavac Mali’ and ‘Bombino Bianco’ share by descent haplotypes that are identical across between 23.8% and 31.7% of their diploid genome length in all six pairwise comparisons. In particular, the members of the pair ‘Heunisch Weiss’ and ‘Blank Blauer’ and the members of the pair ‘Plavac Mali’ and ‘Bombino Bianco’ share by descent 31.7% and 29.3%, respectively, of their diploid genome length.

**Fig. 5:**
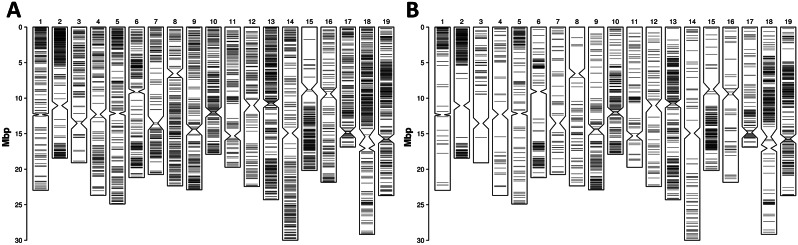
Chromosomal distribution of unmatching SNPs in the proposed trio ‘Primitivo’ x ‘Dobričić’ → ‘Plavac Mali’ in non-overlapping windows of 200 Kb of non-repetitive DNA. **A** Data from reduced-representation genome sequencing. **B** Data from whole genome sequencing from Dong and colleagues [1]

Using SPET sequencing data and the analytical pipeline presented in this article, Mendelian inconsistencies have low frequencies and are randomly distributed across the genome in *bona fide* parent-offspring trios. The incidence of Mendelian inconsistencies was higher and the chromosomal distribution was uneven for the proposed parent-offspring trio that had been claimed for explaining the origin of ‘Plavac Mali’ [41]. ‘Plavac Mali’ is the most widely planted red cultivar in Dalmatia and its pedigree was particularly intriguing because it seemed to result from a cross between two locally grown cultivars ‘Tribidrag’ and ‘Dobričić’ based on microsatellite DNA allele sizes. In the present study, IBD analysis of the proposed parentage of ‘Plavac Mali’ showed 1.7% Mendelian inconsistencies. Unmatching SNPs densely clustered on several chromosomal regions, thereby representing intervals of IBD0 that were incompatible with their origin from either ‘Tribidrag’ or ‘Dobričić’ (Fig. 5A). We validated this finding using WGS data that became publicly available recently (Fig. 5B). A percentage of 1.2% of the 472,828 SNPs that were informative in all three individuals with respect of the reference genome revealed Mendelian inconsistencies across the same chromosomal regions shown by reduced-representation genome sequencing data. WGS data show a higher saturation of variant sites and a lower background noise when compared to reduced-representation genome sequencing data. This allowed us to estimate that 19 chromosomal regions in ‘Plavac Mali’, involving 12 out of 19 chromosomes and covering cumulatively 23.3% of haploid genome, are incompatible with being inherited from either ‘Tribidrag’ or ‘Dobričić’. While we could confirm that ‘Plavac Mali’ has a parent-offspring relationship with both ‘Tribidrag’ and ‘Dobričić’, we can exclude that both ‘Tribidrag’ and ‘Dobričić’ are the parents of ‘Plavac Mali’. Pioneering microsatellite-based parentage analysis suggested that allele sharing at 25 out of 25 loci was consistent with ‘Plavac Mali’ being the progeny of ‘Tribidrag’ and ‘Dobričić’ [41]. Unfortunately, none of the microsatellite loci available at that time was located in the chromosomal segments that we show here carry incompatible SNP haplotypes with the proposed pedigree (Figure S34A). Later on, an extended microsatellite-based parentage analysis showed Mendelian inconsistencies at 4 out of 36 loci, which undermined the confidence in the hypothesis that ‘Plavac Mali’ is the progeny of ‘Tribidrag’ and ‘Dobričić’ [16]. While this incidence of Mendelian inconsistencies is not exceptionally high in microsatellite analyses of grapevine genuine trios for the reasons mentioned in the introduction section, we show here (Fig. S34B) that the alleles of ‘Plavac Mali’ that do not match the size of those in either parent are present at microsatellite loci that lie in 3 out of the 19 chromosomal segments that carry incompatible SNP haplotypes with the initially presumed pedigree [41].

‘Plavac Mali’ showed 8 unoriented parent-offspring relationships in our dataset (Fig. 4), beside those with ‘Tribidrag’, ‘Dobričić’, and three cultivars for which the direction of the relationship was resolved. We therefore tested each individual of those 8 ones (Fig. 4) and their compatibility with being the parent of ‘Plavac Mali’ in a parental combination with either ‘Tribidrag’ or ‘Dobričić’. None of these combinations could explain the origin of ‘Plavac Mali’ (Table S7). In a similar way, we tested each individual of the 5 ones that showed an unoriented parent-offspring relationships with ‘Tribidrag’ (Fig. 4), their compatibility with being the parent of ‘Tribidrag’ in a parental combination with ‘Plavac Mali’. None of these combinations could explain the origin of ‘Tribidrag’.

### Effectiveness and efficiency of SPET sequencing in GWAS

SPET sequencing made available 306,965 SNPs with MAF > 0.01 in the panel of 84 accessions that we selected for GWAS. This amount corresponded to an average frequency of one SNP every 68 informative base pairs, with the genomic distribution of shown in Fig. S35. This SNP dataset tagged 23,703 transcriptional units (15% of all SNPs are located in the immediate vicinity of the 5’ or the 3’ end of the transcriptional unit at a median distance of 106 bp from the predicted transcript), which represent 74.4% of all predicted genes, with a median value of 9 SNPs per gene. Another 3,271 genes were targeted by the SPET primers, but no SNP was detected across those regions in the panel of 84 accessions (Fig. S35). Considering all genes in the reference genome, the frequency at which more than two consecutive genes remained untagged by any SNP was negligible (2.7%). For 7.4% of the genes, the nearest SNPs were located in the next flanking genes on both sides of the untagged gene. For 5.4% of the genes, the nearest SNPs were located in at least one of the flanking genes next to the untagged gene. In addition to the density and distribution of SNP genotyping, the genetic composition of the species under study, and thereby the linkage disequilibrium between SNPs in the GWAS panel, is another major factor affecting detection power and mapping resolution. In this respect, median *r*^*2*^ between each SNP and other SNPs within a maximum distance of 200 Kb is low across the genome, with the notable exception of the domestication locus on chromosome 17 at around 6 Mbp [12, 15] (Fig. S36), suggesting that the residual effect of kinship is limited in the analysed subset of accessions. However, box plot distributions showed an abundance of outliers represented by higher *r*^*2*^ values on all chromosomes (Fig. S36), suggesting the presence of haplotype blocks, which is a positive factor for providing detection power with sparse SNP genotyping and a negative factor for high-resolution mapping.

### Berry colour

**Fig. 6:**
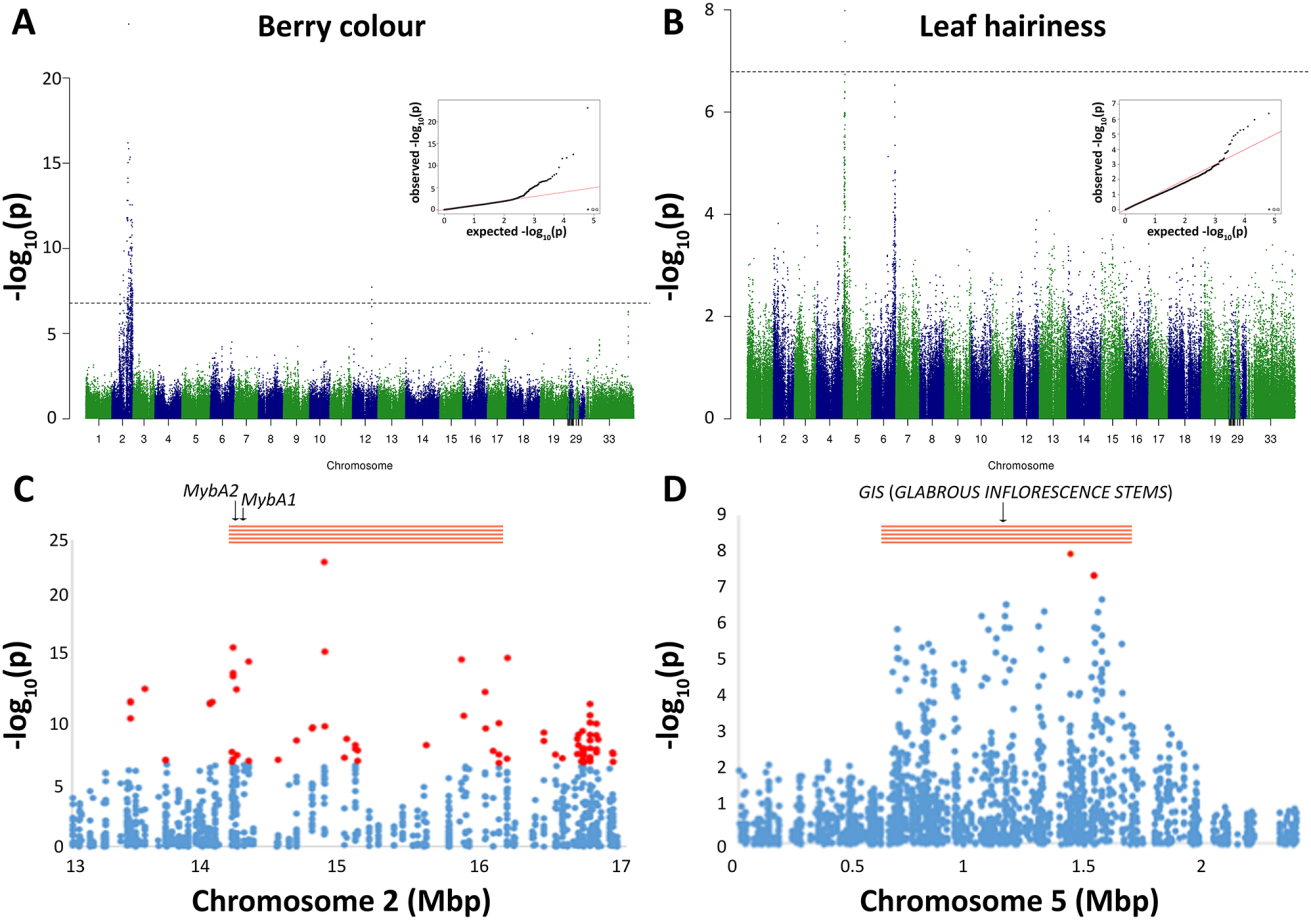
GWAS for berry colour and density of prostrate hairs on the abaxial leaf lamina (descriptor OIV 084). Manhattan plots of − log_10_(p values) vs. chromosomal positions (**A**, **B**), including unanchored scaffolds, and Q–Q plots (insets) of 306,965 SNP markers. Dashed line represents significance threshold. **C**-**D** Magnification of the genomic regions with associated SNPs (red dots)

The Manhattan plot in Fig. 6A shows 143 SNPs above the significance threshold that are associated with berry colour, 140 of which are located on chromosome 2. Of these (Table S8), 139 SNPs span an interval of approximately 8.4 Mbp, which is centered on the array of *MybA* genes that is known to encode two transcription factors (MybA1 and MybA2, Fig. 6C) controlling the expression of a key structural gene for anthocyanin biosynthesis [42]. The large size of the genomic region over which SNP-trait association persists above statistically significant levels is due to high *r*^*2*^ between SNP pairs at Mbp-sized physical distances (Fig. S37), presumably resulting from the low number of recombination events that have historically occurred between the white haplotype and red haplotypes, especially downstream of the *MybA* array [12]. One SNP outside that interval is located at Chr2:7,159,705 in the *VIT_202s0012g01140* gene, which has been annotated as a BSK2-like serine/threonine protein kinase. Three associated SNPs are located on chromosome 12 of the reference genome in a short interval that is highly similar in nucleotide sequence to an interval on chromosome 2 that is located at 16.7 Mbp, within the region shown in Fig. 6C, where several associated SNPs are in linkage disequilibrium with the *MybA* genes. The latter may be considered as a kind of false association, because true positive SNP-trait associations revealed by the reads are displaced from the correct genomic location due to read misalignments on the reference genome.

### Leaf hairiness

**Fig. 7:**
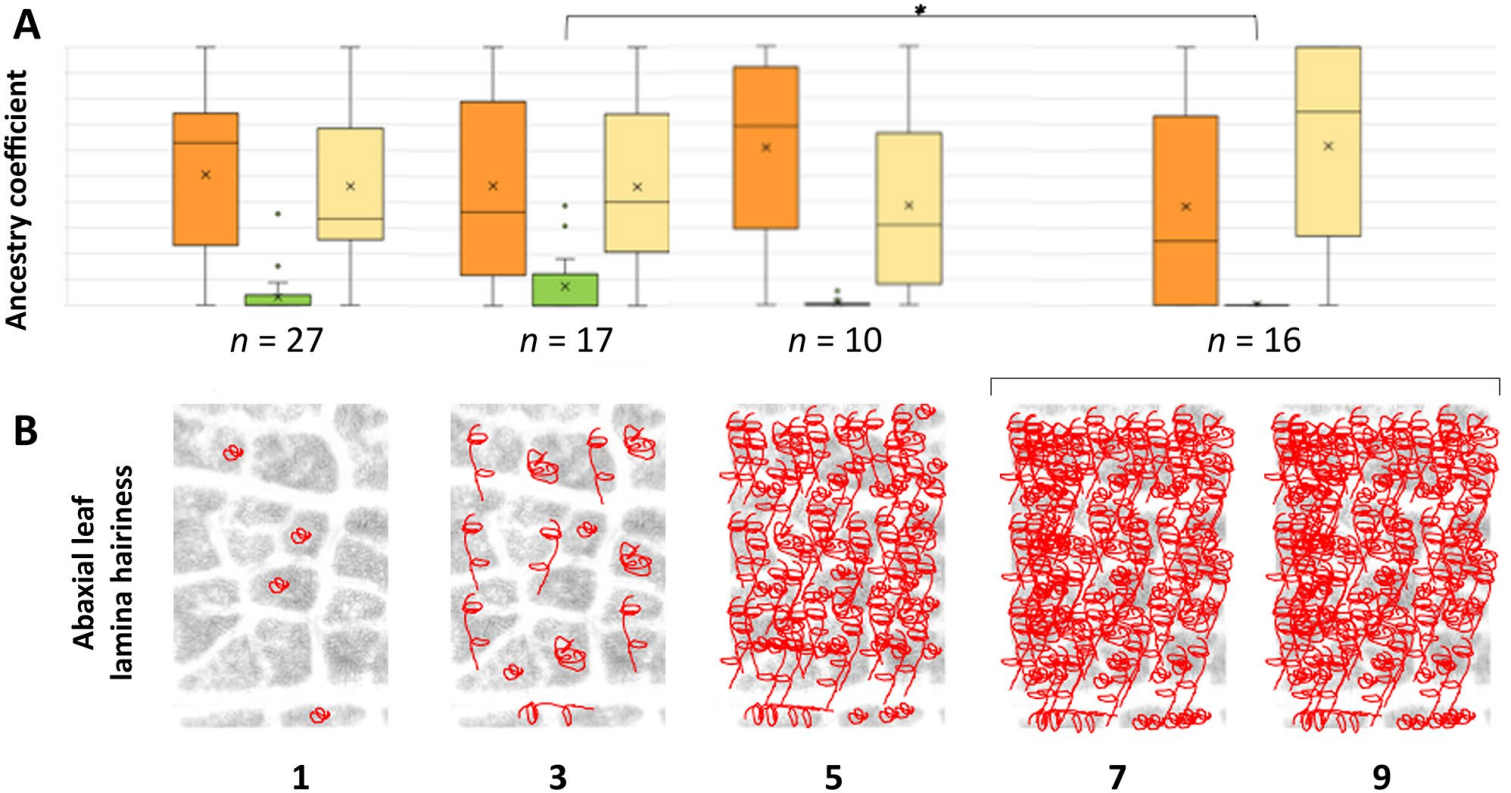
**A** Box plot distribution of ancestry components in cultivars sorted into OIV 084 phenotypes for the density of prostrate hairs between main veins on the abaxial leaf lamina. Orange bars represent Plavac Mali/Bombino Bianco *sativa* ancestry. Yellow bars represent Heunisch Weiss/Blank Blauer *sativa* ancestry. Green bars represent *sylvestris* ancestry. *n* indicates the number of cultivated accessions in each category. **B** Cartoons provide a representation of hairiness levels for defining the phenotypic classes in the present work, according to the OIV 084 drawings in 2nd Edition of the OIV Descriptor List for Grape Varieties and *Vitis* Species [35]

Two SNPs on chromosome 5 were significantly associated with the density of prostrate hairs between the main veins on the abaxial leaf lamina (Fig. 6B). This trait is also known in viticulture as ampelographic descriptor OIV 084 [35]. The first SNP (A/T) is located at Chr5:1,424,308, in an exon of the gene *VIT_205s0077g01800* that encodes for a protein with no predicted function and no known protein domains, according to NCBI and InterPro databases [43]. The second SNP (T/A) is located at Chr5:1,525,871 in an exon of the gene *VIT_205s0077g01940* that is predicted to encode an IBR3 acyl-CoA dehydrogenase, which oxidizes Indole-3-butyric acid (IBA) into indole-3-acetic acid (IAA) in auxin biosynthesis [44]. However, given the occurrence of long-range correlation (*r*^*2*^) between distantly located SNPs within this region (Fig. S38), it is unlikely that exactly those genes may have a role in explaining the observed phenotypic variation. Figure 6D shows, indeed, an interval of approximately 1 Mbp that is likely to contain the causal factor. No other OIV descriptor showed statistically significant associations.

as leaf hairiness might be an adaptive trait and the chromosomal plot of *r*^*2*^ distribution showed a localized increase of linkage disequilibrium at one edge of the genomic region identified on chromosome 5 (Fig. S36), we sought to investigate possible relations between leaf hairiness and ancestry components. When we sorted cultivars in categorical phenotypes (i.e. the only one assigned to OIV 084 class 9 was merged to those assigned to class 7 in the following analysis), we did not detect differences in the distribution of *sativa* ancestry components among hairiness categories, but we found an inverse trend of decreasing *sylvestris* ancestry component as hairiness increased (Fig. 7). In particular, the distributions of the *sylvestris* ancestry component were significantly different (*p* = 0.041) at a two-sided Wilcoxon test between cultivars with hairiness class 3 and cultivars with hairiness classes 7–9.

## Discussion

There is a long-lasting debate in viticulture as to what extent local grapevines are autochthonous and how much they differ in ancestry and genealogy from those in neighboring countries. The results of the present study add food for thought about these points.

The term autochthonous is borrowed from ecology and anthropology. The use of this term has become particularly popular in viticulture to indicate cultivars that have a special link with a vine-growing area—this link tracing far back into the past. In the process by which the term autochthonous from the original context was adapted for use in viticulture, it has acquired multifaceted meanings that are not semantically interchangeable when referring to European grapevine cultivars. Taking for granted that grapevine domestication has not occurred in Europe and domesticated germplasm was introduced from elsewhere, which is not challenged by the results of this study, the term autochthonous in a strict sense implies that indigenous ancestry in European cultivars, if present, may only derive from introgression from indigenous populations of *sylvestris*. In this respect, Croatian cultivars did not show genomic signatures of *sylvestris* introgression from local populations in agreement with earlier studies [27], which would lead to conclude that Croatian cultivars are not direct descendants of local *sylvestris* populations. Western Europe cultivars, such as ‘Chasselas Blanc’ and ‘Cabernet Sauvignon’, and Central-Eastern Europe cultivars, such as ‘Welschriesling’, which were introduced in Croatia from elsewhere, have higher European *sylvestris* ancestry than local cultivars [15, 28]. The only exception is represented by ‘Divjaka’, which could, however, either have inherited *sylvestris* ancestry through Western Europe cultivars or be the result of a recent hybridization with local *sylvestris*. Our analysis could not resolve this level of detail, which would require the availability of multiple, unrelated and undisturbed populations of European *sylvestris* that never received gene flow from *sativa*. The term autochthonous is also used in a broader sense to acknowledge that a cultivar (i.e. precisely the seedling) originated in the geographical area where it is currently grown, no matter the ancestry and the origin of their parents. In this respect, which takes in greater consideration historical circumstances rather than population genetics, we found in Croatia two large descent groups among local cultivars, one originating from ‘Heunisch Weiss’ and/or ‘Blank Blauer’, the other one originating from ‘Plavac Mali’ and/or ‘Bombino Bianco’. The fact the so many seedlings of these progenitors are present only locally makes it more likely that at least part of them originated in this geographical area rather than they were all introduced from other areas from which they have later disappeared. This hypothesis is reinforced by the evidence that descendants of ‘Heunisch Weiss’ and/or ‘Blank Blauer’ are typically grown in Continental Croatia and northern Adriatic Croatia, where their parents are also found, and descendants of ‘Plavac Mali’ are typically grown in Dalmatia, along with their parent (Fig. S1). An exception is represented by descendants of ‘Bombino Bianco’ that are typically grown in Dalmatia, although ‘Bombino Bianco’ is not cultivated there and is only grown in the Istrian peninsula under the synonym of ‘Trevolina Istriana’ [45].

This brings us into the second point of discussion: how is grapevine diversity hierarchically interconnected country-wise and Europe-wise? It comes as no surprise that ‘Heunisch Weiss’ has several descendants and relatives in Croatia, as this genotype has by far the highest number of parent-offspring relationships across the Balkans, Northeastern Italy, Eastern and Central Europe. No wonder either that ‘Blank Blauer’ is a common parent in Continental Croatia, northern Adriatic Croatia, as it has several parent-offspring relationships with grapevines in Austria, Hungary and on the northern Adriatic shores of North-Eastern Italy [24], including the cultivar ‘Glera’ that is used for the production of the Prosecco sparkling wine. It is also noteworthy that ‘Visparola’, a cultivar among those that have a parent-offspring relationship with ‘Blank Blauer’, is itself a major progenitor in Central Italy [24]. ‘Bombino Bianco’ deserves more attention. In Dalmatia, there are no historical records of ‘Bombino Bianco’ [46], where it was once mistaken for the cultivar ‘Debit’ (synonym of ‘Puljižanac’). ‘Bombino Bianco’ is extensively grown on the Adriatic shores of Central Italy, where is also known under the synonym of ‘Passerina’. D’Onofrio and colleagues [24] discovered that ‘Bombino Bianco’ has parent-offspring relationships with 11 Italian cultivars, in addition to the 11 Croatian cultivars identified in our study. It remains therefore impossible to infer from historical and genetic data whether ‘Bombino Bianco’ was a popular cultivar once grown across the entire Adriatic Sea Basin and it gave birth to descendants everywhere or its descendants were introduced from a smaller area where they originated, which is not necessarily corresponding to the present-day distribution of ‘Bombino Bianco’.

At a larger geographical scale, data show that there is a common and narrow genetic basis of the germplasm today found in the Adriatic Sea Basin. The imprint of this genetic basis extends northwards into Central Europe and eastwards into the Danube Basin. Nonetheless and in contrast with the general rule of high consanguinity, it was striking to notice that no parent-offspring relationship was found between the two most widely grown Croatian white cultivars, ‘Welschriesling’ and ‘Malvazija Istarska’, and the rest of Croatian germplasm, although these genotypes share the same ancestry components with the rest of the germplasm. As for ‘Welschriesling’, no parent-offspring relationship was found either in the panel of this article or in WGS panels from [12] and [1], including collectively more than 3,500 accessions. Only one parent-offspring relationship has been reported with ‘Orsolina’ [24], a minor cultivar in Northern Italy where ‘Welschriesling’ is also grown under the synonym of ‘Italian Riesling’. As for ‘Malvazija Istarska’, the only known parent-offspring relationship is represented by ‘Vega’, which was selected by the breeder Giovanni Dalmasso from an intentional cross between ‘Malvazija Istarska’ and ‘Furmint’ [24, 47]. The unique genotypic combinations in ‘Welschriesling’ and ‘Malvazija Istarska’, which may have derived from parents that are now extinct, enrich diversity by reassorting common ancestries into novel allelic combinations. PCA results are indeed coherent with the assumptions that cultivars in the Western Balkans are a relevant part of the existing diversity in Europe [12, 14, 16, 23, 48–50], that some of them played an important historical role in the generation of descent groups in other parts of Europe, and that a few of them are the result of singular, likely unrepeatable, parental combinations.

The origin of the most widely grown Croatian red cultivar, ‘Plavac Mali’ deserves a separate discussion. We conclusively exclude that ‘Plavac Mali’ is the offspring of the cross between ‘Tribidrag’ and ‘Dobričić’ [16, 17] or the result of other combinations reported in the results section. This leaves all other scenarios and speculations possible. Considering the historical records that mention the synonym ‘Tribidrag’ in the Balkans as early as in the 15th century [51, 52], it is more likely that ‘Tribidrag’ predates ‘Plavac Mali’ rather than vice versa. If that were true, it would be consequent to assume that ‘Tribidrag’ is one of the parents of ‘Plavac Mali’, and ‘Dobričić’ is an offspring of ‘Plavac Mali’. The fact that ‘Dobričić‘ is only present on the small island of Šolta, off the Central Dalmatian coast, and the fact that it has no parent-offspring relation with known cultivars other than ‘Plavac Mali’ are in favour of a distal position for ‘Dobričić’ on the family tree. The opposite hypothesis would require two assumptions to invoke. First, ‘Dobričić’ was ancestral to both ‘Tribidrag’ and ‘Plavac Mali’ and it has progressively disappeared from viticulture. Second, ‘Plavac Mali’ rose to fame long after its presumed offspring ‘Tribidrag’ had become widely known with different names in the Balkans as well as in Southern Italy. However, caution should be used with these interpretations. Although the century-old cultivation of ‘Tribidrag’ in the Balkans was confirmed by microsatellite analysis of a 90-year-old herbarium specimen [53], more ancient historical records may have associated the cultivar name with one or more different genotypes that are not corresponding to ‘Tribidrag’ as we know it nowadays. Similar cases of uncertain direction in parent-offspring relationships for important regional cultivars has led to considerable debate in the past, which is well illustrated by the case of ‘Sangiovese’. Initially, two hypotheses were proposed based on SSR alleles [54, 55] that consistently indicated ‘Ciliegiolo’ as one of the biological parents, until trio analysis based on SNP markers [24] clarified that ‘Ciliegiolo’ is an offspring and not a parent of ‘Sangiovese’.

There is also a lot of uncertainty about the status of the present-day populations of European *sylvestris* and their relations with locally grown cultivars. This indeterminateness is due to the fact that, when signs of admixture are found in both compartments, the direction of the gene flow is hard to ascertain in presence of very small population sizes on one side and lack of random mating on the other side. Data from the present article suggest the occurrence of a crop-to-wild gene flow that has not left any of the surveyed wild populations untouched. The detection in local spontaneous individuals of a domestication-related *sativa* haplotype that is extremely frequent everywhere in the cultivated germplasm leaves no room for doubt that this situation represents only the tip of the iceberg. The introgression in once wild populations of *sativa* alleles at other genes is much harder to detect in genomic analyses because of lower linkage disequilibrium and overlapping frequency spectra between the two gene pools. The introgression in once wild populations of recessive *sativa* alleles, as illustrated by the white haplotype, is not even recognized by expert prospectors, because they will be mostly present in a heterozygous condition, as in the case shown here, and will not affect the phenotypic appearance of the wild individuals (Fig. S12). Last but not least, the particular *sativa* haplotype we found out to have leaked into spontaneous populations is unlikely to have provided adaptive advantages to the recipient individuals and to have been positively selected for, which could have otherwise contributed to increase its frequency compared to random expectations. These findings are in agreement with Dong and colleagues’ WGS analysis [1], according to which Croatian *sylvestris* accessions were predominantly assigned an admixed *sylvestris*-*sativa* ancestry, with the exception of only three individuals. The impact of crop-to-wild gene flow on conservation and breeding programs of spontaneous populations might be severe. The introduction of deleterious recessive alleles and maladaptive traits, which are part of the genetic load in the cultivated compartment [11, 56], into small and partially isolated spontaneous populations may further endanger their survival and/or impoverish their reservoir of adaptive traits useful for breeding.

In this article, we also provided methodological proofs that reduced-representation genome sequencing is an effective approach for obtaining significant SNP-trait associations. The advantage of the SPET method, compared to other GBS approaches, in GWAS applications resides in the fact that it can target the gene space and investigate portions of the transcriptional unit from the vast majority of the predicted genes. In this study, we identified 2 SNPs that are associated with the density of prostrate hairs on the abaxial side of the leaf blade on the upper subtelomeric end of chromosome 5. Two QTL mapping studies consistently identified a QTL for leaf hair density over the same region [57, 58]. The latter study also proposed a functional candidate gene in the QTL region (*VIT_205s0077g01390*), based on its predicted homology with GLABROUS INFLORESCENCE STEMS transcription factors that control trichome cell development in *Arabidopsis. VIT_205s0077g01390* is located approximately 295 Kb upstream of our associated SNPs (Fig. 6D). The GWAS results suggest that the SNP dataset generated by using our SPET primer design provides adequate detection power to identify associations between sparse SNPs across large regions and phenotypes with very different characteristics. One of them was a Mendelian trait controlled by a single locus in a relatively gene-poor arm on chromosome 2 (Fig. 6A, C and Fig. S35). The other one was characterized by quantitative trait variation and polygenic inheritance, with SNP-trait associations found in a gene-rich subtelomeric region of chromosome 5 (Fig. 6B, D and Fig. S35). These results are consistent with simulations made by Nicolas and colleagues [59], who predicted that approximately 500,000 SNPs are optimal for efficient GWAS in a comprehensive grapevine diversity panel. Our SPET sequencing protocol yielded a similar number of SNPs in a smaller diversity panel that was mainly composed of European wine grapes due to the focus of this article on Croatian germplasm. Our SPET protocol also captures genomic regions for sequencing in a targeted way with a physical density that is variable in proportion to gene density. Considering these facts together, we are confident to recommend the use of this SPET protocol for cost-effective GWAS. The genetic composition of the species and the crop germplasm determines the operational boundaries within which this protocol is adequate to different GWAS purposes. On the one hand, the long-range correlation between SNP genotypes that are located hundreds or thousands Kb apart from one another facilitates the identification of locus-trait associations with low investment in sparse SNP genotyping (Fig. S37-S38). On the other hand, high linkage disequilibrium hampers high-resolution mapping, which is a necessary for narrowing SNP-trait associations down to individual genes. This limiting condition is inherent to the evolutionary processes that have forged the species and the crop as we know it nowadays and is not going to be ameliorated by resorting to higher investments in dense SNP genotyping.

## Conclusions

The genetic separation between Croatian cultivars and *sylvestris* excludes that cultivars originated from independent events of local domestication. On the other hand, the evidence of a crop-to-wild gene flow, leading not only to obvious cases of admixture but also to more cryptic *sativa* introgression, raises an alarm on the genetic integrity of natural populations of the wild progenitor, in Croatia, in Europe and elsewhere. The results of our study also show country-wise and Europe-wise interconnections of the Croatian grapevine germplasm and stimulate the incorporation of local spontaneous genetic resources into breeding programs for improving adaptation and expanding diversity.

## Electronic supplementary material

Below is the link to the electronic supplementary material.

Additional file 1: Supplementary Tables S1 to S8

Additional file 2: Supplementary Figures S1 to S38

## Data Availability

Raw reads are deposited in the NCBI Sequence Read Archive under the BioProject number PRJNA1126049. WGS raw reads were retrieved from the BioProject numbers PRJNA373967, PRJNA390884, PRJNA385116, PRJNA321480 and from the Genome Warehouse in the National Genomics Data Center, China National Center for Bioinformation accession number CRA006917. The plant material of the 108 cultivars used in this study is held at three Croatian germplasm repositories: Institute of Adriatic Crops and Karst Reclamation, Split, institute code HRV048; Institute of Agriculture and Tourism, Poreč, institute code HRV050; University of Zagreb, institute code HRV041.

## Abbreviations

SNP: Single Nucleotide Polymorphism
QTL: Quantitative Trait Locus
SSR: Simple Sequence Repeat
NGS: Next Generation Sequencing
GBS: Genotyping by Sequencing
SPET: Single Primer Enrichment Technology
WGS: Whole Genome Sequencing
GWAS: Genome Wide Association Study
IBS: Identity by State
IBD: Identity by Descent
PCA: Principal Component Analysis
EMMAX: Efficient Mixed-Model Association eXpedited
