# Supplementary material for: Single nucleotide polymorphism (SNP) analysis reveals ancestry and genetic diversity of cultivated and wild grapevines in Croatia
Source: BMC Plant Biol. 2024 Oct 17;24:975. doi: 10.1186/s12870-024-05675-4 (PMC11483961; doi:10.1186/s12870-024-05675-4)
Supplement: Supplementary file 2 — Additional file 2: Supplementary Figures S1 to S38 [file 12870_2024_5675_MOESM2_ESM.pdf]

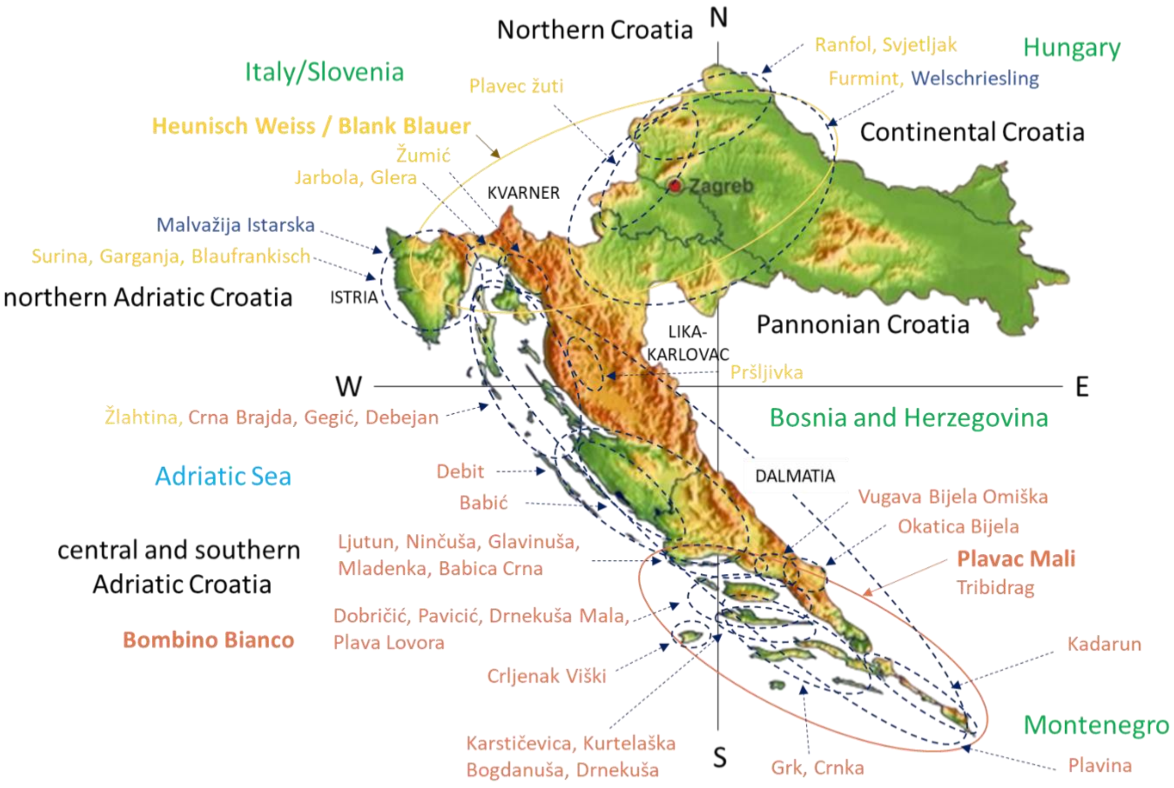

Fig. S1 Present-day area of cultivation in Croatia of the main cultivars discussed in this article and of the progenies of the four main progenitors 'Heunisch Weiss', 'Blank Blauer', 'Bombino Bianco' and 'Plavac Mali'. Progenies of 'Heunisch Weiss' and/or 'Blank Blauer' are indicated with the same colour as their parents. Progenies of 'Bombino Bianco' and/or 'Plavac Mali' are indicated with the same colour as their parents. 'Bombino Bianco' is not substantially cultivated in Croatia. Definitions of geographical areas in this article are adapted from the Nomenclature of Territorial Units for Statistics (NUTS) of the European Union, level NUTS-2. For the sake of simplicity, we refer to Northern Croatia (HR06), the City of Zagreb (HR05) and the Western part of Pannonian Croatia (HR02) collectively as Continental Croatia. We refer to Adriatic Croatia (HR03) as northern Adriatic Croatia when indicating the Istrian peninsula and coastal/insular Kvarner, as central and southern Adriatic Croatia when indicating the coastal and insular region of Lika and the entire Dalmatia. Map source: https://croatiamap360.com/croatia-geography-map.

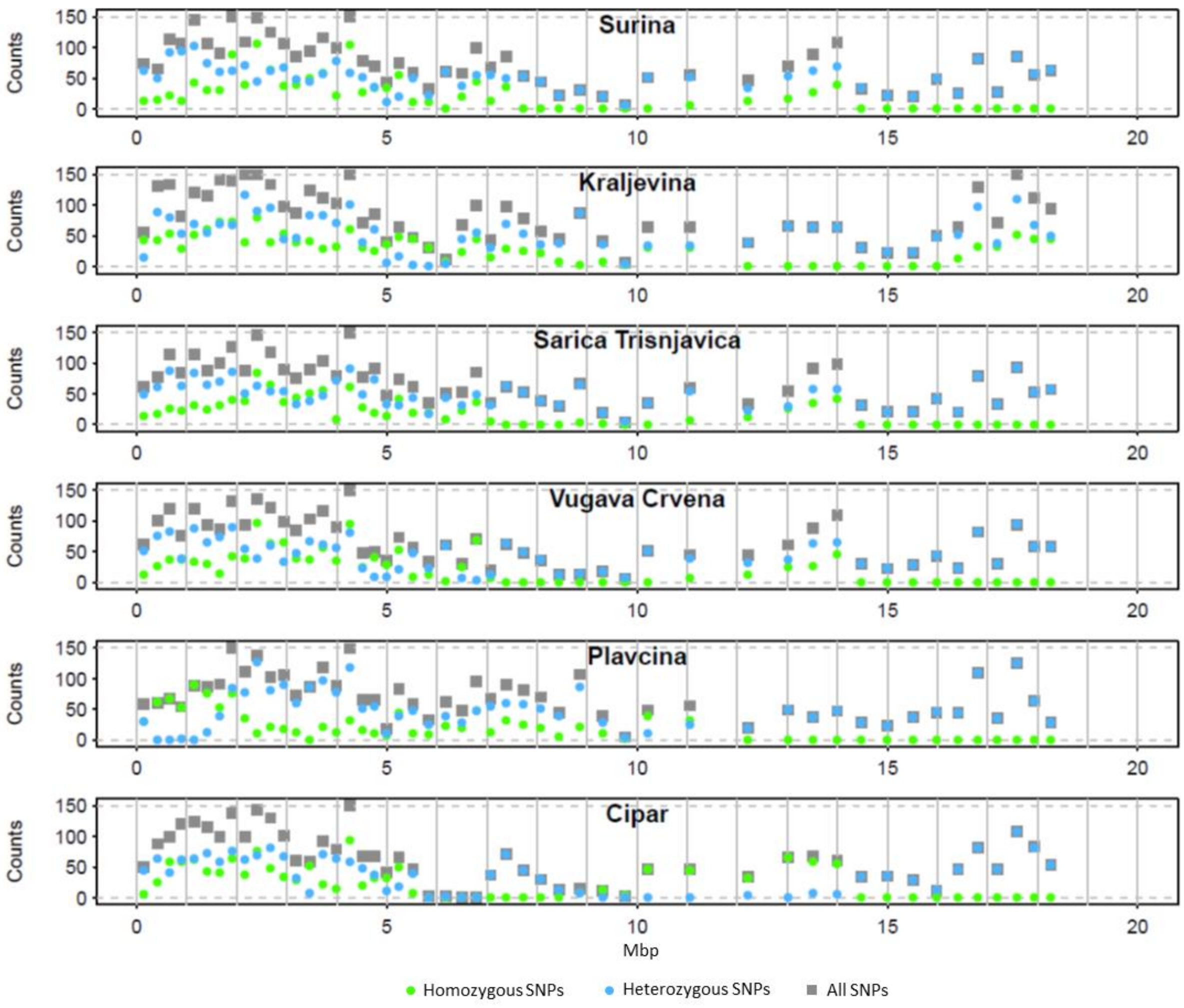

Fig. S2 Comparison between 6 cultivars of the present paper bearing light red or pink berries and the Chr2 homolog carrying the white *sativa* haplotype in the reference genome of *V. vinifera* PN40024 12Xv0 around the *MybA* array at position Chr2:14.1-14.3 Mbp. SNP density and zygosity in non-overlapping genomic windows containing 200 Kb of non-repetitive DNA. Along the y-axis, counts were plotted at a maximum value of 150 if higher.

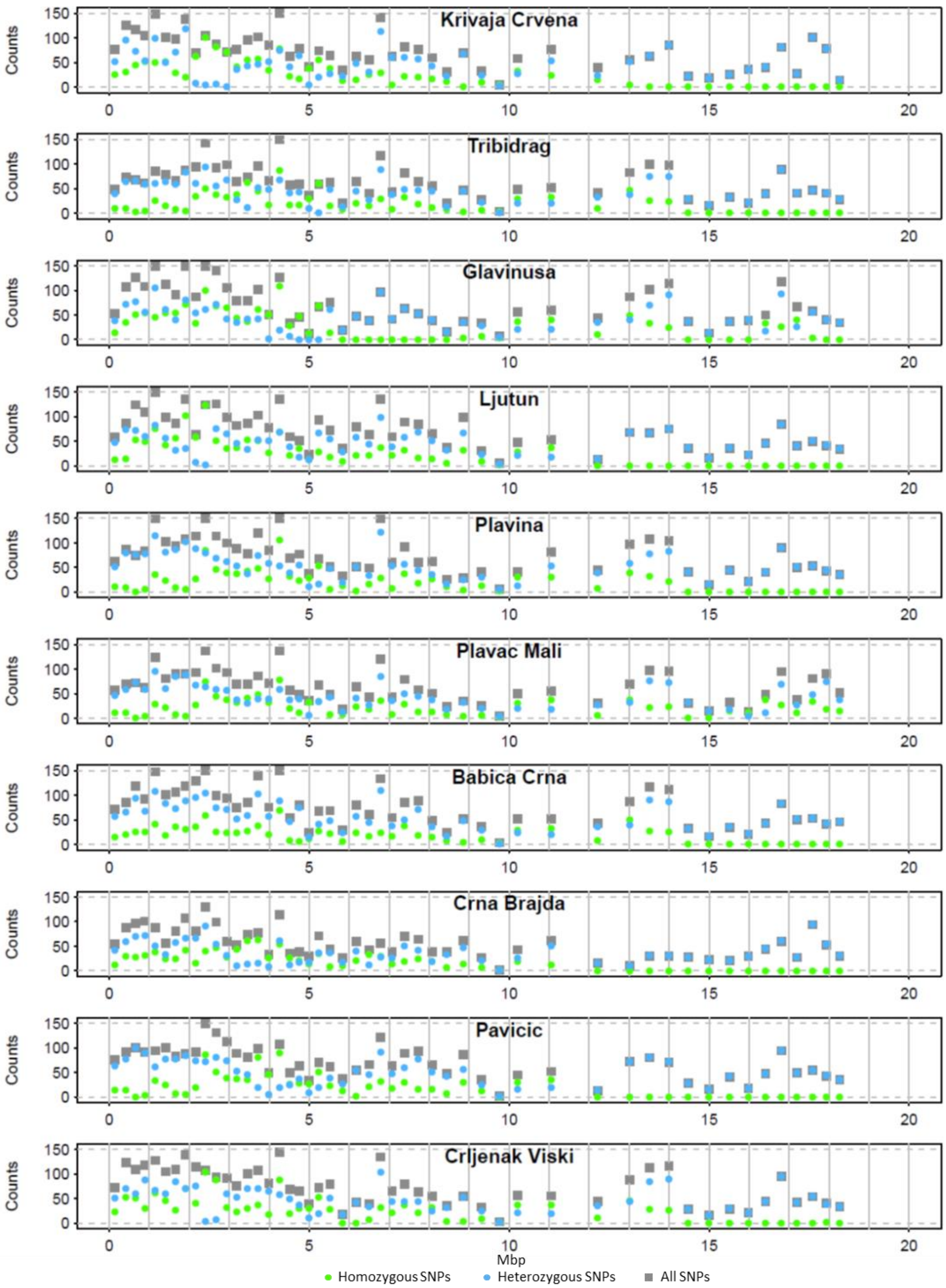

Fig. S3 Comparison between 36 cultivars of the present paper bearing dark blue or black berries and the Chr2 homolog carrying the white *sativa* haplotype in the reference genome of *V. vinifera* PN40024 12Xv0 around the *MybA* array at position Chr2:14.1-14.3 Mbp. SNP density and zygosity in non-overlapping genomic windows containing 200 Kb of non-repetitive DNA. Along the y-axis, counts were plotted at a maximum value of 150 if higher.

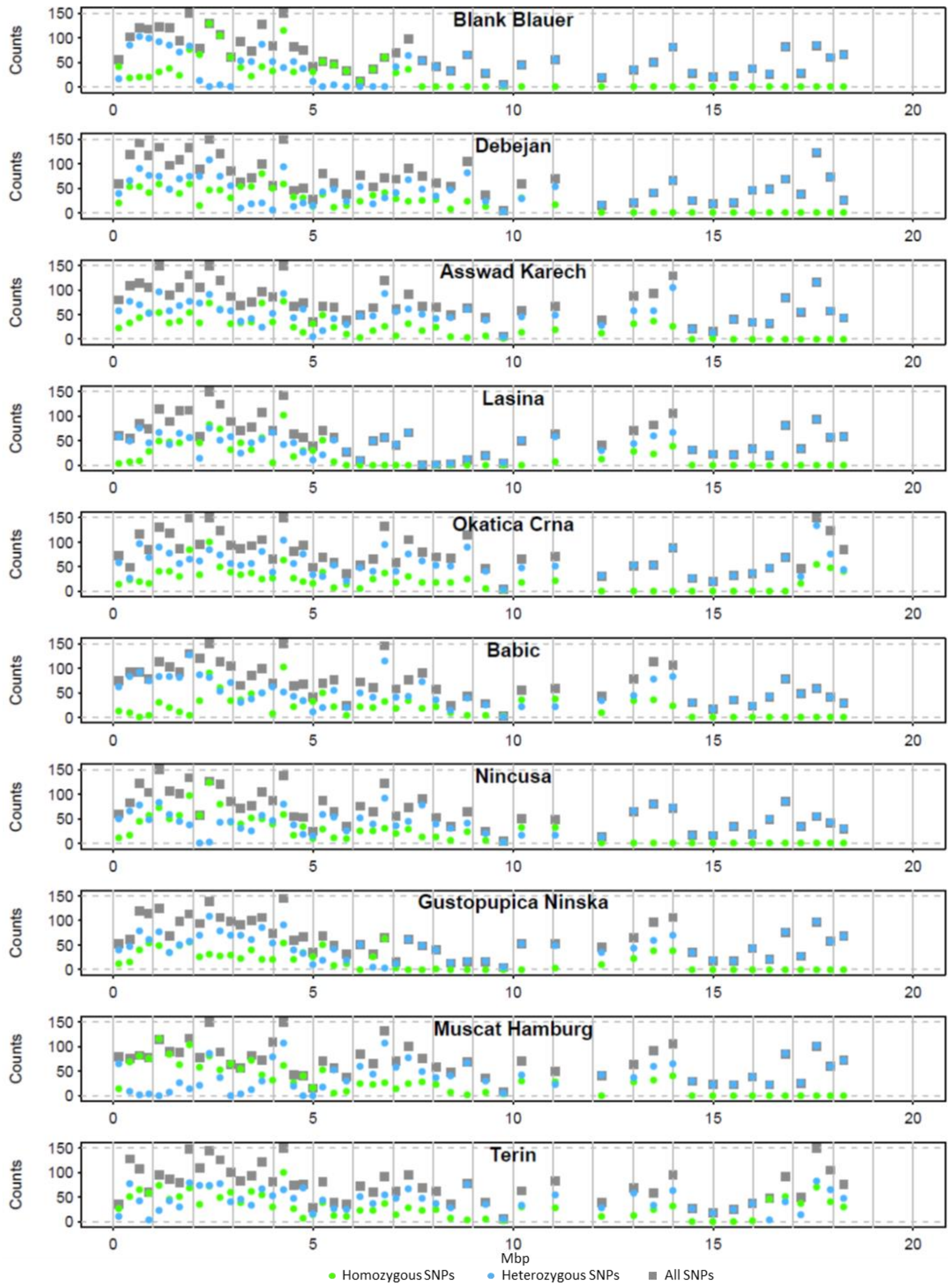

Fig. S3 (continued) Comparison between 36 cultivars of the present paper bearing dark blue or black berries and the Chr2 homolog carrying the white *sativa* haplotype in the reference genome of *V. vinifera* PN40024 12Xv0 around the *MybA* array at position Chr2:14.1-14.3 Mbp. SNP density and zygosity in non-overlapping genomic windows containing 200 Kb of non-repetitive DNA. Along the y-axis, counts were plotted at a maximum value of 150 if higher.

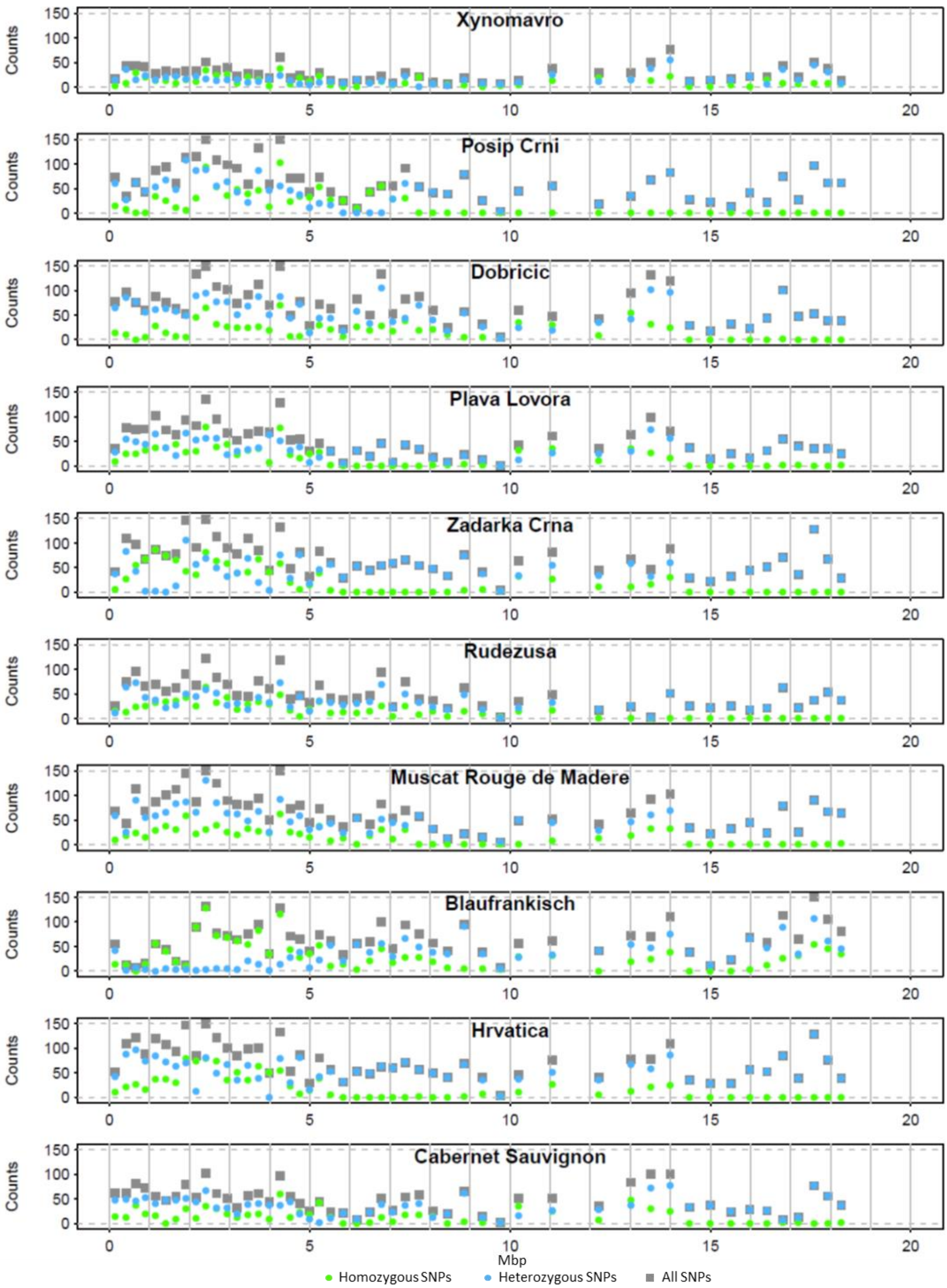

Fig. S3 (continued) Comparison between 36 cultivars of the present paper bearing dark blue or black berries and the Chr2 homolog carrying the white *sativa* haplotype in the reference genome of *V. vinifera* PN40024 12Xv0 around the *MybA* array at position Chr2:14.1-14.3 Mbp. SNP density and zygosity in non-overlapping genomic windows containing 200 Kb of non-repetitive DNA. Along the y-axis, counts were plotted at a maximum value of 150 if higher.

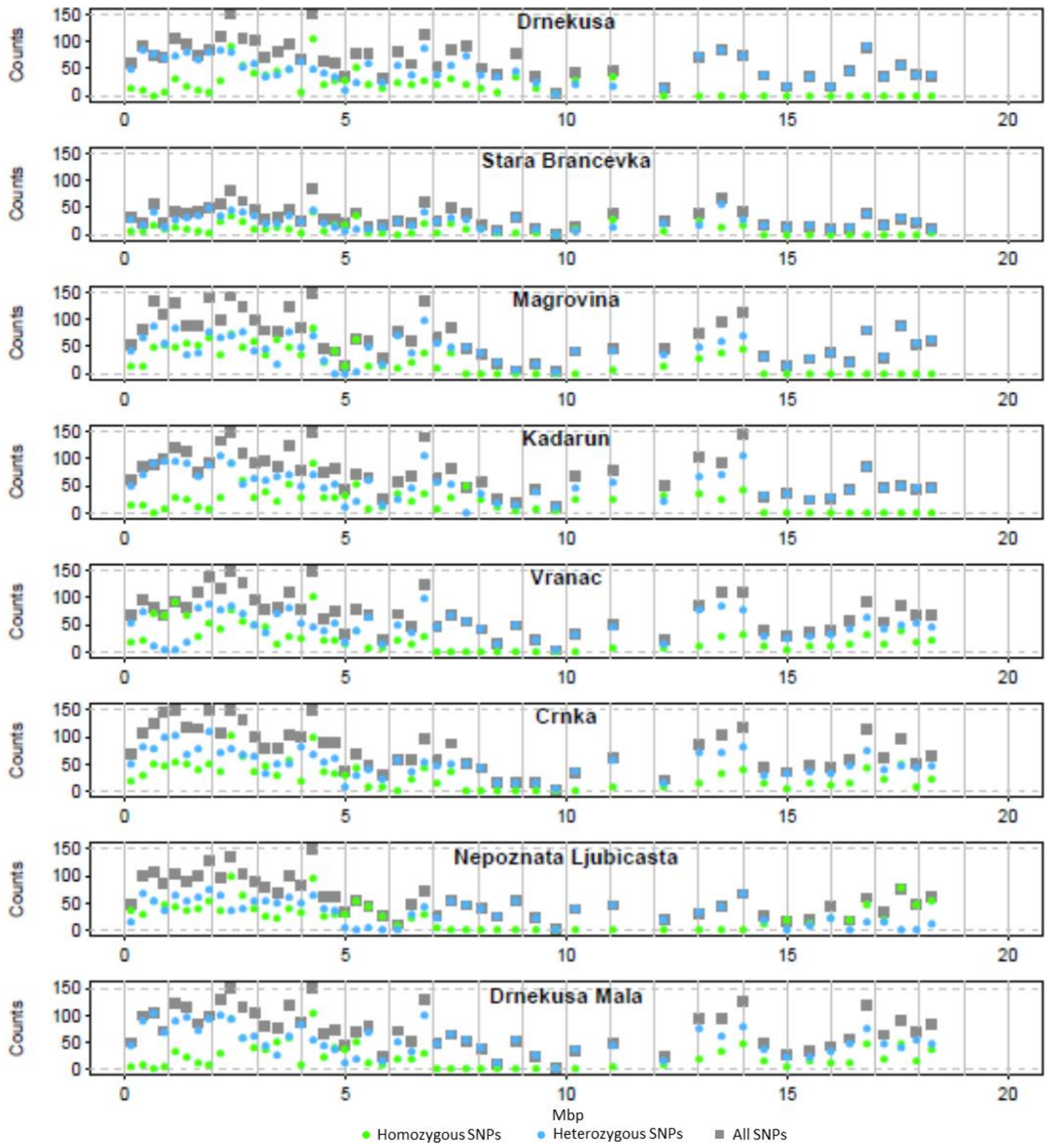

Fig. S3 (continued) Comparison between 36 cultivars of the present paper bearing dark blue or black berries and the Chr2 homolog carrying the white *sativa* haplotype in the reference genome of *V. vinifera* PN40024 12Xv0 around the *MybA* array at position Chr2:14.1-14.3 Mbp. SNP density and zygosity in non-overlapping genomic windows containing 200 Kb of non-repetitive DNA. Along the y-axis, counts were plotted at a maximum value of 150 if higher. The latter 4 cultivars (Vranac, Crnka, Nepoznata Ljubičasta and Drnekuša Mala) show a mixture of homozygous and heterozygous SNPs in the windows spanning the *MybA* array, indicating that they are carry two red haplotypes that both differ from the white *sativa* haplotype of PN40024. All other 32 cultivars show only heterozygous SNP in the windows spanning the *MybA* array and the downstream region in strong linkage disequilibrium, indicating that they are carry one copy of the white *sativa* haplotype of PN40024 in combination with a red haplotype.

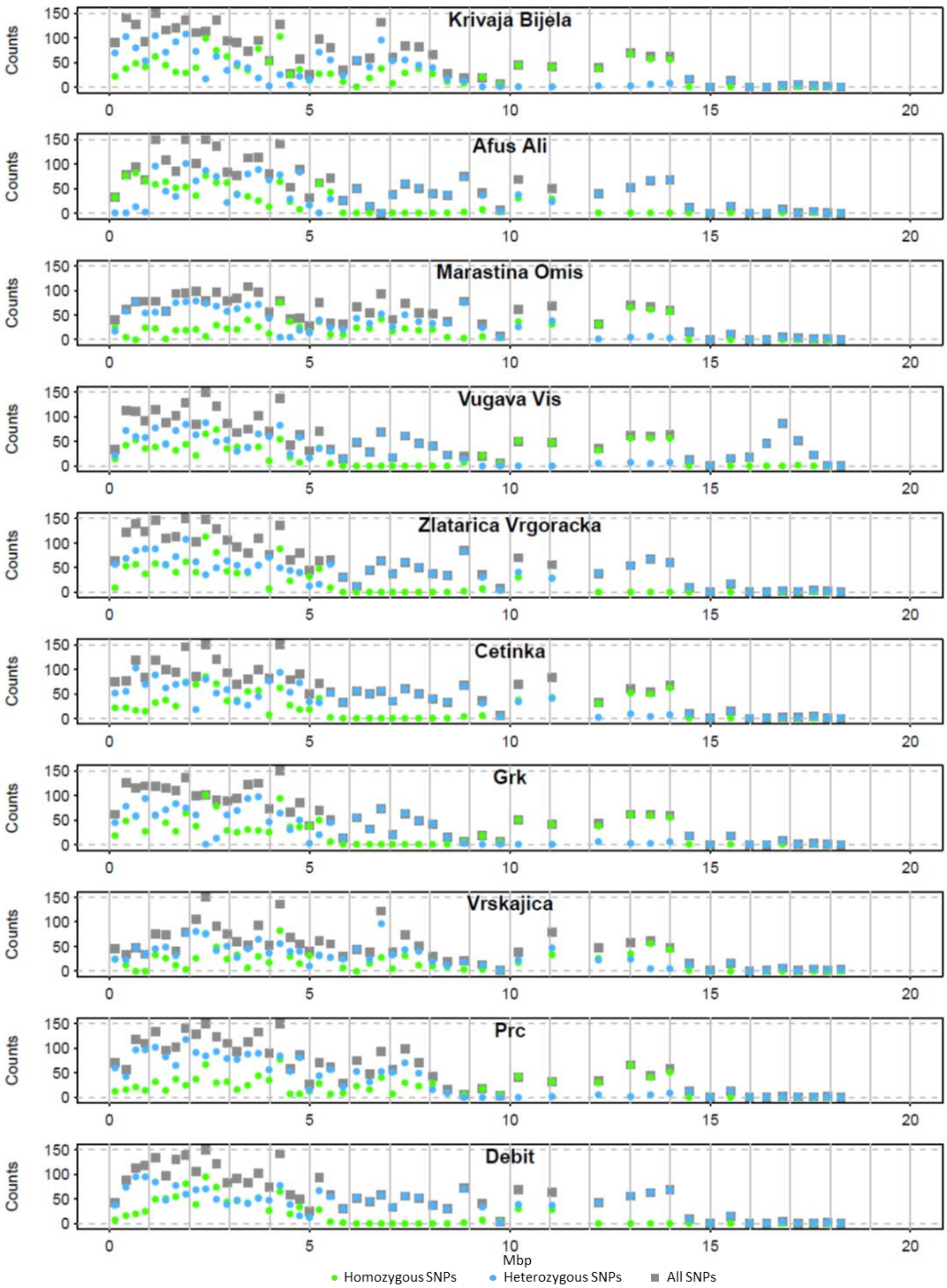

Fig. S4 Comparison between 65 cultivars of the present paper bearing yellow or green berries and the Chr2 homolog carrying the white *sativa* haplotype in the reference genome of *V. vinifera* PN40024 12Xv0 around the *MybA* array at position Chr2:14.1-14.3 Mbp. SNP density and zygosity in non-overlapping genomic windows containing 200 Kb of non-repetitive DNA. Along the y-axis, counts were plotted at a maximum value of 150 if higher.

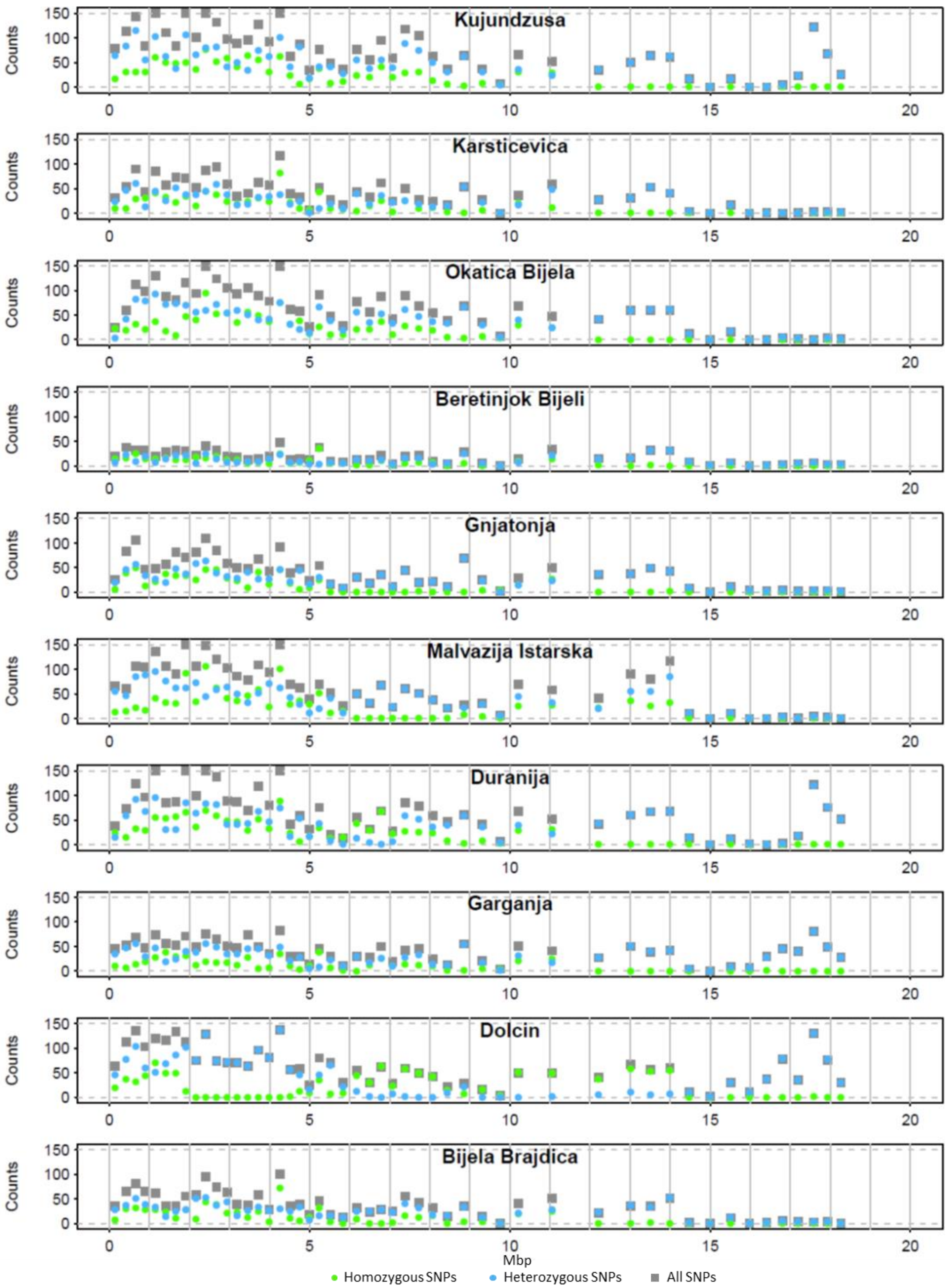

Fig. S4 (continued) Comparison between 65 cultivars of the present paper bearing yellow or green berries and the Chr2 homolog carrying the white *sativa* haplotype in the reference genome of *V. vinifera* PN40024 12Xv0 around the *MybA* array at position Chr2:14.1-14.3 Mbp. SNP density and zygosity in non-overlapping genomic windows containing 200 Kb of non-repetitive DNA. Along the y-axis, counts were plotted at a maximum value of 150 if higher.

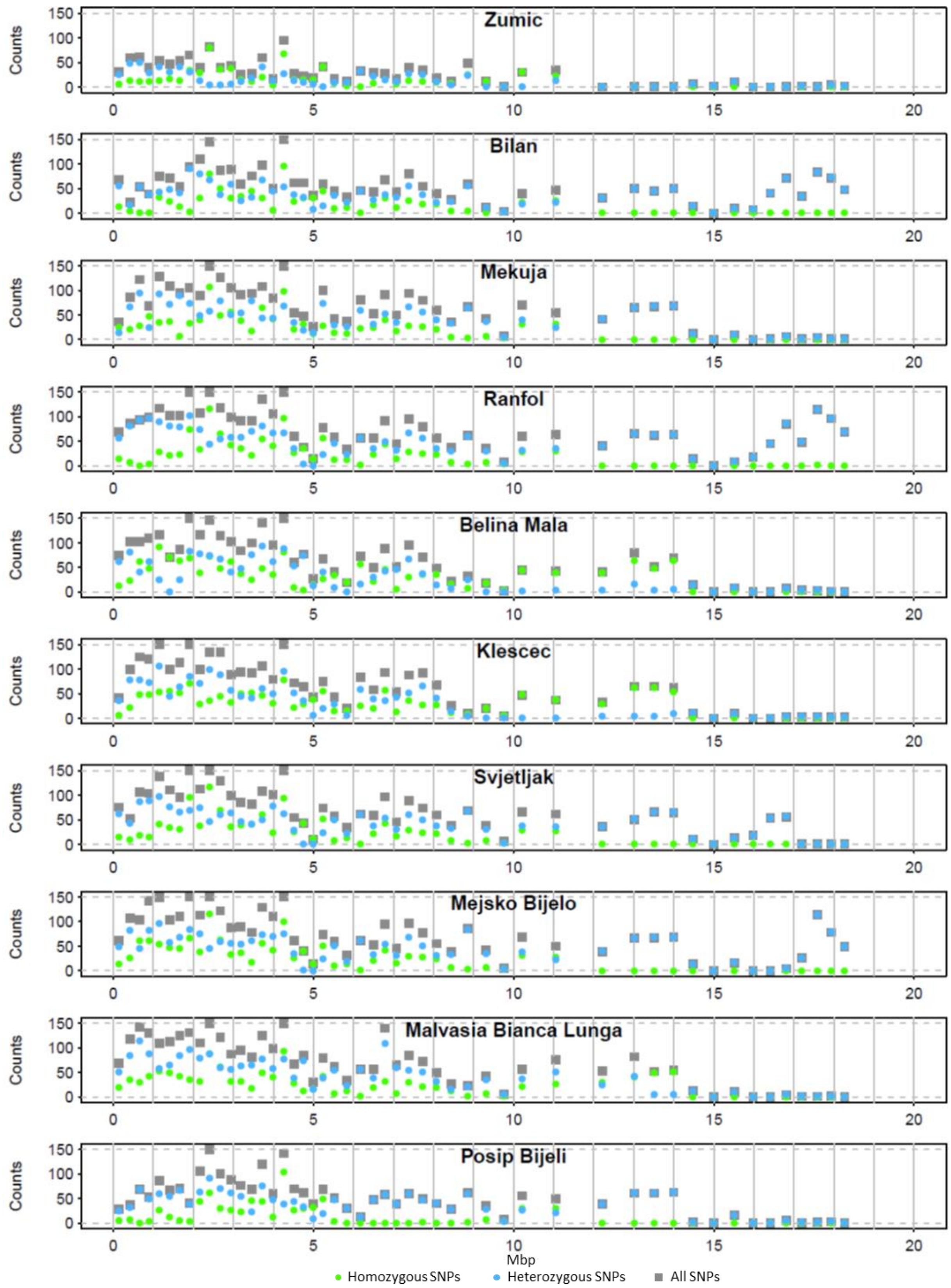

Fig. S4 (continued) Comparison between 65 cultivars of the present paper bearing yellow or green berries and the Chr2 homolog carrying the white *sativa* haplotype in the reference genome of *V. vinifera* PN40024 12Xv0 around the *MybA* array at position Chr2:14.1-14.3 Mbp. SNP density and zygosity in non-overlapping genomic windows containing 200 Kb of non-repetitive DNA. Along the y-axis, counts were plotted at a maximum value of 150 if higher.

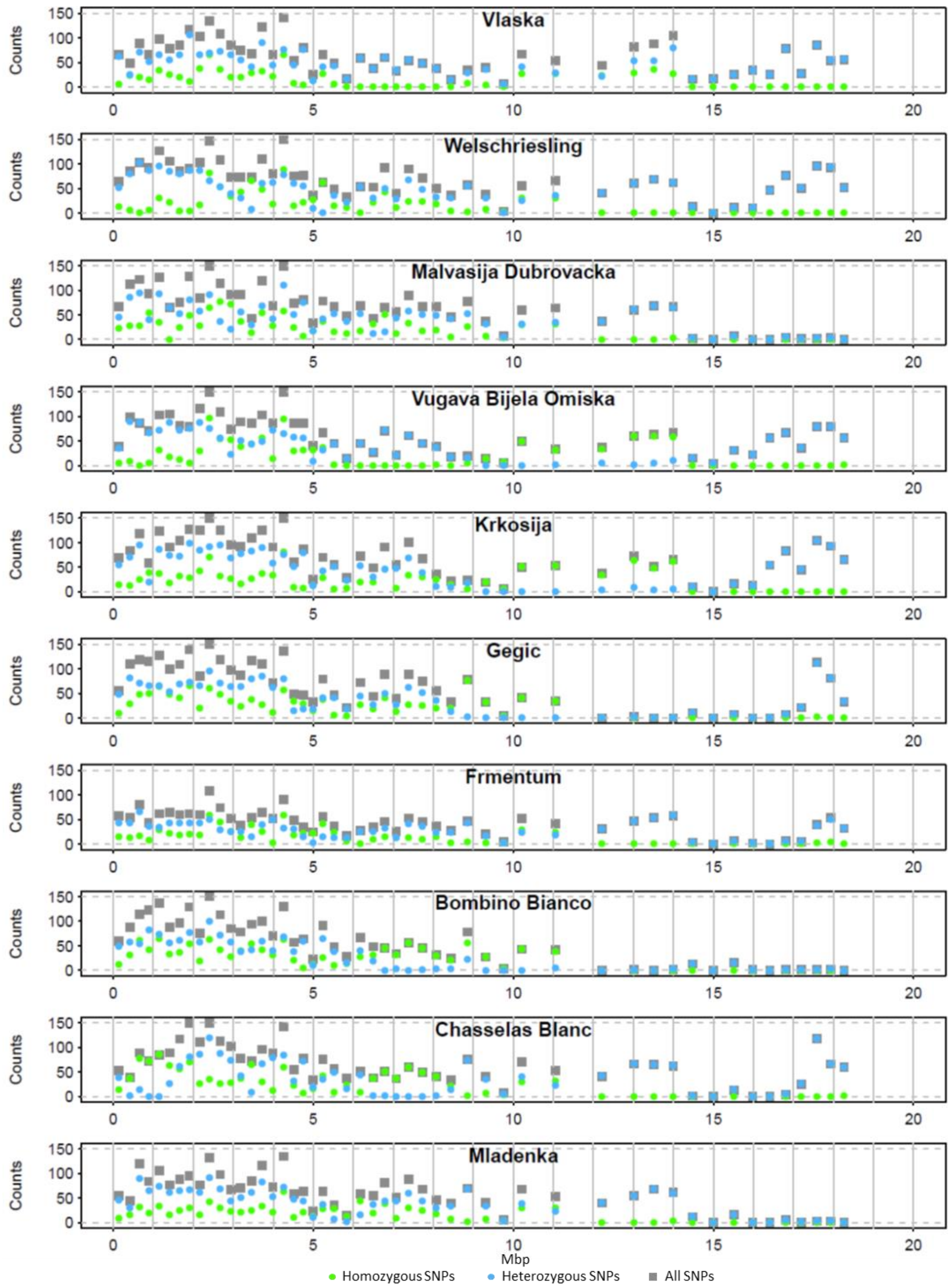

Fig. S4 (continued) Comparison between 65 cultivars of the present paper bearing yellow or green berries and the Chr2 homolog carrying the white *sativa* haplotype in the reference genome of *V. vinifera* PN40024 12Xv0 around the *MybA* array at position Chr2:14.1-14.3 Mbp. SNP density and zygosity in non-overlapping genomic windows containing 200 Kb of non-repetitive DNA. Along the y-axis, counts were plotted at a maximum value of 150 if higher.

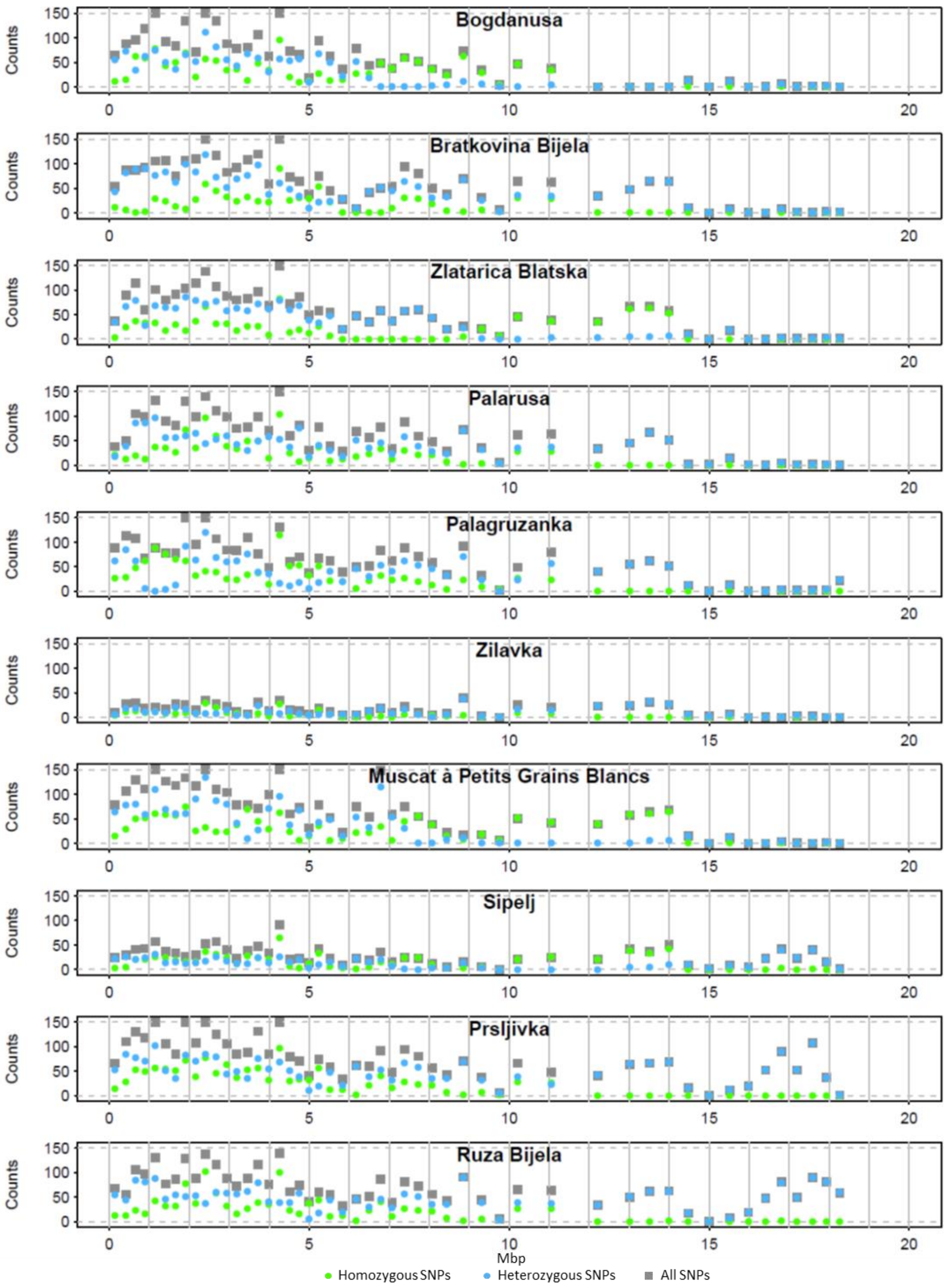

Fig. S4 (continued) Comparison between 65 cultivars of the present paper bearing yellow or green berries and the Chr2 homolog carrying the white *sativa* haplotype in the reference genome of *V. vinifera* PN40024 12Xv0 around the *MybA* array at position Chr2:14.1-14.3 Mbp. SNP density and zygosity in non-overlapping genomic windows containing 200 Kb of non-repetitive DNA. Along the y-axis, counts were plotted at a maximum value of 150 if higher.

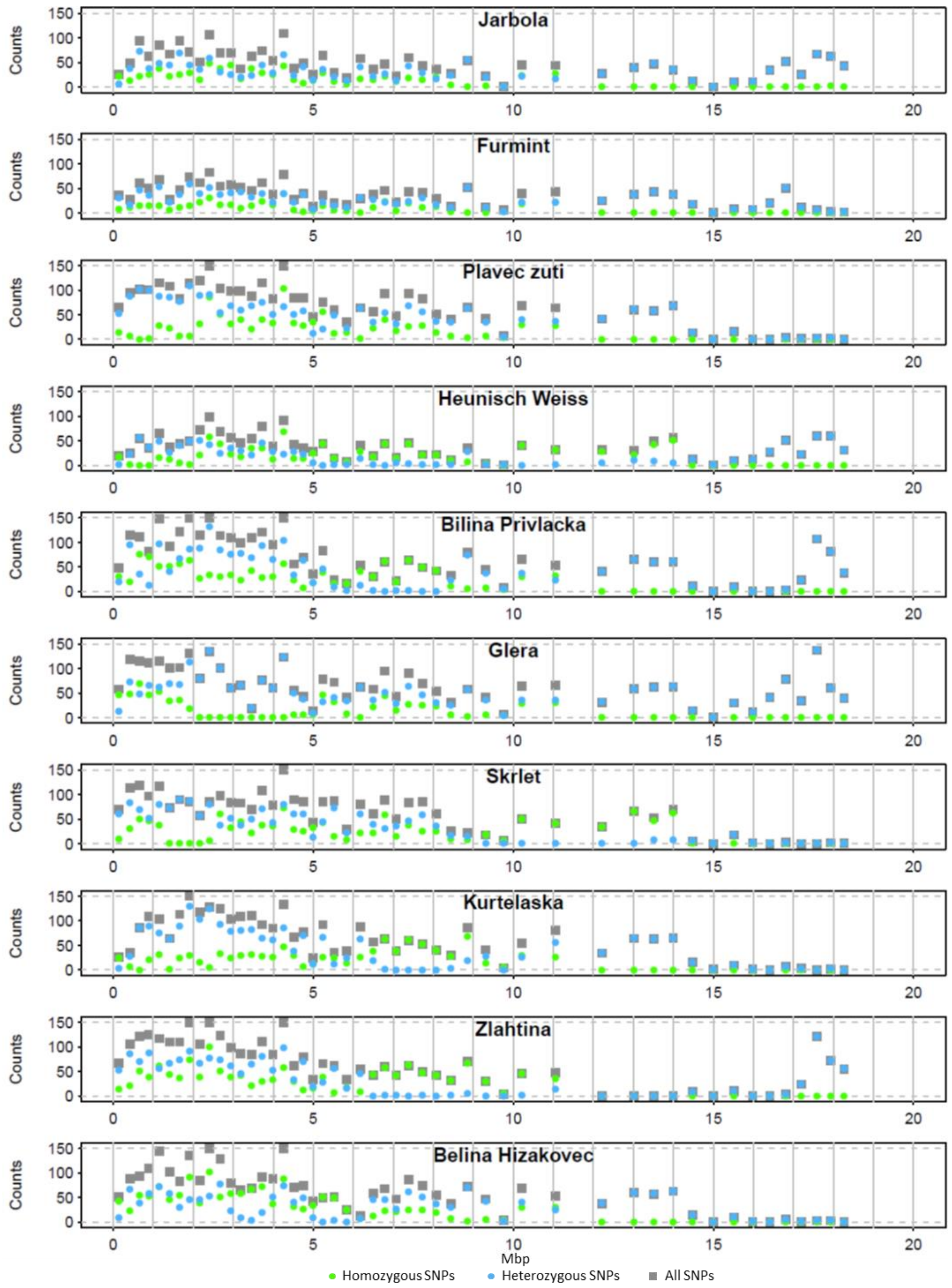

Fig. S4 (continued) Comparison between 65 cultivars of the present paper bearing yellow or green berries and the Chr2 homolog carrying the white *sativa* haplotype in the reference genome of *V. vinifera* PN40024 12Xv0 around the *MybA* array at position Chr2:14.1-14.3 Mbp. SNP density and zygosity in non-overlapping genomic windows containing 200 Kb of non-repetitive DNA. Along the y-axis, counts were plotted at a maximum value of 150 if higher.

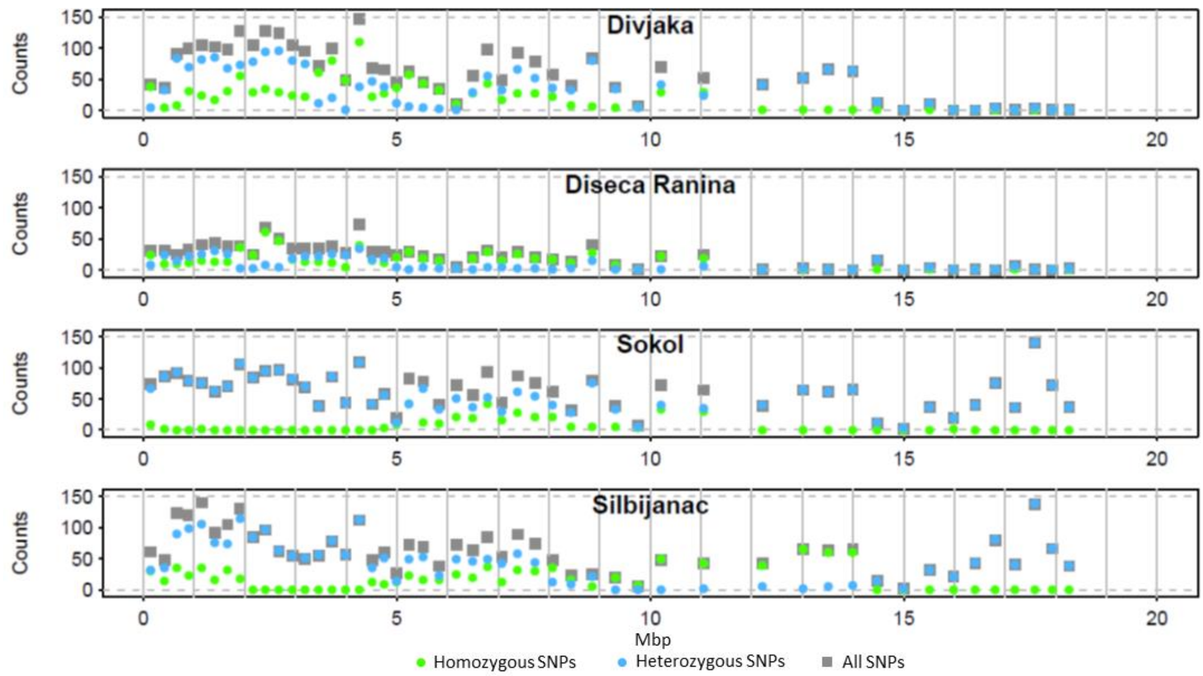

Fig. S4 (continued) Comparison between 65 cultivars of the present paper bearing yellow or green berries and the Chr2 homolog carrying the white *sativa* haplotype in the reference genome of *V. vinifera* PN40024 12Xv0 around the *MybA* array at position Chr2:14.1-14.3 Mbp. SNP density and zygosity in non-overlapping genomic windows containing 200 Kb of non-repetitive DNA. Along the y-axis, counts were plotted at a maximum value of 150 if higher.

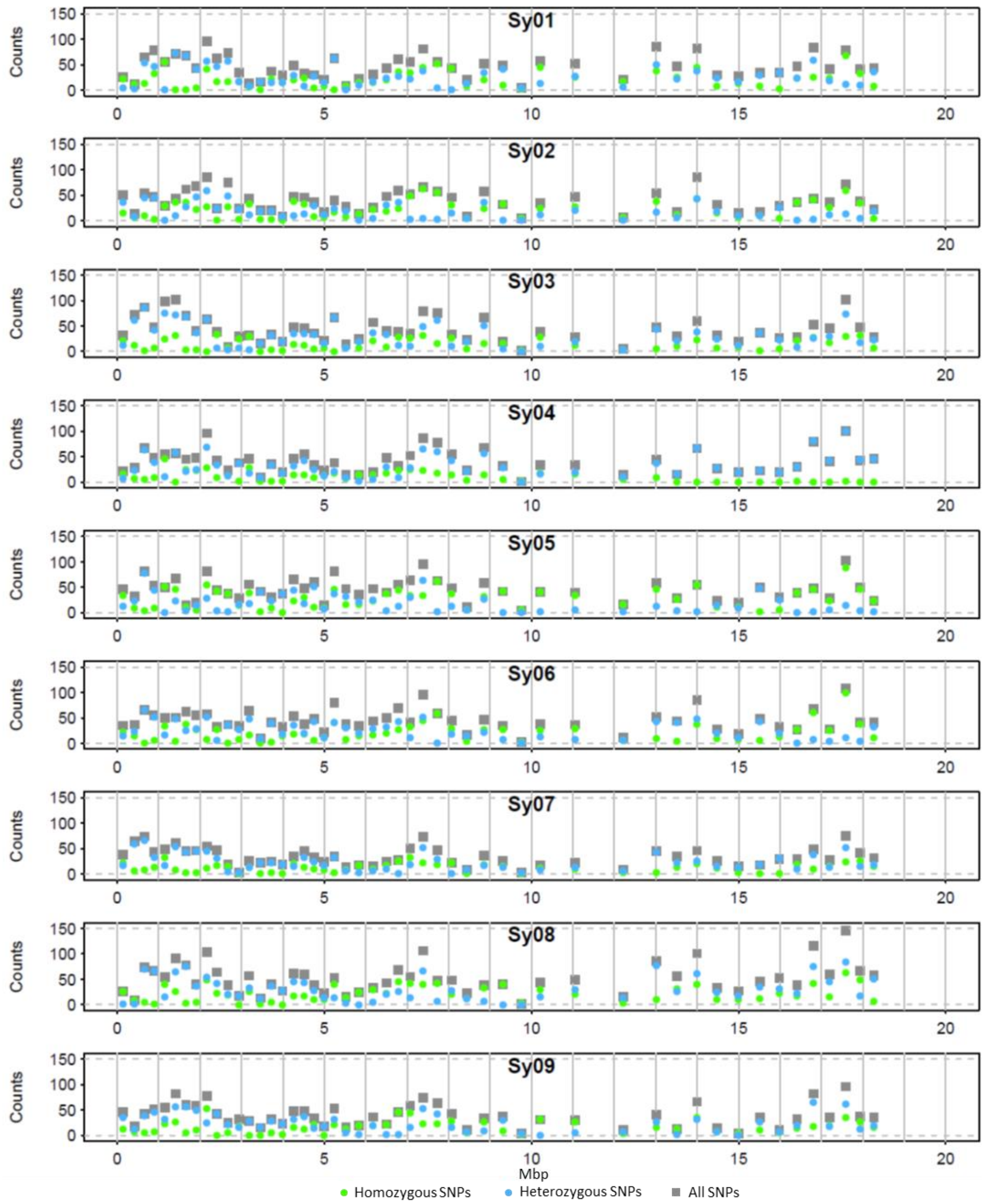

Fig. S5 Comparison between 9 spontaneous accessions at the natural site of Cerovica and the Chr2 homolog carrying the white *sativa* haplotype in the reference genome of *V. vinifera* PN40024 12Xv0 around the *MybA* array at position Chr2:14.1-14.3 Mbp. SNP density and zygosity in non-overlapping genomic windows containing 200 Kb of non-repetitive DNA. Along the y-axis, counts were plotted at a maximum value of 150 if higher. The accession Sy04 shows only heterozygous SNPs in the windows spanning the *MybA* array and the downstream region down to the lower end of Chr2, indicating that it carries one copy of the white *sativa* haplotype of PN40024 and this copy has been shared through a relatively recent common ancestor.

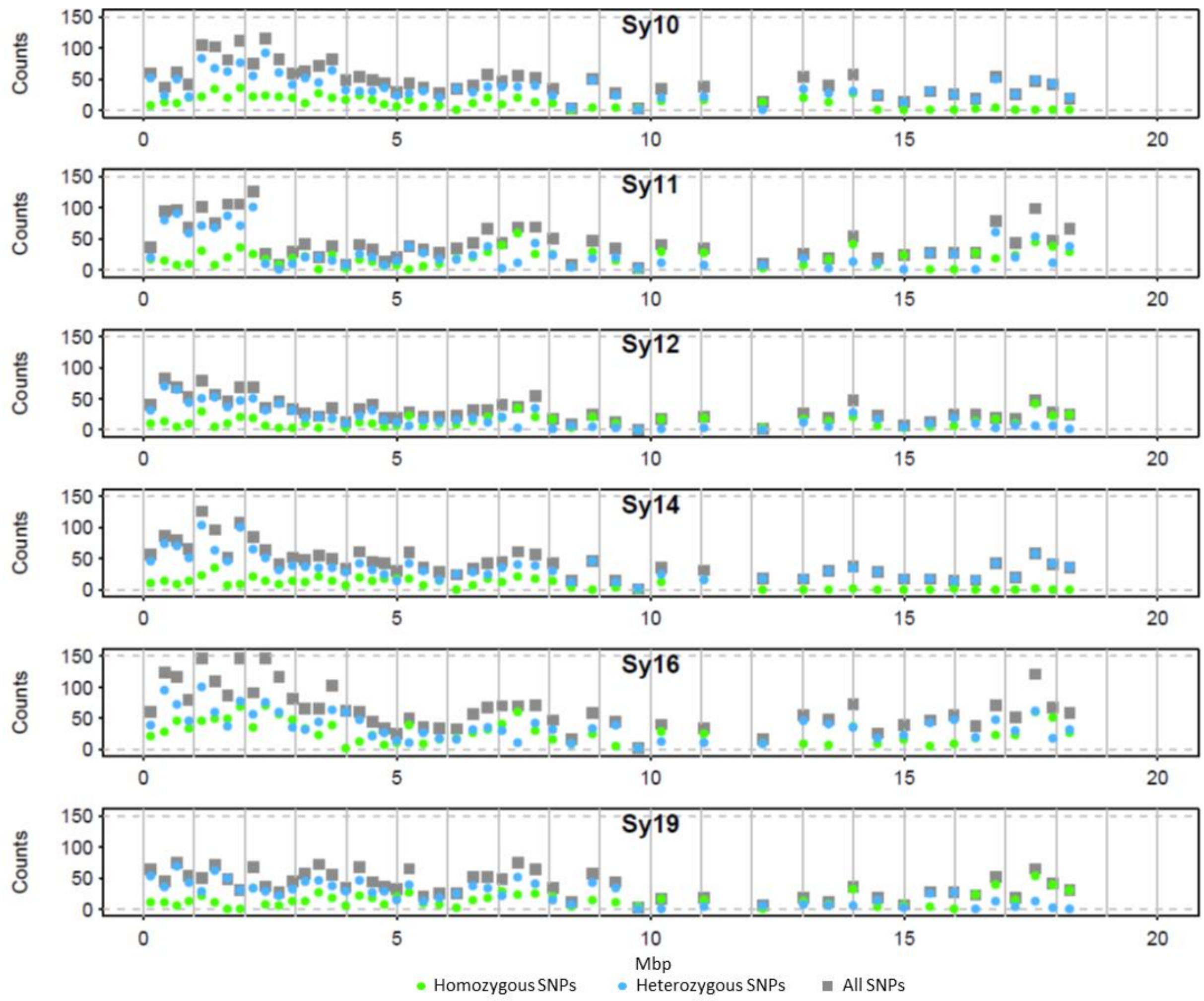

Fig. S6 Comparison between 6 spontaneous accessions at the natural site of Modro jezero and the Chr2 homolog carrying the white *sativa* haplotype in the reference genome of *V. vinifera* PN40024 12Xv0 around the *MybA* array at position Chr2:14.1-14.3 Mbp. SNP density and zygosity in non-overlapping genomic windows containing 200 Kb of non-repetitive DNA. Along the y-axis, counts were plotted at a maximum value of 150 if higher. The accessions Sy10 and Sy14 show only heterozygous SNPs in the windows spanning the *MybA* array and the downstream region down to the lower end of Chr2, indicating that they carry one copy of the white *sativa* haplotype of PN40024 and this copy has been shared through a relatively recent common ancestor.

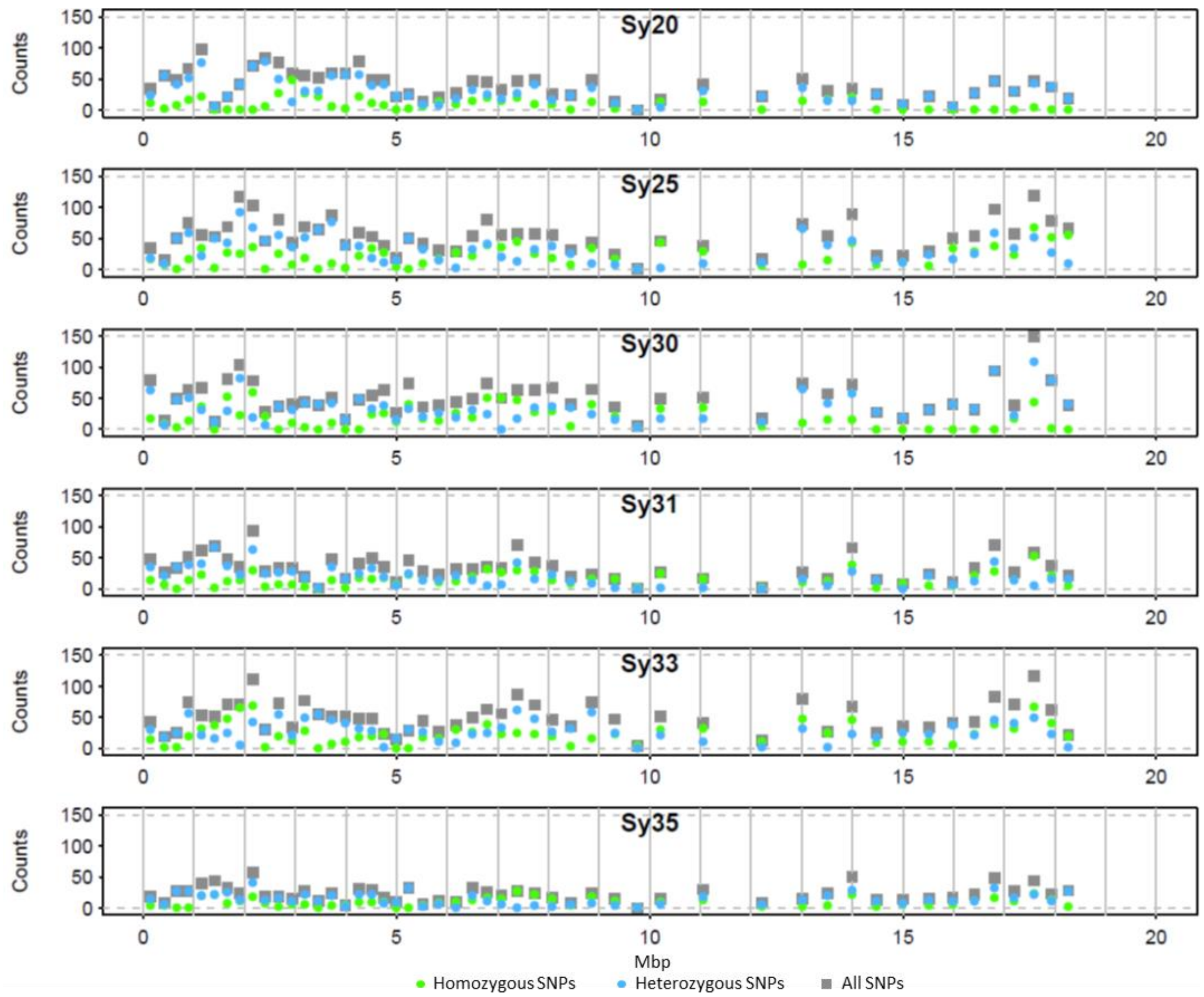

Fig. S7 Comparison between 6 spontaneous accessions at the natural site of Psunj and the Chr2 homolog carrying the white *sativa* haplotype in the reference genome of *V. vinifera* PN40024 12Xv0 around the *MybA* array at position Chr2:14.1-14.3 Mbp. SNP density and zygosity in non-overlapping genomic windows containing 200 Kb of non-repetitive DNA. Along the y-axis, counts were plotted at a maximum value of 150 if higher. The accessions Sy20 and Sy30 show only heterozygous SNPs in the windows spanning the *MybA* array and the downstream region, indicating that they carry one copy of the white *sativa* haplotype of PN40024 and this copy has been shared through a relatively recent common ancestor.

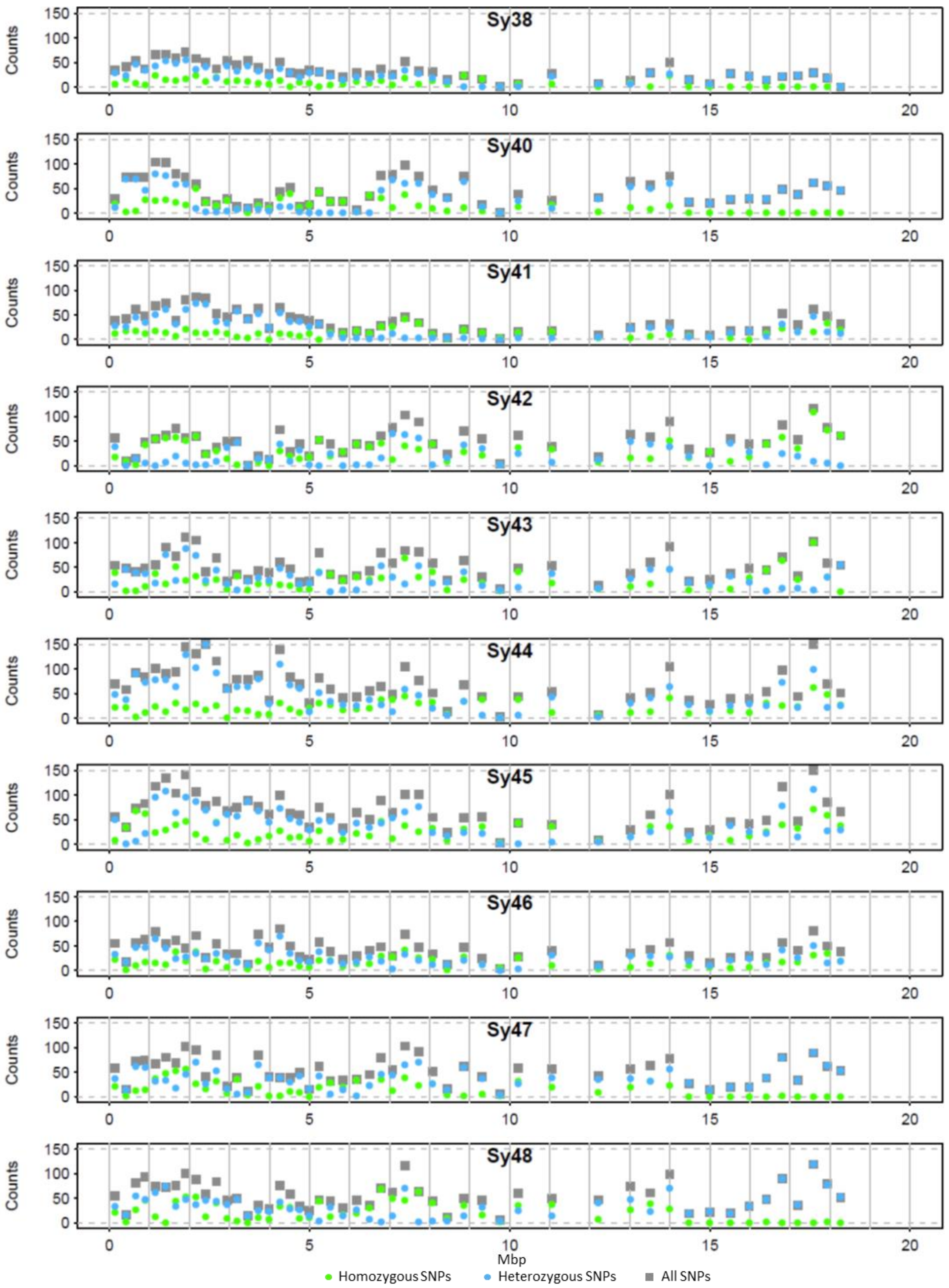

Fig. S8 Comparison between 20 spontaneous accessions at the natural site of Paklenika and the Chr2 homolog carrying the white *sativa* haplotype in the reference genome of *V. vinifera* PN40024 12Xv0 around the *MybA* array at position Chr2:14.1-14.3 Mbp. SNP density and zygosity in non-overlapping genomic windows containing 200 Kb of non-repetitive DNA. Along the y-axis, counts were plotted at a maximum value of 150 if higher. Multiple accessions show only heterozygous SNPs

in the windows spanning the *MybA* array and the downstream region, indicating that they carry one copy of the white *sativa* haplotype of PN40024 and this copy has been shared through a relatively recent common ancestor.

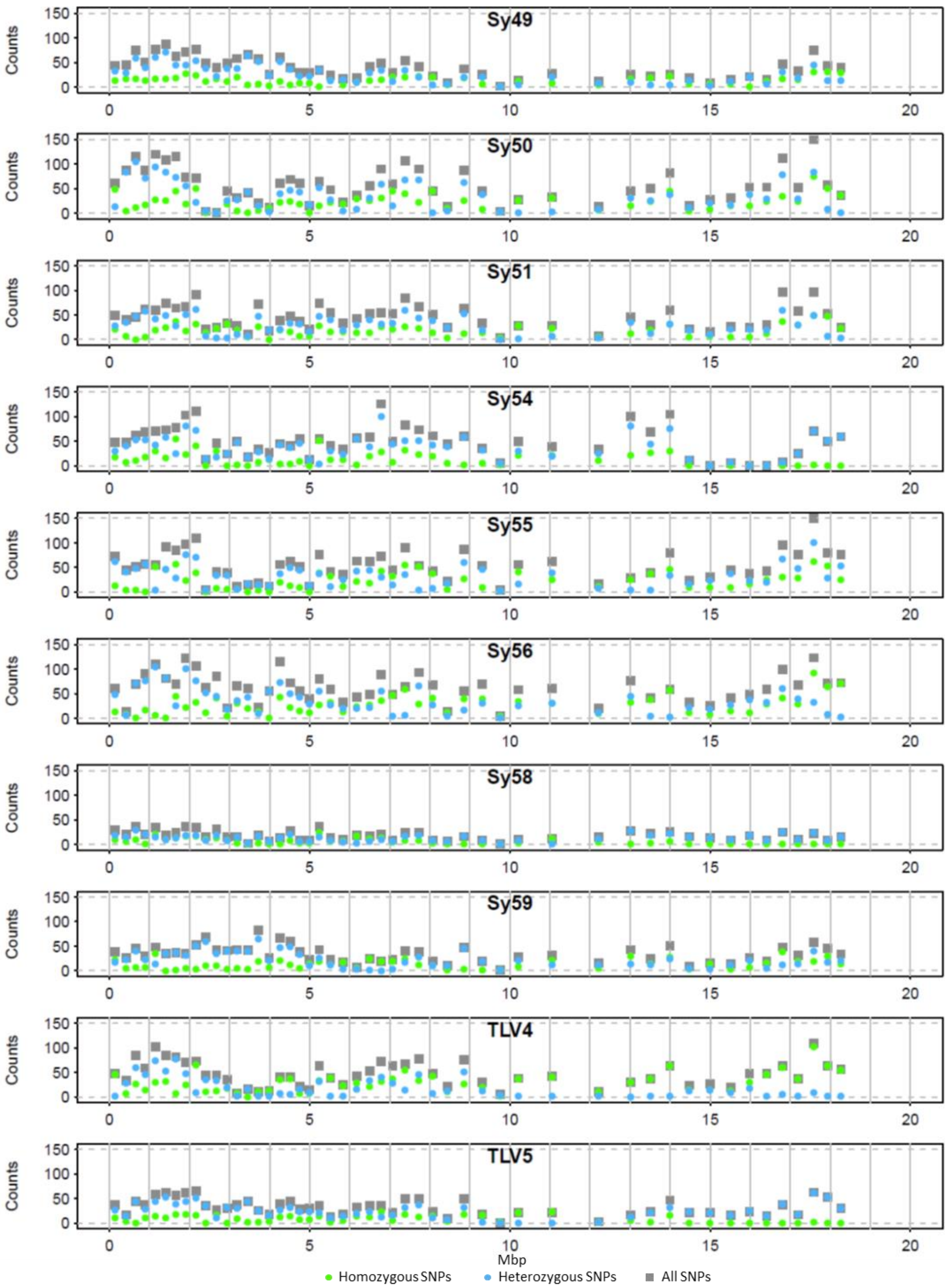

Fig. S8 (continued) Comparison between 20 spontaneous accessions at the natural site of Paklenika and the Chr2 homolog carrying the white *sativa* haplotype in the reference genome of *V. vinifera* PN40024 12Xv0 around the *MybA* array at position Chr2:14.1-14.3 Mbp. SNP density and zygosity in non-overlapping genomic windows containing 200 Kb of non-repetitive DNA. Along the y-axis, counts were plotted at a maximum value of 150 if higher. Multiple accessions show only heterozygous SNPs

in the windows spanning the *MybA* array and the downstream region, indicating that they carry one copy of the white *sativa* haplotype of PN40024 and this copy has been shared through a relatively recent common ancestor. The accession Sy54 shows no substantial SNPs the windows spanning the *MybA* array and the downstream region, a condition identical to white-berried cultivars, indicating that Sy54 carries two identical copies of the white *sativa* haplotype of PN40024 around the *MybA* array and one of these copy is identical to PN40024 down to the lower end of Chr2.

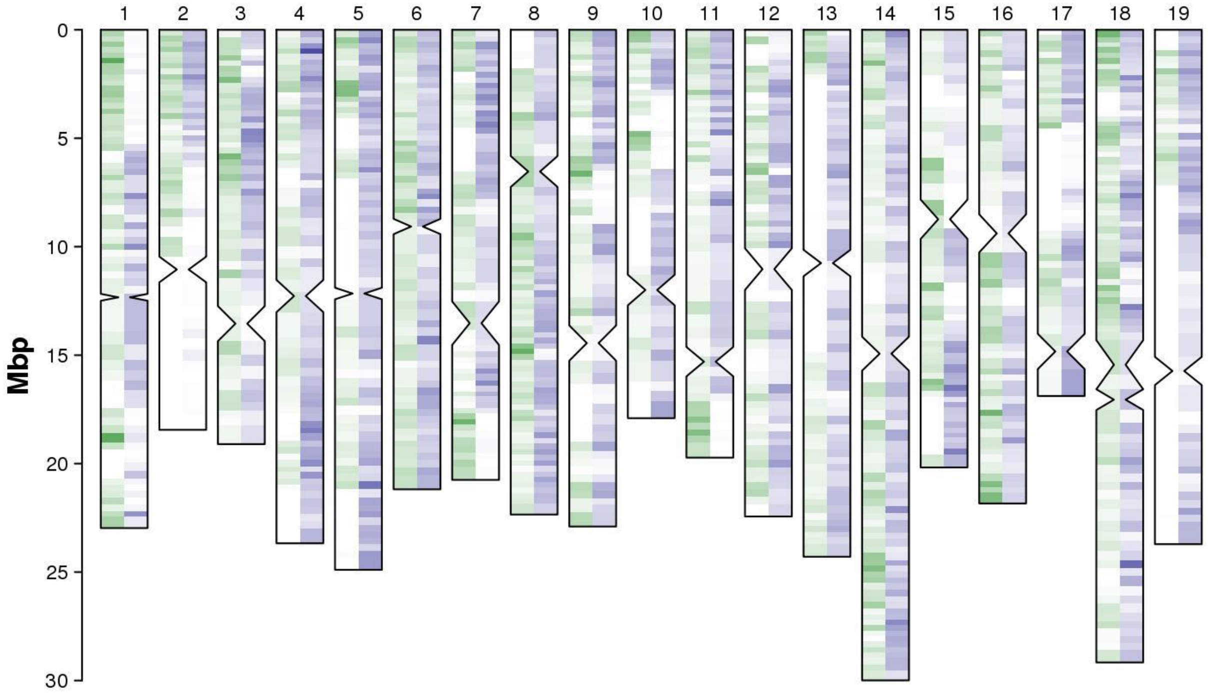

Fig. S9 Chromosomal distribution and density of SNPs in 'Bombino Bianco' compared to the reference genome assembly of *V. vinifera* PN40024 12Xv0. Vertical ideograms represent chromosomes. Constrictions indicate the location of centromeric repeats. Densities of heterozygous SNP (white-to-blue heatmap shown in the right-hand portion of each chromosome, max blue = 235 SNPs) and homozygous (white-to-green heatmap shown in the left-hand portion of each chromosome, max green = 162 SNPs) are calculated in non-overlapping 200-Kb windows.

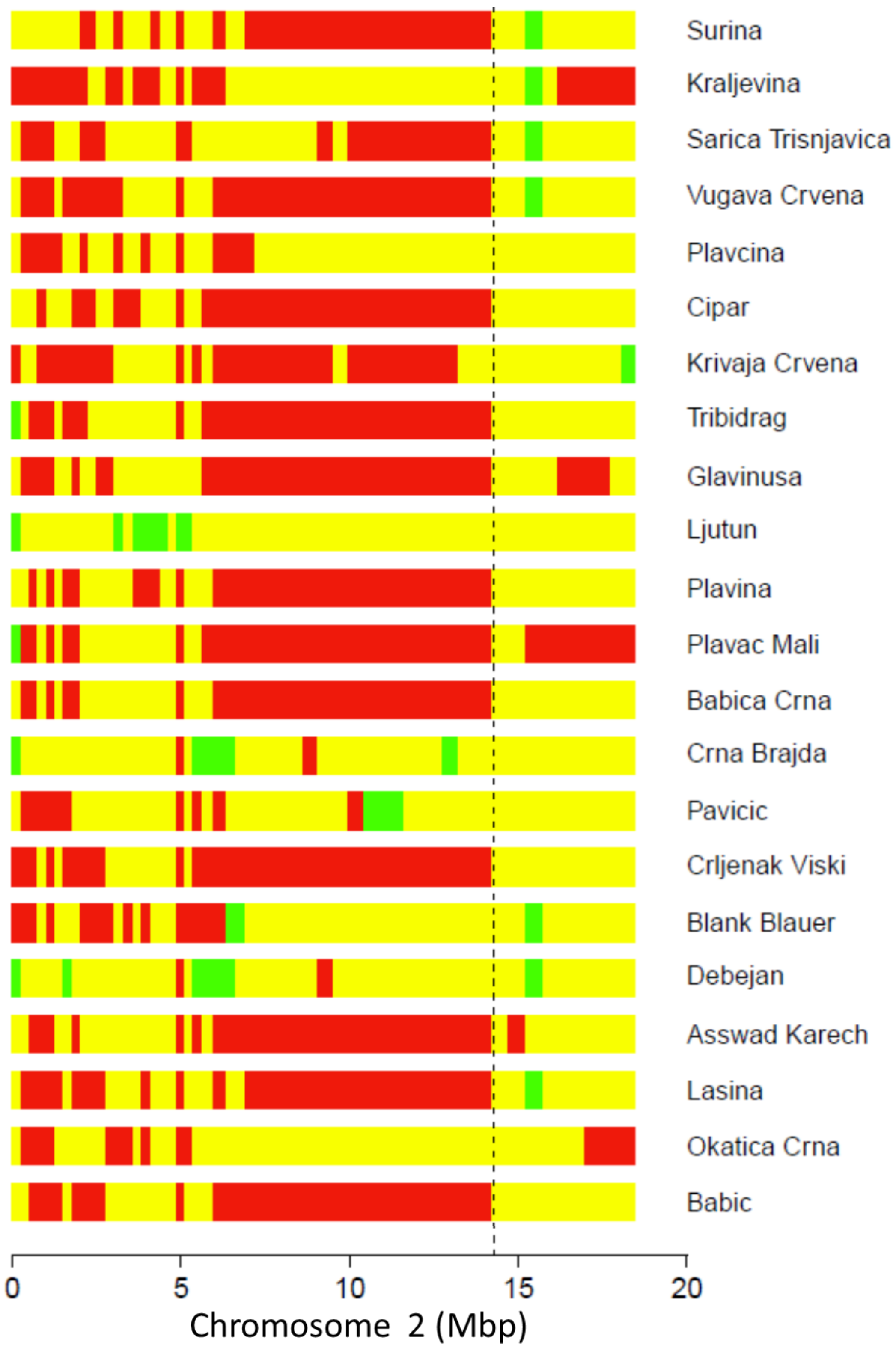

Fig. S10 Identity by descent (IBD) between pink or red or blue/black cultivars and the Chr2 homolog in 'Bombino Bianco', containing the inactivating mutations in the MybA array. Red indicates genomic windows with IBD=0 (no shared haplotype). Yellow indicates genomic windows with IBD=1 (one shared haplotype). Green indicates genomic windows with IBD=2 (two shared haplotypes). Each genomic window includes 200 Kb of non-repetitive DNA. The dashed line indicates the location of the MybA array (Chr2:14.1-14.3 Mbp). The nearest genomic windows have coordinates Chr2:13,775,305-14,215,737 and Chr2:14,215,738-14,735,623.

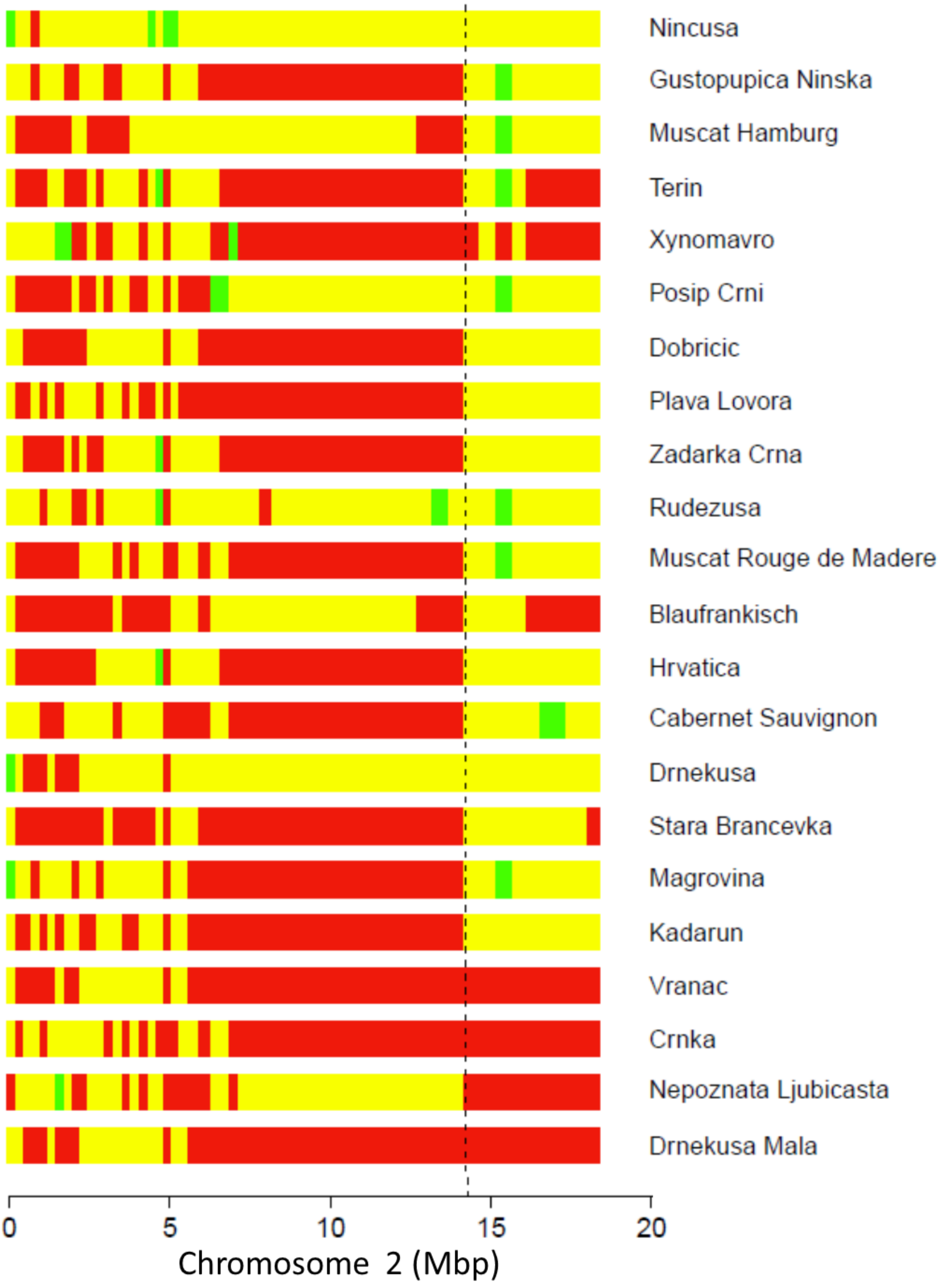

Fig. S10 (continued) Identity by descent (IBD) between pink or red or blue/black cultivars and the Chr2 homolog in 'Bombino Bianco', containing the inactivating mutations in the MybA array. Red indicates genomic windows with IBD=0 (no shared haplotype). Yellow indicates genomic windows with IBD=1 (one shared haplotype). Green indicates genomic windows with IBD=2 (two shared haplotypes). Each genomic window includes 200 Kb of non-repetitive DNA. The dashed line indicates the location of the MybA array (Chr2:14.1-14.3 Mbp). The nearest genomic windows have coordinates Chr2:13,775,305-14,215,737 and Chr2:14,215,738-14,735,623.

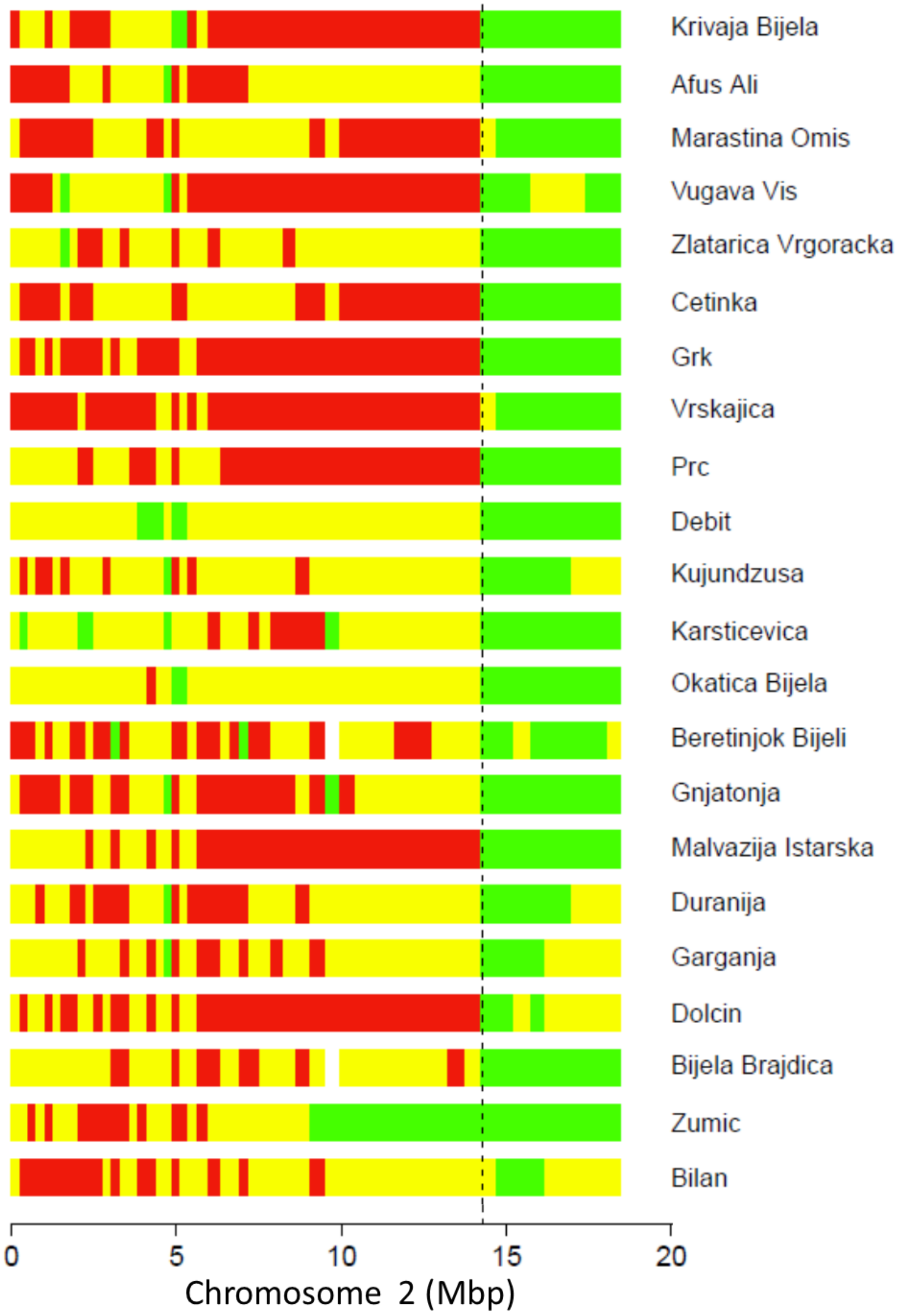

Fig. S11 Identity by descent (IBD) between green or yellow cultivars and the Chr2 homolog in 'Bombino Bianco', containing the inactivating mutations in the MybA array. Red indicates genomic windows with IBD=0 (no shared haplotype). Yellow indicates genomic windows with IBD=1 (one shared haplotype). Green indicates genomic windows with IBD=2 (two shared haplotypes). Each genomic window includes 200 Kb of non-repetitive DNA. The dashed line indicates the location of the MybA array (Chr2:14.1-14.3 Mbp). The nearest genomic windows have coordinates Chr2:13,775,305-14,215,737 and Chr2:14,215,738-14,735,623.

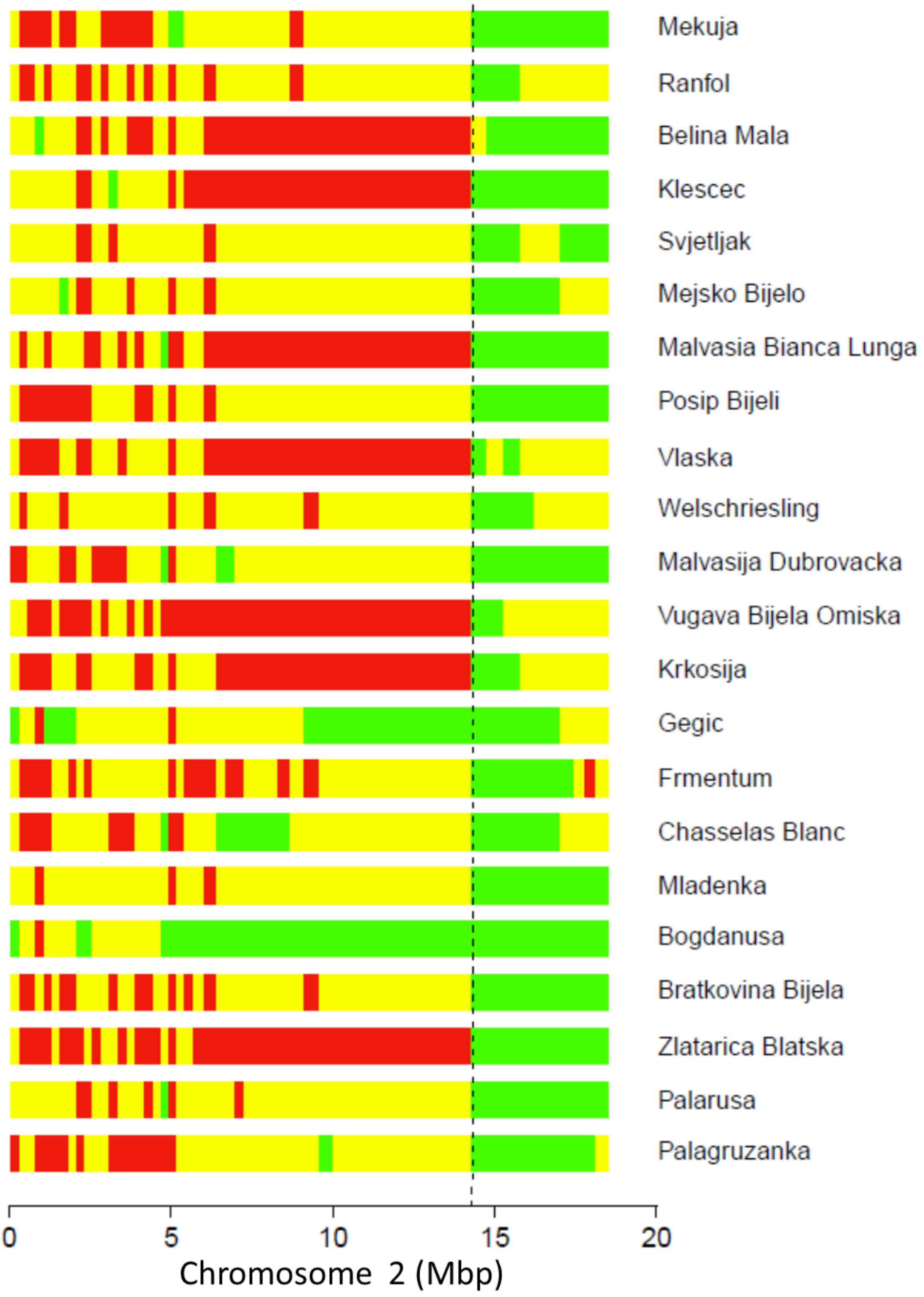

Fig. S11 (continued) Identity by descent (IBD) between green or yellow cultivars and the Chr2 homolog in 'Bombino Bianco', containing the inactivating mutations in the MybA array. Red indicates genomic windows with IBD=0 (no shared haplotype). Yellow indicates genomic windows with IBD=1 (one shared haplotype). Green indicates genomic windows with IBD=2 (two shared haplotypes). Each genomic window includes 200 Kb of non-repetitive DNA. The dashed line indicates the location of the MybA array (Chr2:14.1-14.3 Mbp). The nearest genomic windows have coordinates Chr2:13,775,305-14,215,737 and Chr2:14,215,738-14,735,623.

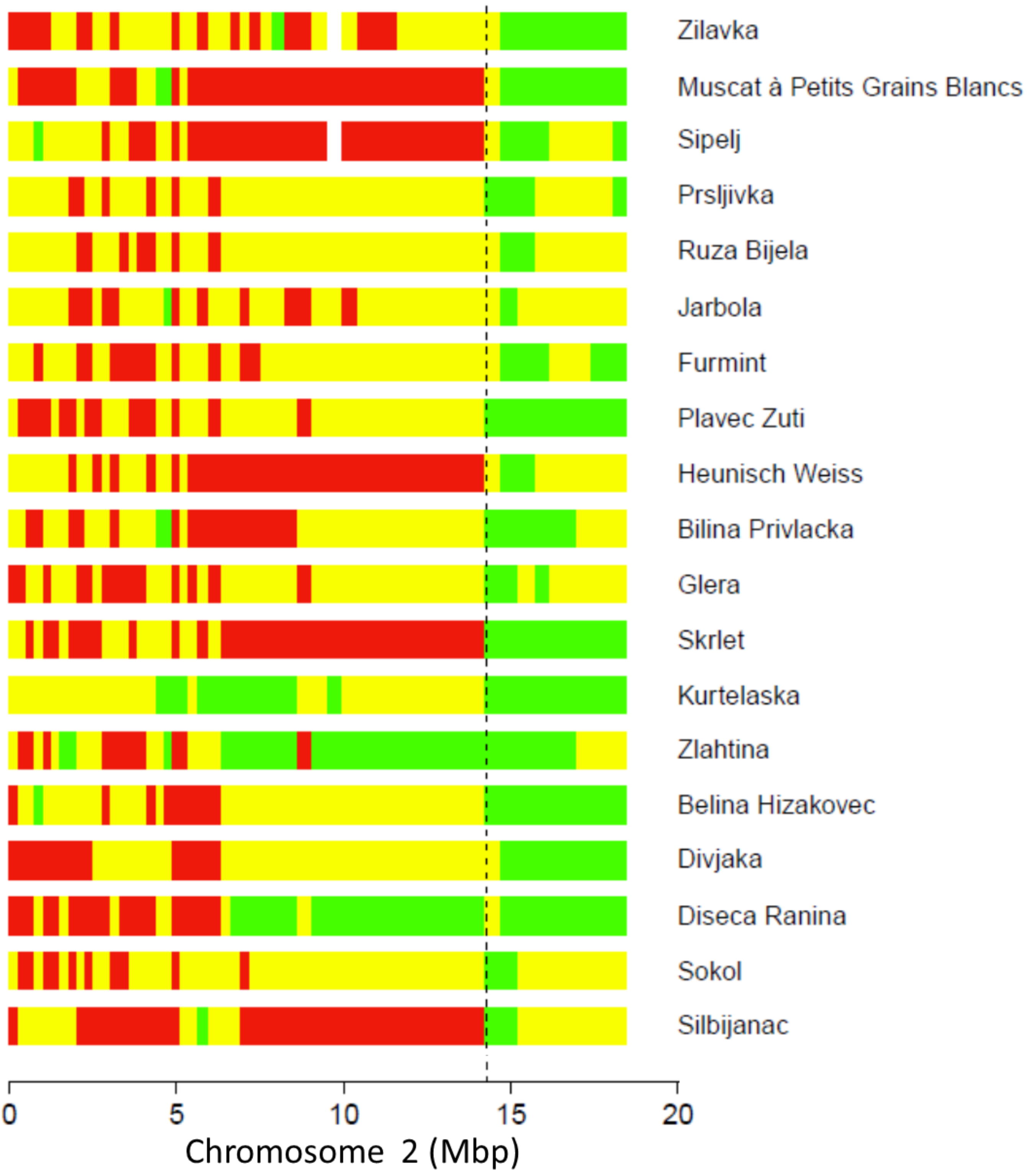

Fig. S11 (continued) Identity by descent (IBD) between green or yellow cultivars and the Chr2 homolog in 'Bombino Bianco', containing the inactivating mutations in the MybA array. Red indicates genomic windows with IBD=0 (no shared haplotype). Yellow indicates genomic windows with IBD=1 (one shared haplotype). Green indicates genomic windows with IBD=2 (two shared haplotypes). Each genomic window includes 200 Kb of non-repetitive DNA. The dashed line indicates the location of the MybA array (Chr2:14.1-14.3 Mbp). The nearest genomic windows have coordinates Chr2:13,775,305-14,215,737 and Chr2:14,215,738-14,735,623.

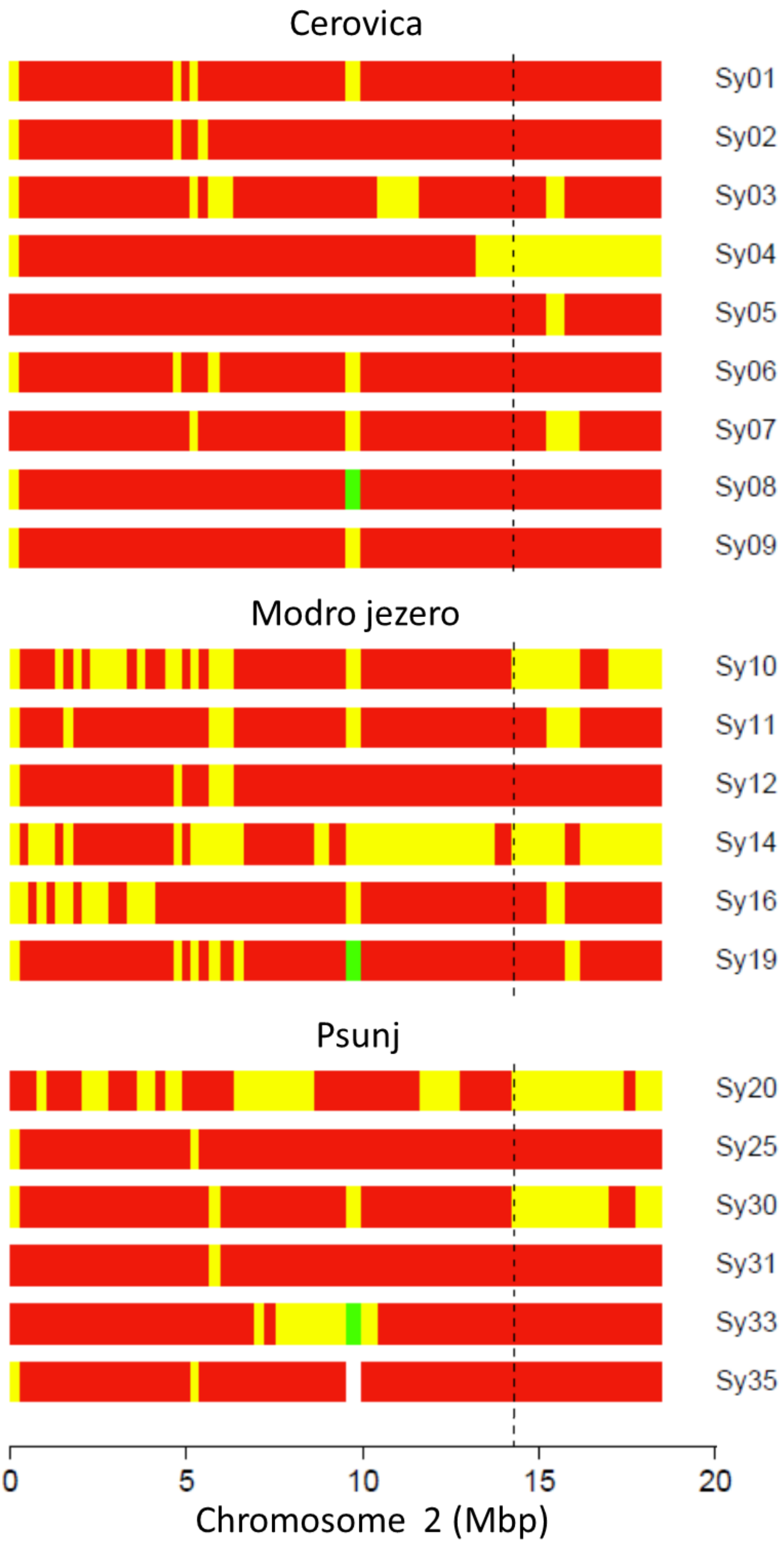

Fig. S12 Identity by descent (IBD) spontaneous individuals at natural sites and the Chr2 homolog in 'Bombino Bianco', containing the inactivating mutations in the MybA array. Red indicates genomic windows with IBD=0 (no shared haplotype). Yellow indicates genomic windows with IBD=1 (one shared haplotype). Green indicates genomic windows with IBD=2 (two shared haplotypes). Each genomic window includes 200 Kb of non-repetitive DNA. The dashed line indicates the location of the MybA array (Chr2:14.1-14.3 Mbp). The nearest genomic windows have coordinates Chr2:13,775,305-14,215,737 and Chr2:14,215,738-14,735,623.

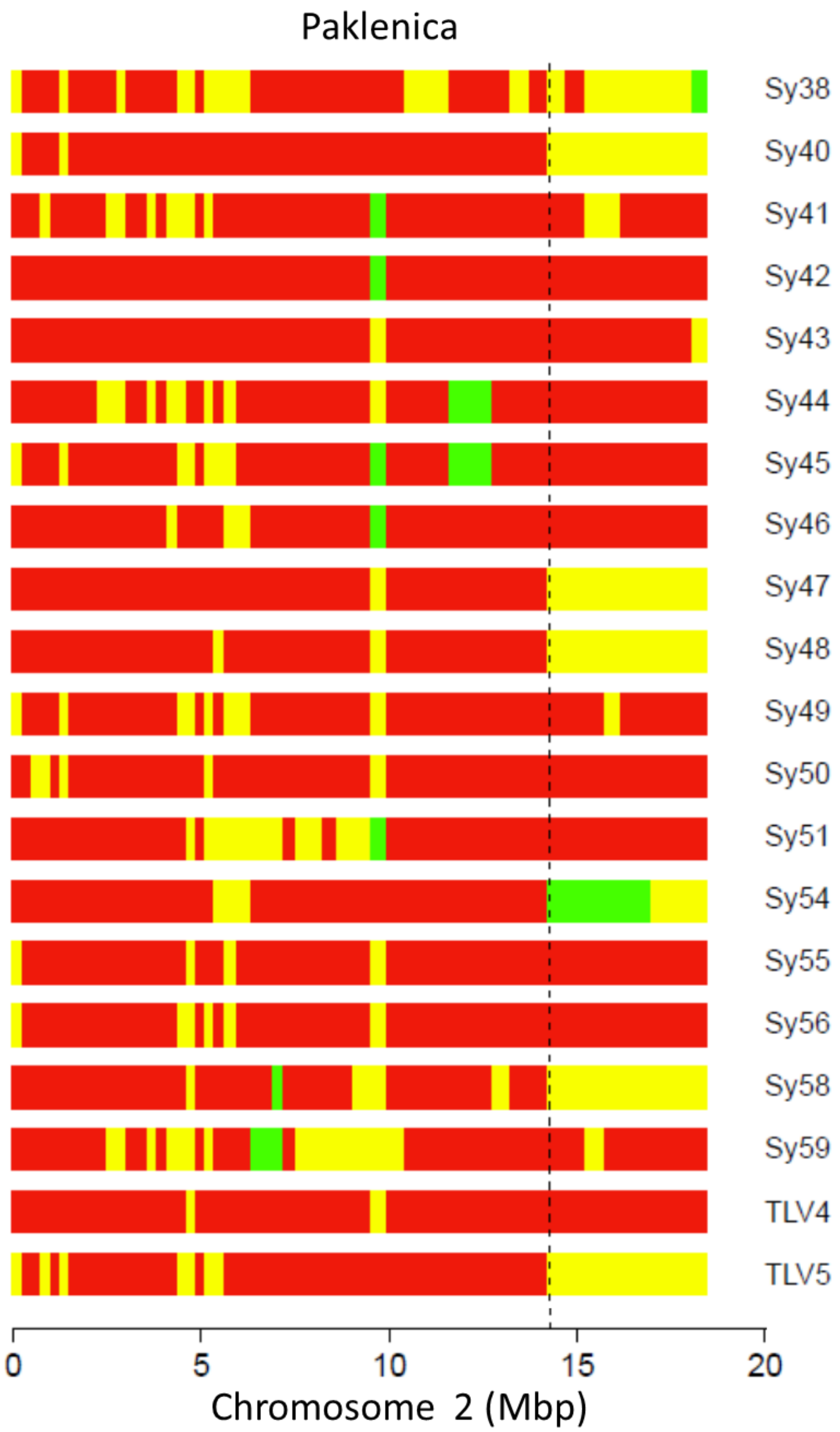

Fig. S12 (continued) Identity by descent (IBD) spontaneous individuals at natural sites and the Chr2 homolog in 'Bombino Bianco', containing the inactivating mutations in the MybA array. Red indicates genomic windows with IBD=0 (no shared haplotype). Yellow indicates genomic windows with IBD=1 (one shared haplotype). Green indicates genomic windows with IBD=2 (two shared haplotypes). Each genomic window includes 200 Kb of non-repetitive DNA. The dashed line indicates the location of the MybA array (Chr2:14.1-14.3 Mbp). The nearest genomic windows have coordinates Chr2:13,775,305-14,215,737 and Chr2:14,215,738-14,735,623.

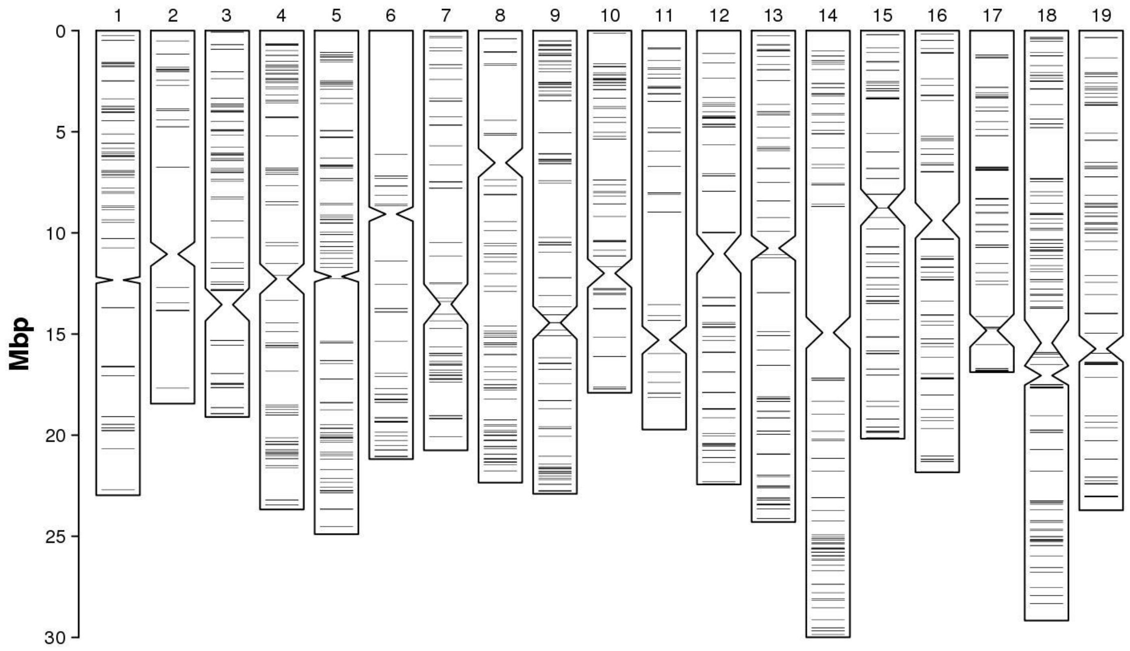

Fig. S13 Random distribution of unmatching SNPs (black ticks) across the genome for the 'Surina' pedigree (Heunisch Weiss × Blank Blauer). Vertical ideograms represent chromosomes of the reference genome assembly of *V. vinifera* PN40024 12Xv0. Chromosome size is indicated on the y-axis in million base pairs (Mbp). Constrictions indicate the location of centromeric repeats.

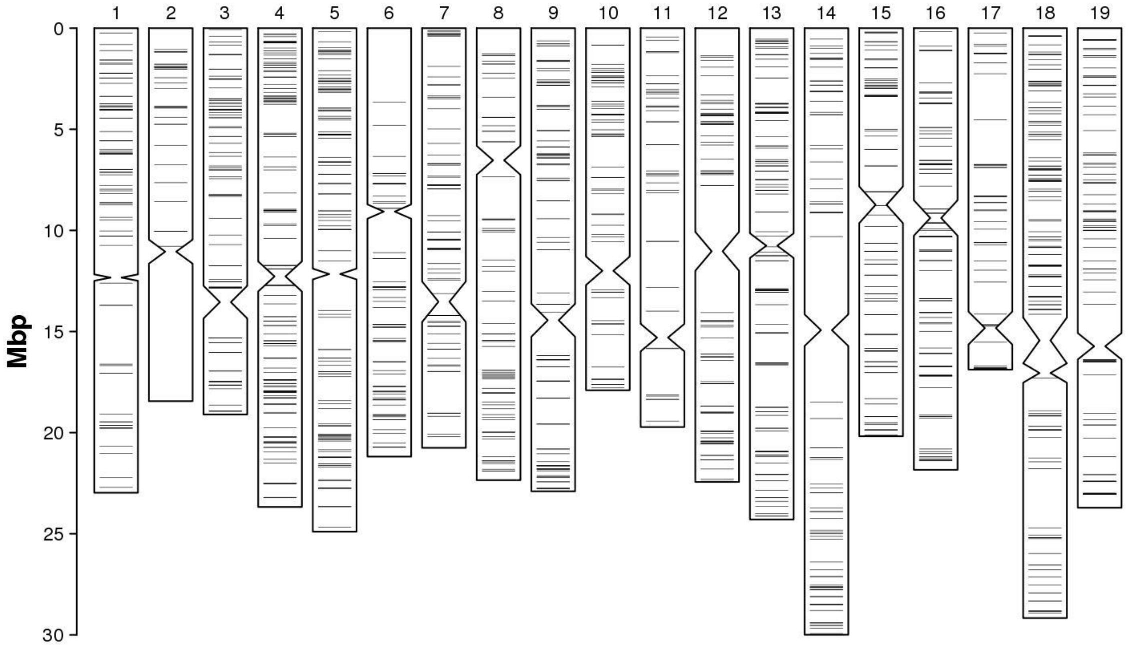

Fig. S14 Random distribution of unmatching SNPs (black ticks) across the genome for the 'Svjetljak' pedigree (Heunisch Weiss × Blank Blauer). Vertical ideograms represent chromosomes of the reference genome assembly of *V. vinifera* PN40024 12Xv0. Chromosome size is indicated on the y-axis in million base pairs (Mbp). Constrictions indicate the location of centromeric repeats.

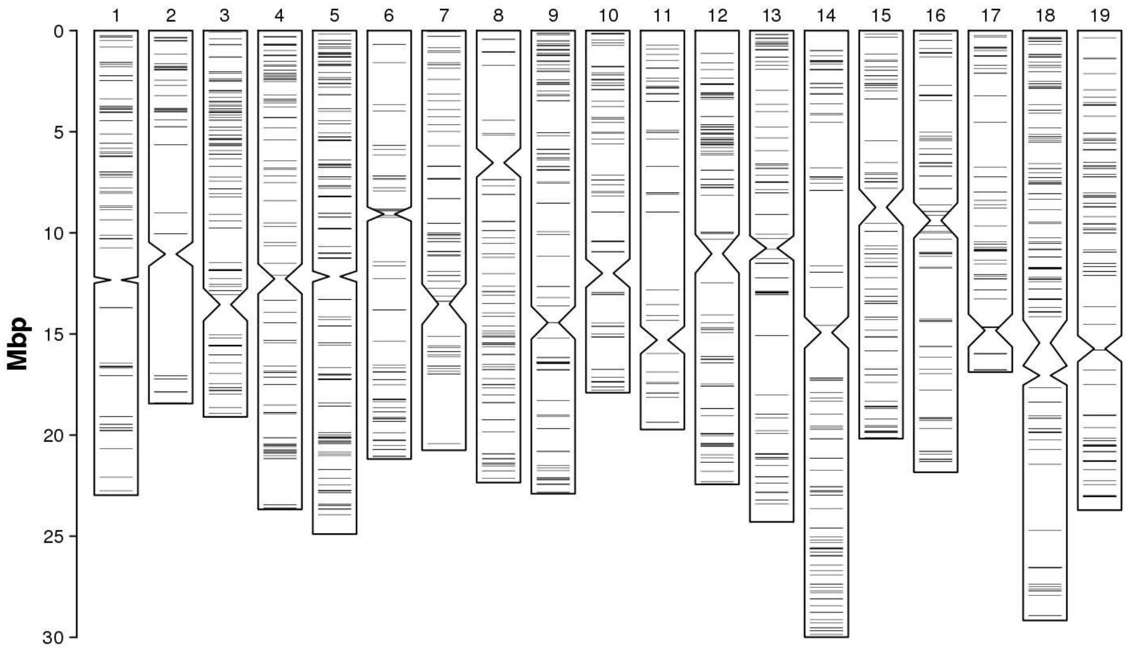

Fig. S15 Random distribution of unmatching SNPs (black ticks) across the genome for the 'Ranfol' pedigree (Heunisch Weiss × Blank Blauer). Vertical ideograms represent chromosomes of the reference genome assembly of *V. vinifera* PN40024 12Xv0. Chromosome size is indicated on the y-axis in million base pairs (Mbp). Constrictions indicate the location of centromeric repeats.

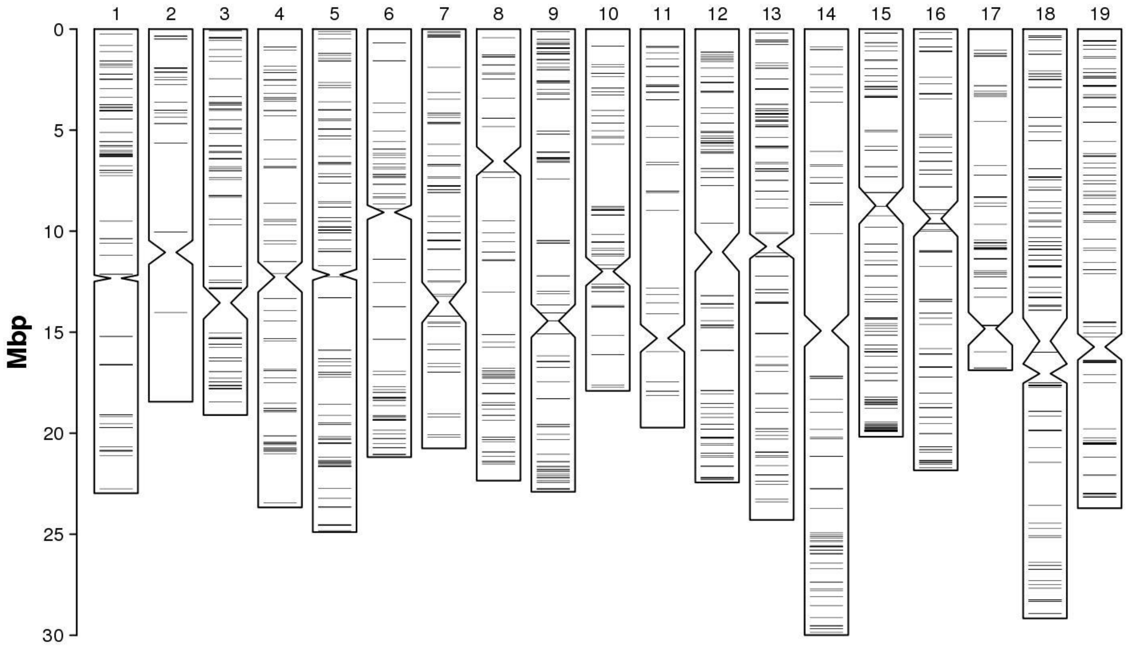

Fig. S16 Random distribution of unmatching SNPs (black ticks) across the genome for the 'Plavec žuti' pedigree (Heunisch Weiss × Blank Blauer). Vertical ideograms represent chromosomes of the reference genome assembly of *V. vinifera* PN40024 12Xv0. Chromosome size is indicated on the y-axis in million base pairs (Mbp). Constrictions indicate the location of centromeric repeats.

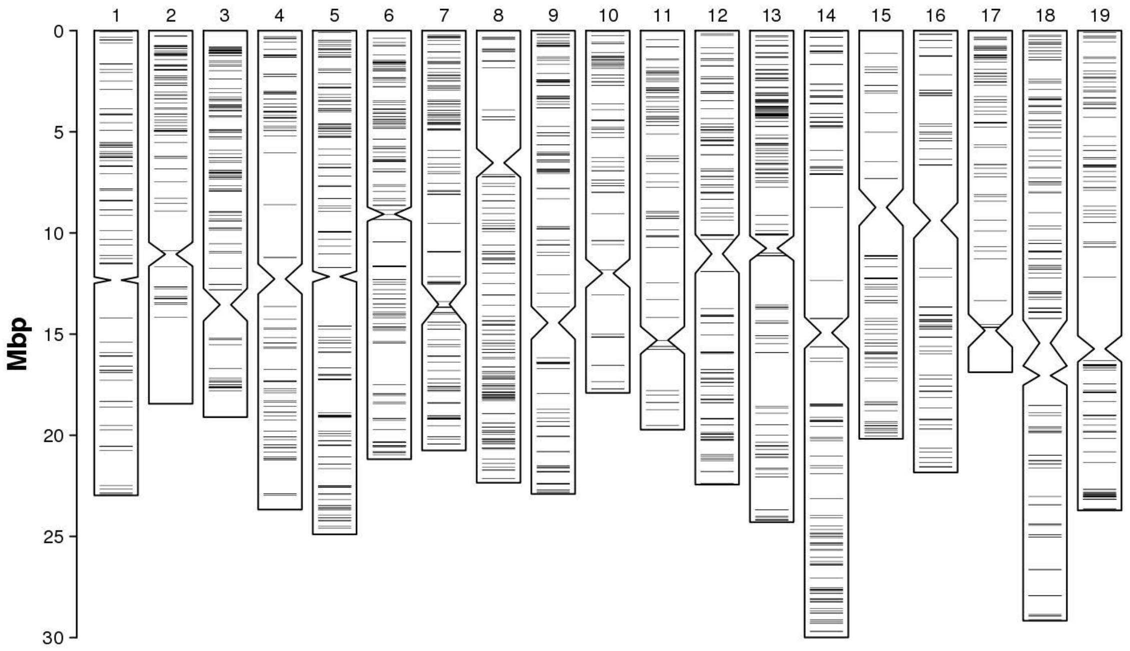

Fig. S17 Random distribution of unmatching SNPs (black ticks) across the genome for the 'Debit' pedigree (Bombino Bianco × Lasina). Vertical ideograms represent chromosomes of the reference genome assembly of *V. vinifera* PN40024 12Xv0. Chromosome size is indicated on the y-axis in million base pairs (Mbp). Constrictions indicate the location of centromeric repeats.

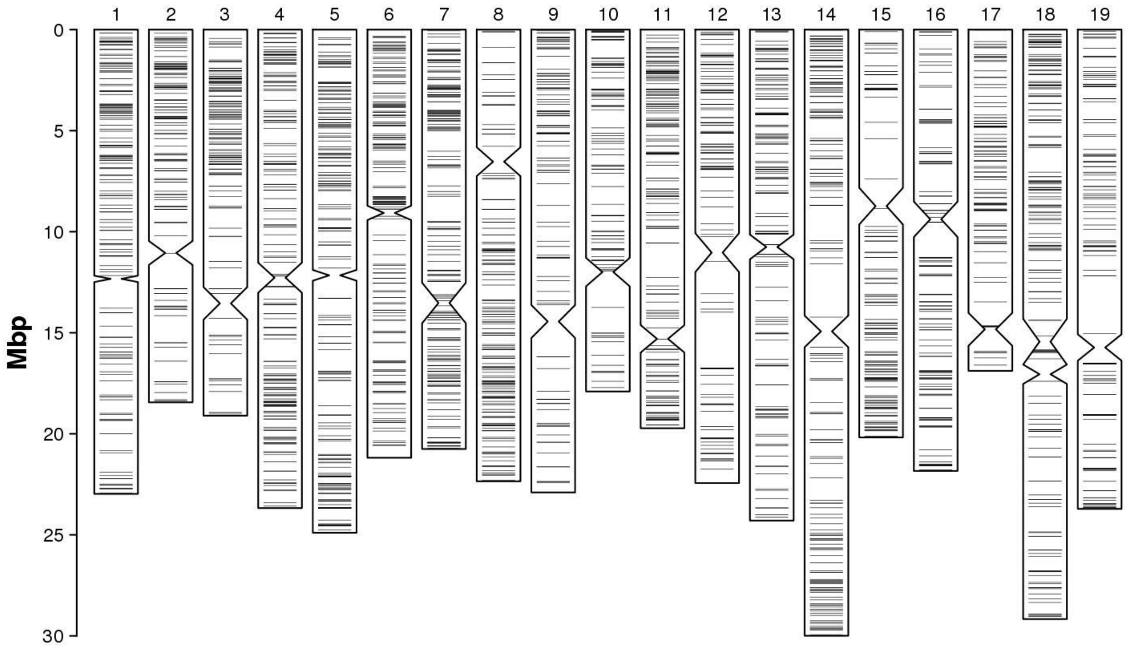

Fig. S18 Random distribution of unmatching SNPs (black ticks) across the genome for the 'Karstičevica' pedigree (Bombino Bianco × Plavina). Vertical ideograms represent chromosomes of the reference genome assembly of *V. vinifera* PN40024 12Xv0. Chromosome size is indicated on the y-axis in million base pairs (Mbp). Constrictions indicate the location of centromeric repeats.

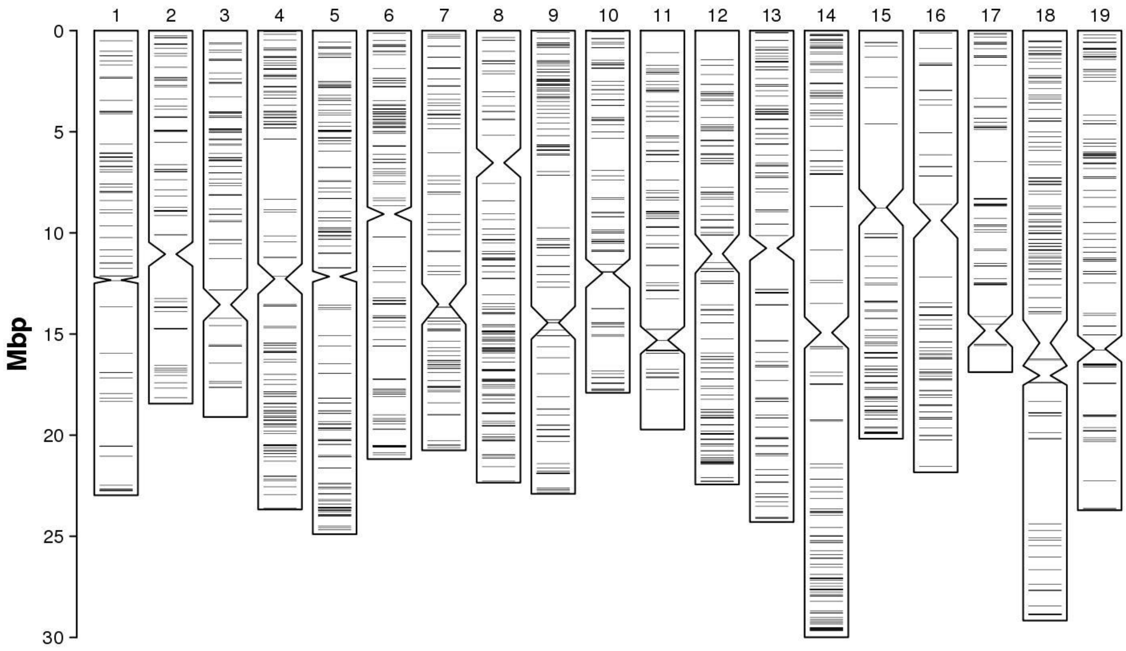

Fig. S19 Random distribution of unmatching SNPs (black ticks) across the genome for the 'Ljutun' pedigree (Bombino Bianco × Plavac Mali). Vertical ideograms represent chromosomes of the reference genome assembly of *V. vinifera* PN40024 12Xv0. Chromosome size is indicated on the y-axis in million base pairs (Mbp). Constrictions indicate the location of centromeric repeats.

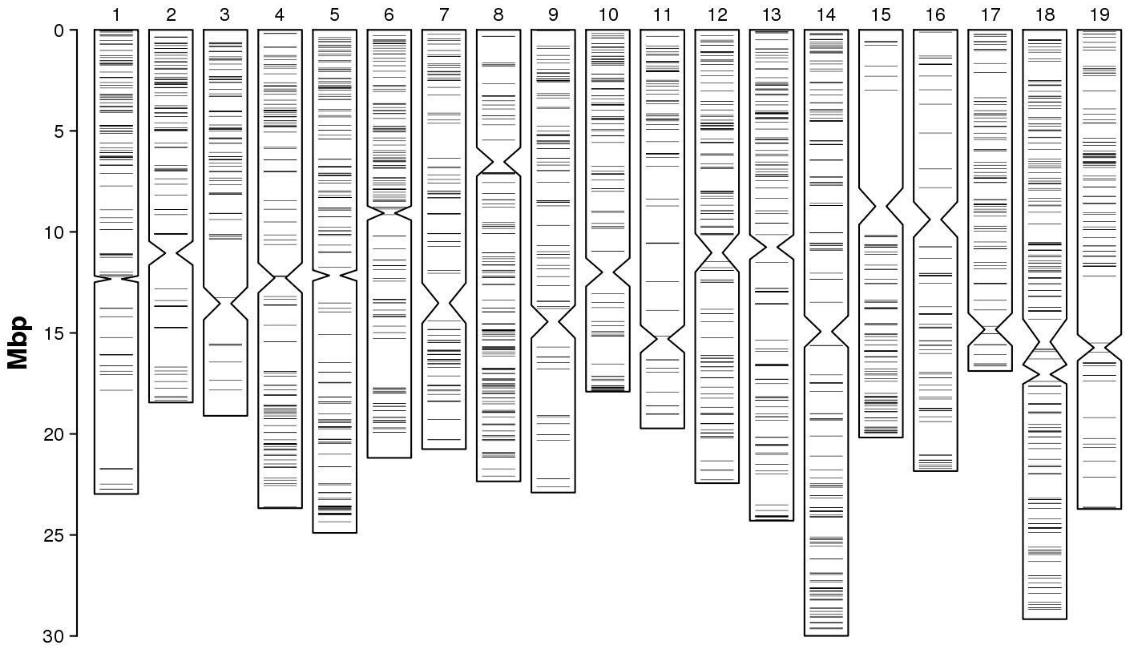

Fig. S20 Random distribution of unmatching SNPs (black ticks) across the genome for the 'Ninčuša' pedigree (Bombino Bianco × Plavac Mali). Vertical ideograms represent chromosomes of the reference genome assembly of *V. vinifera* PN40024 12Xv0. Chromosome size is indicated on the y-axis in million base pairs (Mbp). Constrictions indicate the location of centromeric repeats.

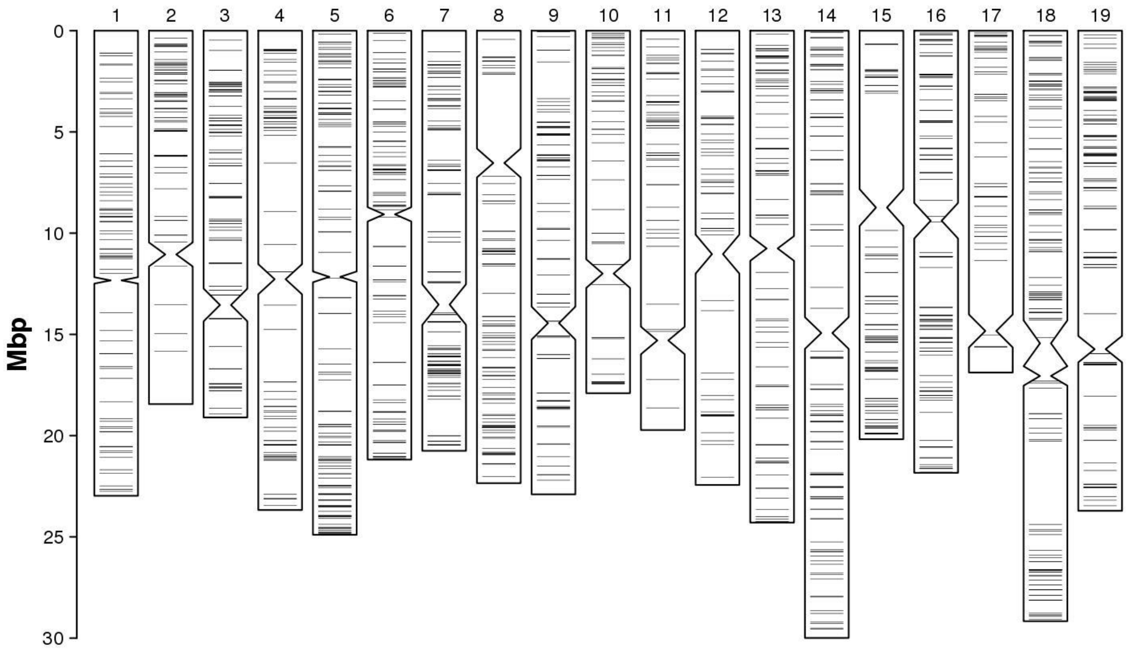

Fig. S21 Random distribution of unmatching SNPs (black ticks) across the genome for the 'Kurtelaška' pedigree (Bombino Bianco × Maraština Omiš). Vertical ideograms represent chromosomes of the reference genome assembly of *V. vinifera* PN40024 12Xv0. Chromosome size is indicated on the y-axis in million base pairs (Mbp). Constrictions indicate the location of centromeric repeats.

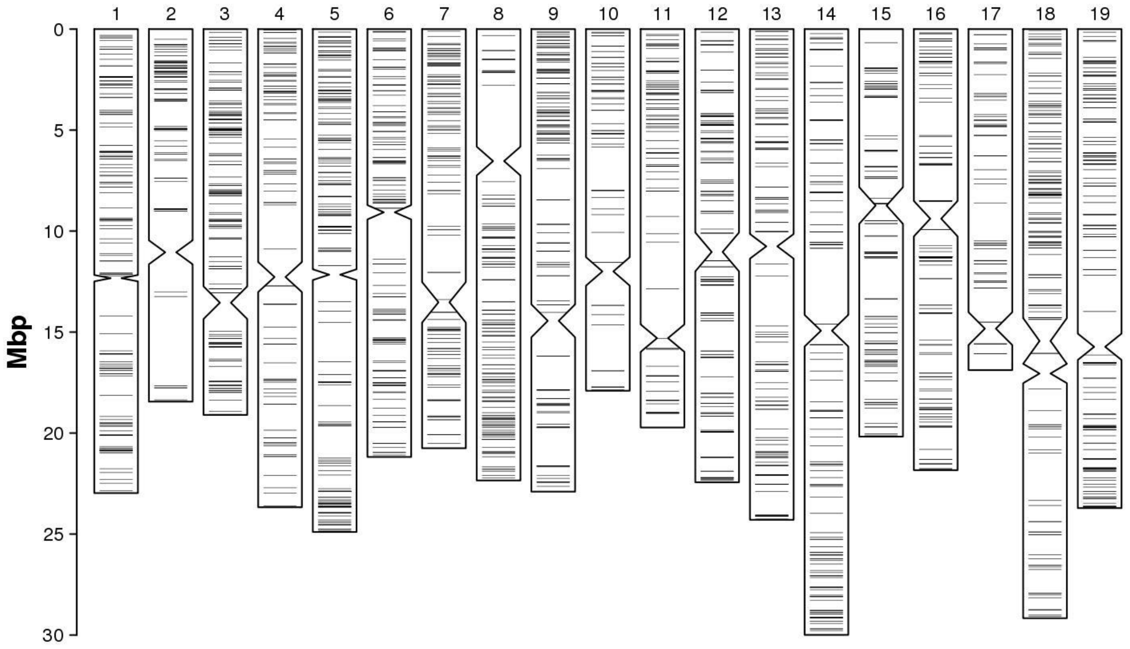

Fig. S22 Random distribution of unmatching SNPs (black ticks) across the genome for the 'Gegić' pedigree (Bombino Bianco × Bilina Privlačka). Vertical ideograms represent chromosomes of the reference genome assembly of *V. vinifera* PN40024 12Xv0. Chromosome size is indicated on the y-axis in million base pairs (Mbp). Constrictions indicate the location of centromeric repeats.

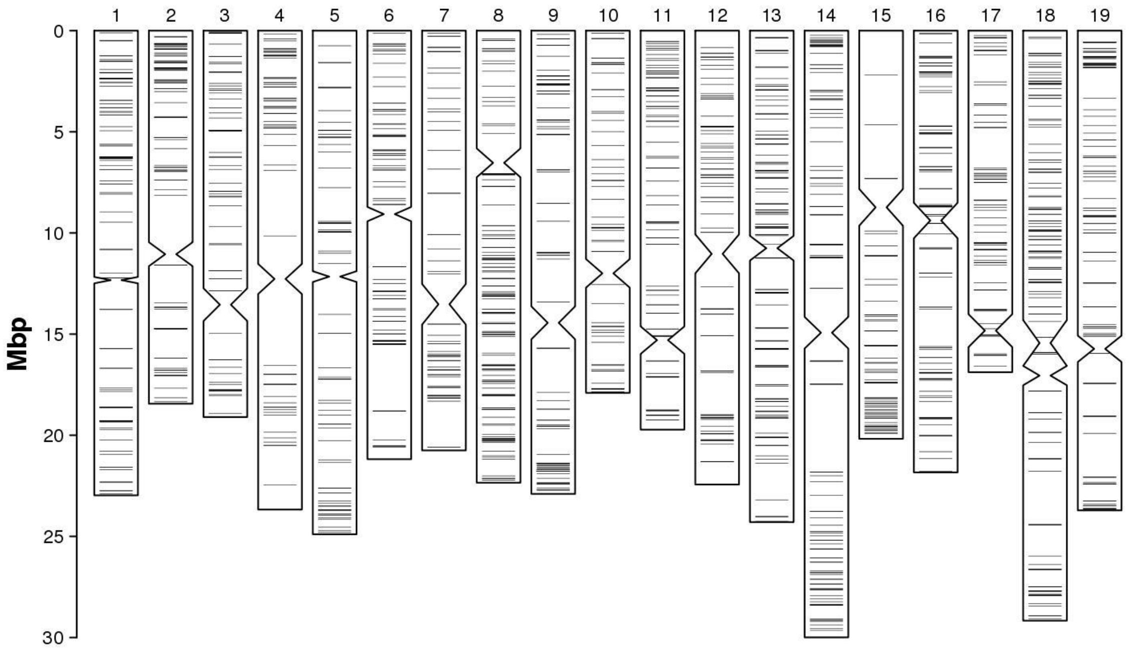

Fig. S23 Random distribution of unmatching SNPs (black ticks) across the genome for the 'Glavinuša' pedigree (Plavac Mali × Vugava Vis). Vertical ideograms represent chromosomes of the reference genome assembly of *V. vinifera* PN40024 12Xv0. Chromosome size is indicated on the y-axis in million base pairs (Mbp). Constrictions indicate the location of centromeric repeats.

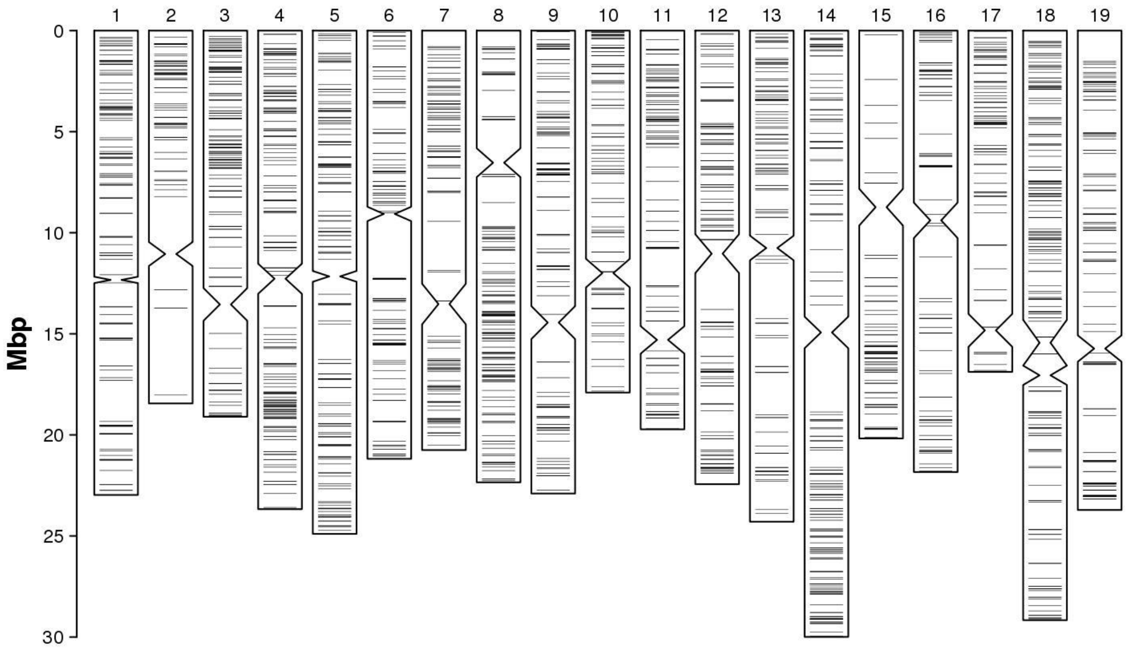

Fig. S24 Random distribution of unmatching SNPs (black ticks) across the genome for the 'Pošip Bijeli' pedigree (Zlatarica Blatska × Bratkovina Bijela). Vertical ideograms represent chromosomes of the reference genome assembly of *V. vinifera* PN40024 12Xv0. Chromosome size is indicated on the y-axis in million base pairs (Mbp). Constrictions indicate the location of centromeric repeats.

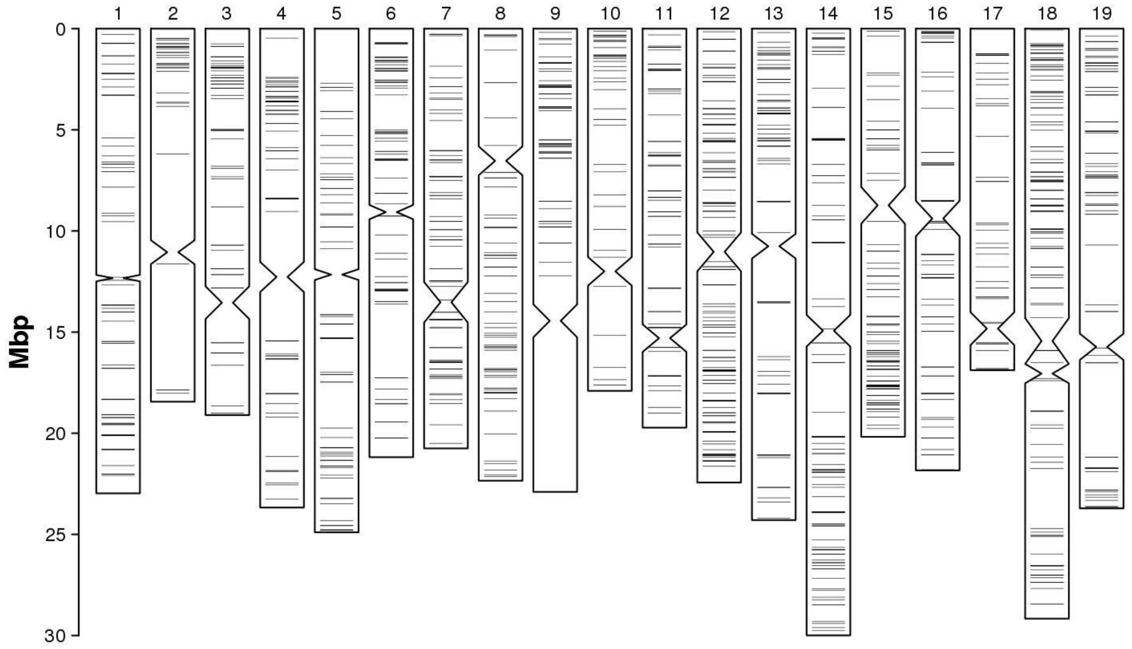

Fig. S25 Random distribution of unmatching SNPs (black ticks) across the genome for the 'Mejsko Bijelo' pedigree (Žumić × Duranija). Vertical ideograms represent chromosomes of the reference genome assembly of *V. vinifera* PN40024 12Xv0. Chromosome size is indicated on the y-axis in million base pairs (Mbp). Constrictions indicate the location of centromeric repeats.

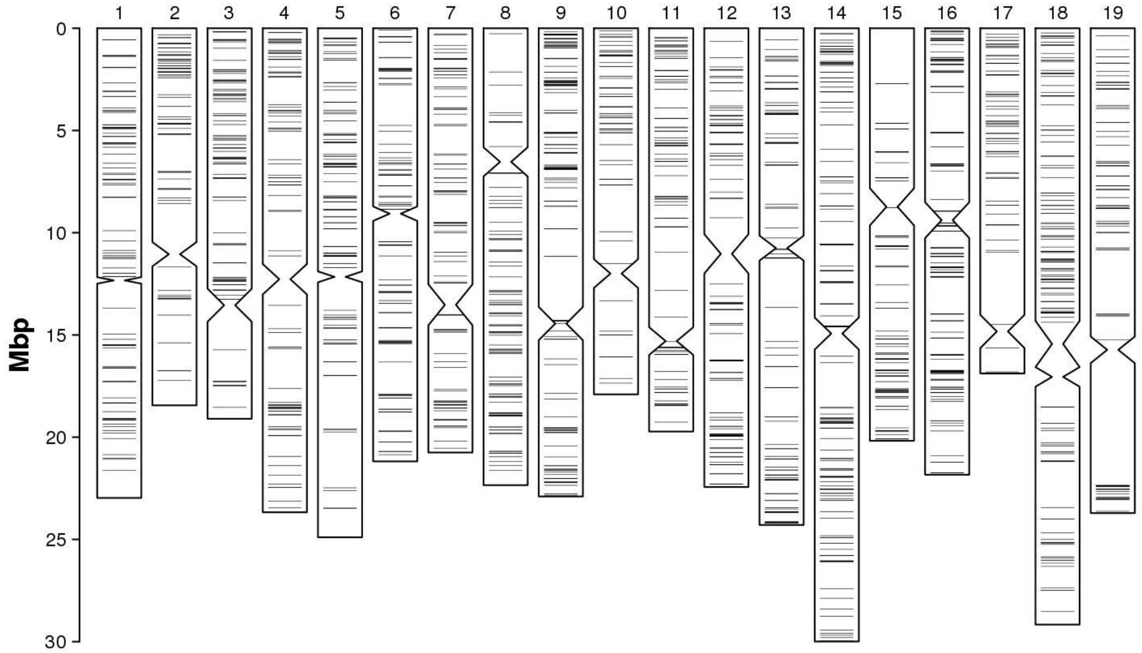

Fig. S26 Random distribution of unmatching SNPs (black ticks) across the genome for the 'Dolcin' pedigree (Malvasia Bianca Lunga × Glera). Vertical ideograms represent chromosomes of the reference genome assembly of *V. vinifera* PN40024 12Xv0. Chromosome size is indicated on the y-axis in million base pairs (Mbp). Constrictions indicate the location of centromeric repeats.

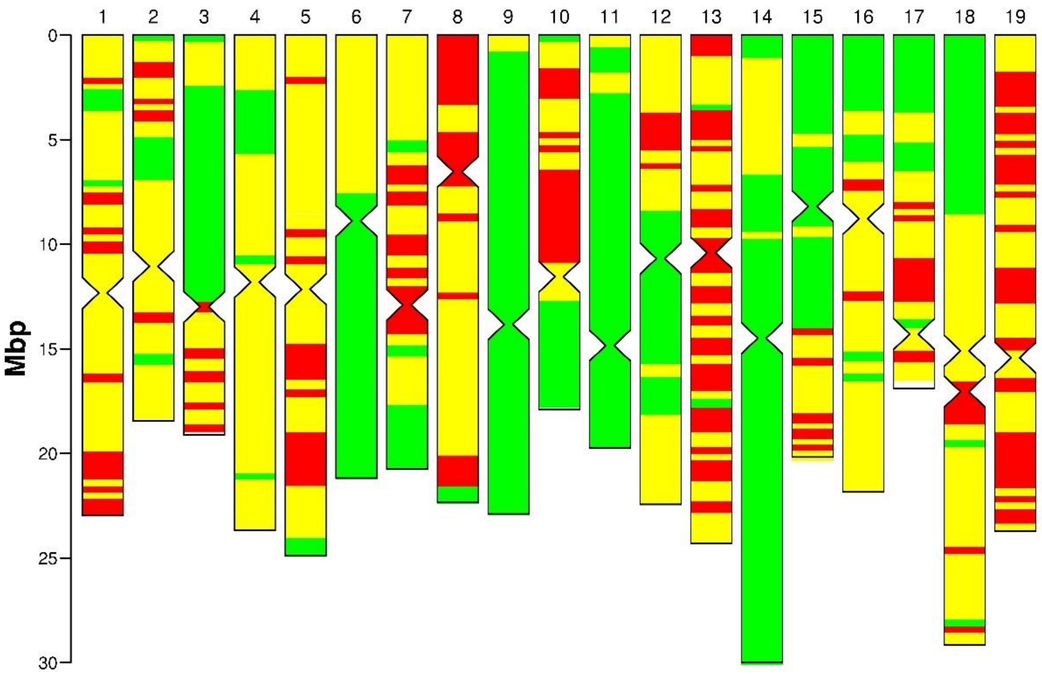

Fig. S27 Identity by descent (IBD) between ‘Plavec žuti’ and ‘Surina’. Vertical ideograms represent chromosomes of the reference genome assembly of *V. vinifera* PN40024 12Xv0. Chromosome size is indicated on the y-axis in million base pairs (Mbp). Constrictions indicate the location of centromeric repeats. Red indicates genomic windows with IBD=0 (no shared haplotype). Yellow indicates genomic windows with IBD=1 (one shared haplotype). Green indicates genomic windows with IBD=2 (two shared haplotypes). Each genomic window includes 200 Kb of non-repetitive DNA.

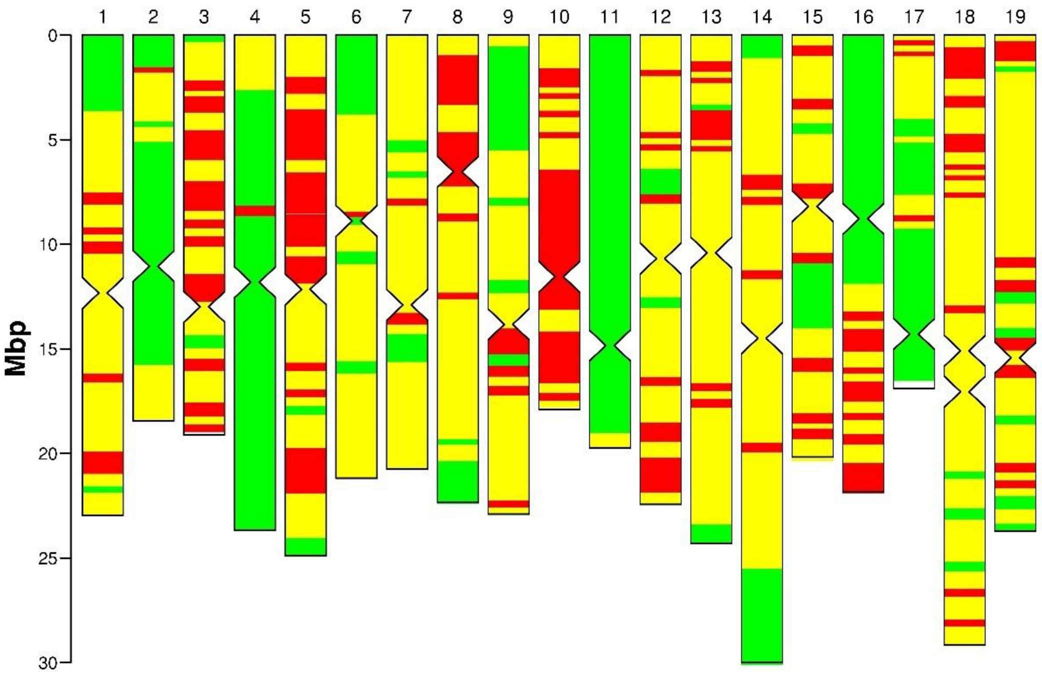

Fig. S28 Identity by descent (IBD) between 'Plavec žuti' and 'Ranfol'. Vertical ideograms represent chromosomes of the reference genome assembly of *V. vinifera* PN40024 12Xv0. Chromosome size is indicated on the y-axis in million base pairs (Mbp). Constrictions indicate the location of centromeric repeats. Red indicates genomic windows with IBD=0 (no shared haplotype). Yellow indicates genomic windows with IBD=1 (one shared haplotype). Green indicates genomic windows with IBD=2 (two shared haplotypes). Each genomic window includes 200 Kb of non-repetitive DNA.

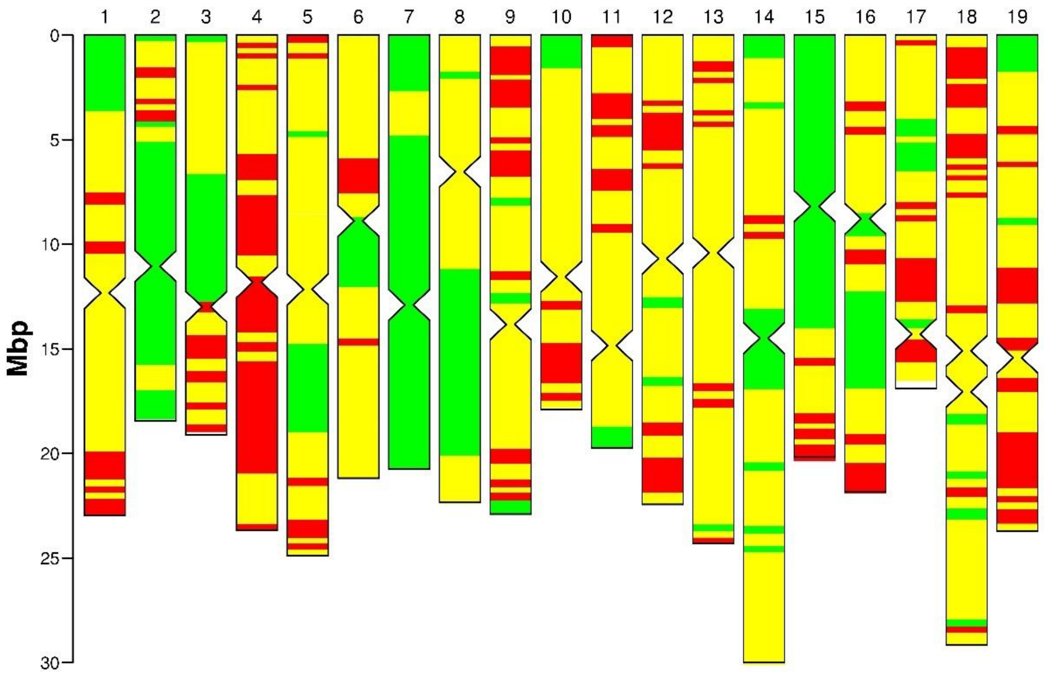

Fig. S29 Identity by descent (IBD) between 'Plavec žuti' and 'Svjetljak'. Vertical ideograms represent chromosomes of the reference genome assembly of *V. vinifera* PN40024 12Xv0. Chromosome size is indicated on the y-axis in million base pairs (Mbp). Constrictions indicate the location of centromeric repeats. Red indicates genomic windows with IBD=0 (no shared haplotype). Yellow indicates genomic windows with IBD=1 (one shared haplotype). Green indicates genomic windows with IBD=2 (two shared haplotypes). Each genomic window includes 200 Kb of non-repetitive DNA.

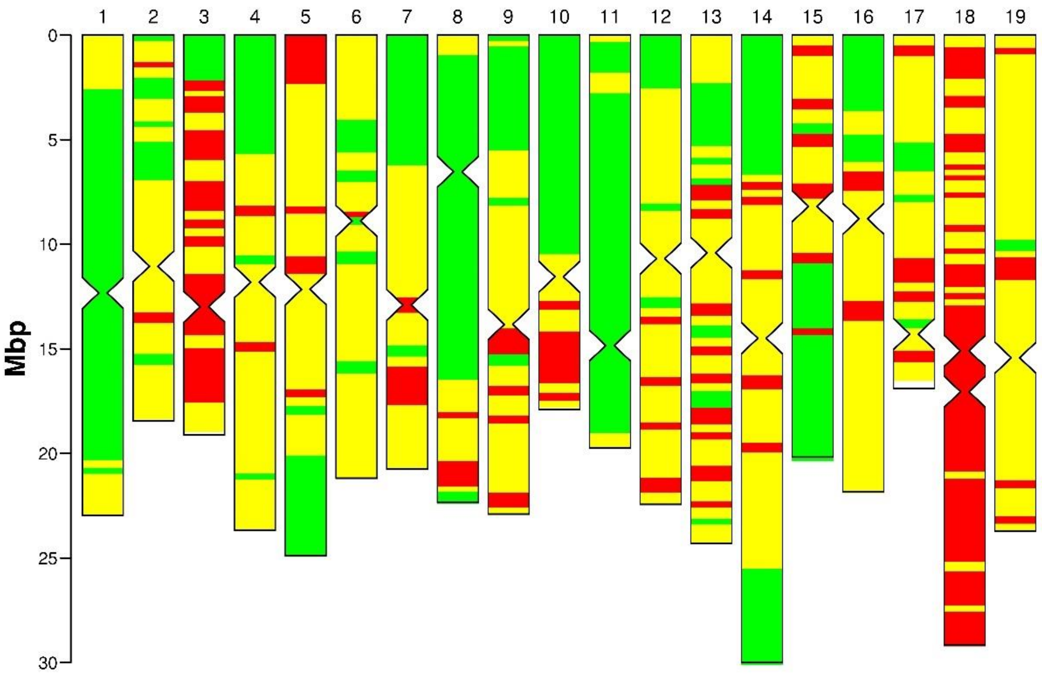

Fig. S30 Identity by descent (IBD) between ‘Ranfol’ and ‘Surina’. Vertical ideograms represent chromosomes of the reference genome assembly of *V. vinifera* PN40024 12Xv0. Chromosome size is indicated on the y-axis in million base pairs (Mbp). Constrictions indicate the location of centromeric repeats. Red indicates genomic windows with IBD=0 (no shared haplotype). Yellow indicates genomic windows with IBD=1 (one shared haplotype). Green indicates genomic windows with IBD=2 (two shared haplotypes). Each genomic window includes 200 Kb of non-repetitive DNA.

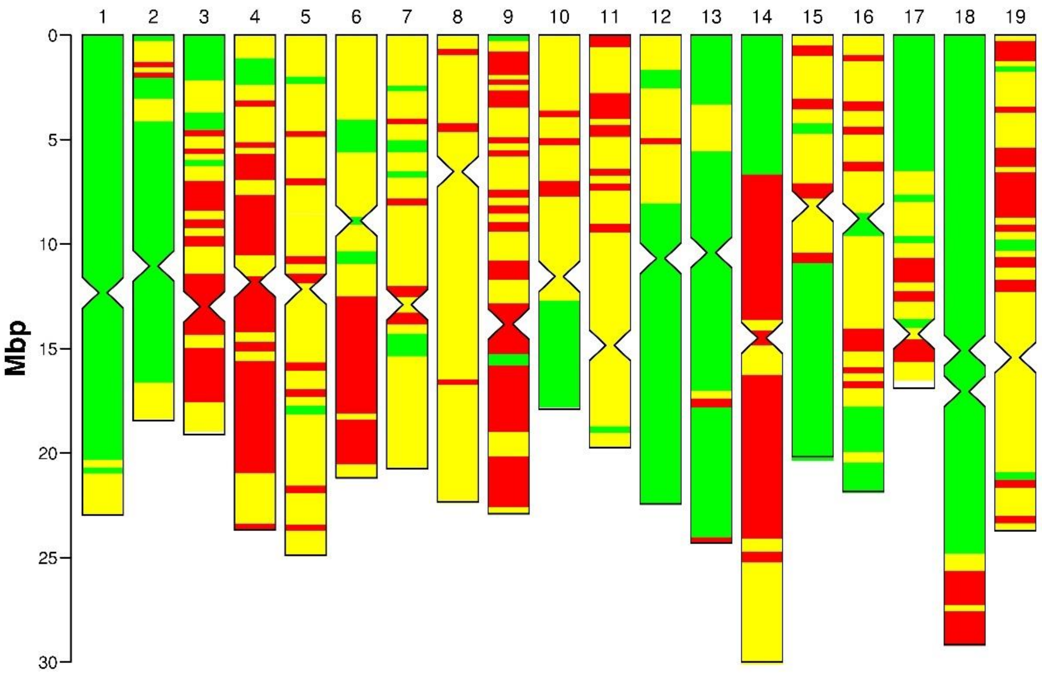

Fig. S31 Identity by descent (IBD) between 'Ranfol' and 'Svjetljak'. Vertical ideograms represent chromosomes of the reference genome assembly of *V. vinifera* PN40024 12Xv0. Chromosome size is indicated on the y-axis in million base pairs (Mbp). Constrictions indicate the location of centromeric repeats. Red indicates genomic windows with IBD=0 (no shared haplotype). Yellow indicates genomic windows with IBD=1 (one shared haplotype). Green indicates genomic windows with IBD=2 (two shared haplotypes). Each genomic window includes 200 Kb of non-repetitive DNA.

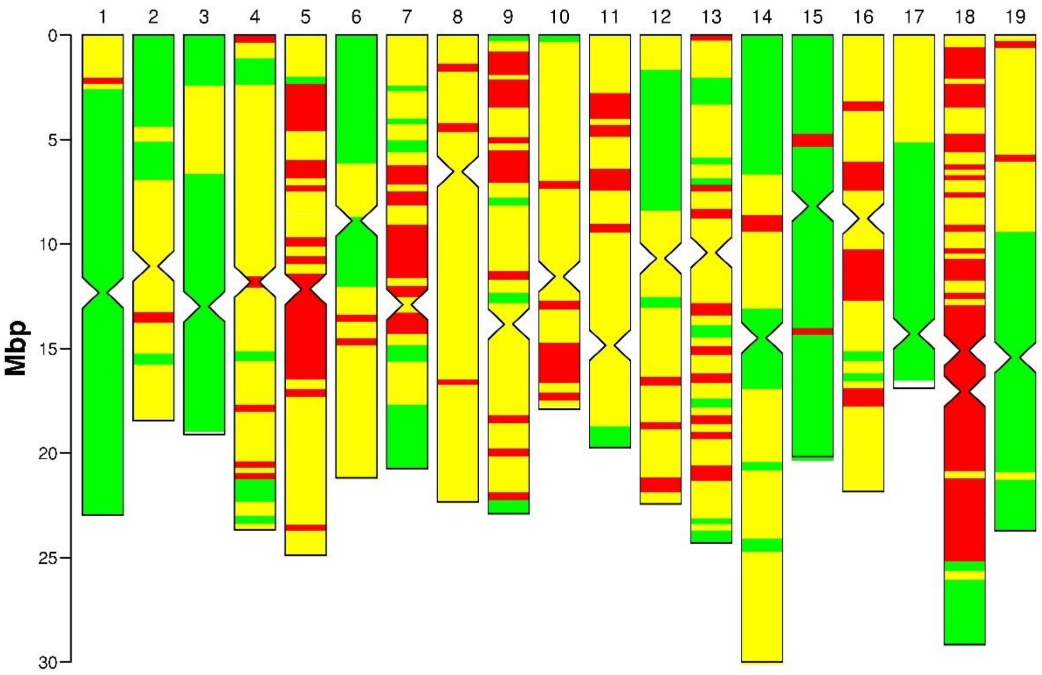

Fig. S32 Identity by descent (IBD) between ‘Svjetljak’ and ‘Surina’. Vertical ideograms represent chromosomes of the reference genome assembly of *V. vinifera* PN40024 12Xv0. Chromosome size is indicated on the y-axis in million base pairs (Mbp). Constrictions indicate the location of centromeric repeats. Red indicates genomic windows with IBD=0 (no shared haplotype). Yellow indicates genomic windows with IBD=1 (one shared haplotype). Green indicates genomic windows with IBD=2 (two shared haplotypes). Each genomic window includes 200 Kb of non-repetitive DNA.

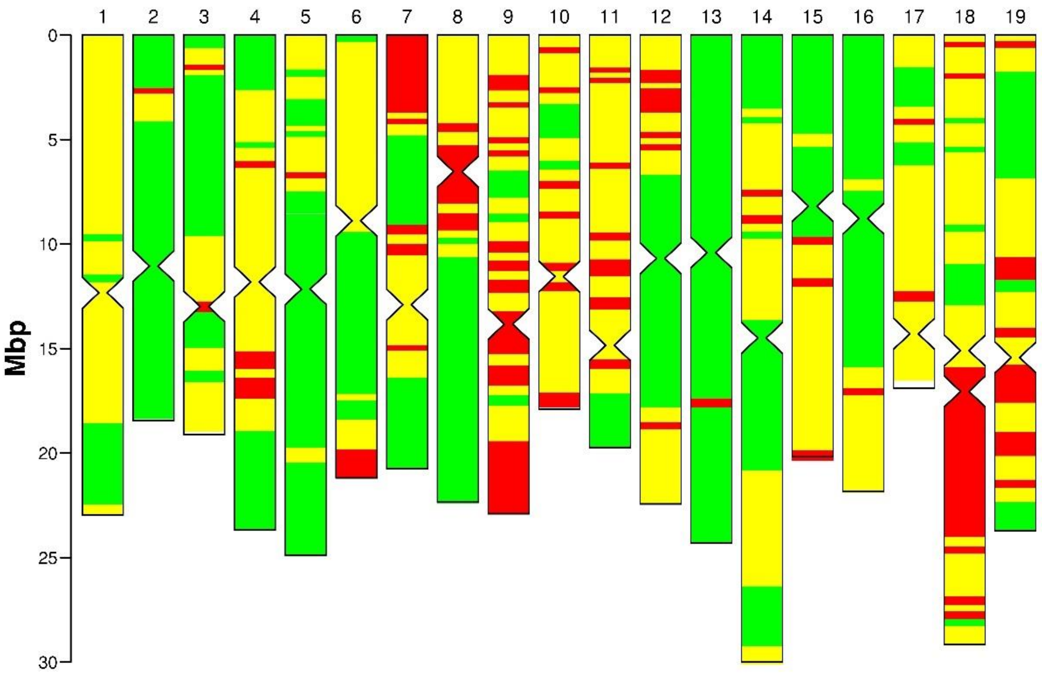

Fig. S33 Identity by descent (IBD) between 'Ljutun' and 'Ninčuša'. Vertical ideograms represent chromosomes of the reference genome assembly of *V. vinifera* PN40024 12Xv0. Chromosome size is indicated on the y-axis in million base pairs (Mbp). Constrictions indicate the location of centromeric repeats. Red indicates genomic windows with IBD=0 (no shared haplotype). Yellow indicates genomic windows with IBD=1 (one shared haplotype). Green indicates genomic windows with IBD=2 (two shared haplotypes). Each genomic window includes 200 Kb of non-repetitive DNA.

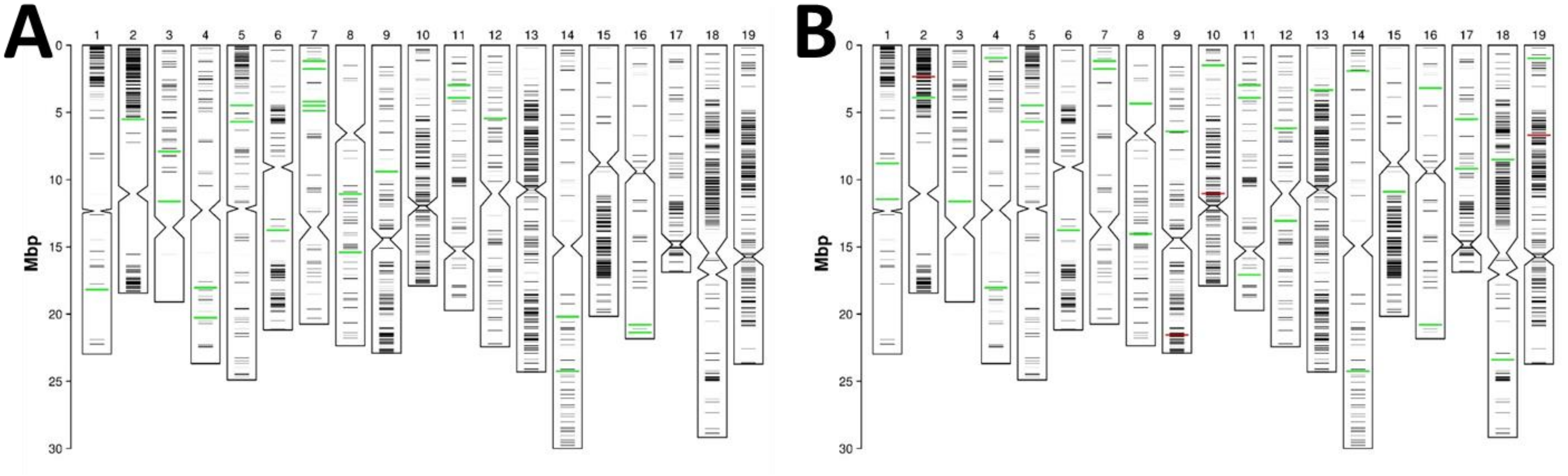

Fig. S34 Comparison between the chromosomal distribution of unmatching SNPs (black ticks) in the proposed trio 'Primitivo' x 'Dobričić' → 'Plavac Mali' as shown in Fig. 5 and the location of 25 microsatellite loci analysed by Maletić and coworkers (2004) shown in panel A or the location of 35 out of 36 microsatellite loci analysed by Žulj Mihaljević and coworkers (2020) shown in panel B. Vertical ideograms represent chromosomes of the reference genome assembly of *V. vinifera* PN40024 12Xv0. Chromosome size is indicated on the y-axis in million base pairs (Mbp). Constrictions indicate the location of centromeric repeats. Microsatellite loci were positioned by a BlastN search of the PCR primers referenced in Maletić et al (2004), Žulj Mihaljević et al (2020) and references therein against the *V. vinifera* PN40024 12Xv0 pseudomolecules. One of the 36 microsatellite loci analysed by Žulj Mihaljević and coworkers (2020), namely VCHR7b, could not be positioned on the chromosome pseudomolecules because it localized on an anchored scaffold that was assigned to Chr7_random. Two microsatellite loci used by Maletić et al (2004) are located close to one another on chromosome 4 and their ticks are overlapping at the graphical resolution of this plot (VrZAG83 Chr4:20.260 Mbp, VVMD26 Chr4:20.288 Mbp). In both panels, microsatellite loci locations are shown by coloured ticks. Green ticks indicate loci with microsatellites alleles in 'Plavac Mali' that are compatible with the reported pedigree. Red ticks indicate four loci with microsatellites alleles in 'Plavac Mali' that are not present in either presumed parent of the reported pedigree. Microsatellies revealing Mendelian inconsistencies in allele sizes in Žulj Mihaljević et al (2020) are: VVIB01 (Chr2:2.35 Mbp), VVIQ52 (Chr9:21.56 Mbp), VVIV37 (Chr10:1.10 Mbp) and VVIP31 (Chr19:6.70 Mbp). The microsatellite VCHR2b (Chr2:3.89 Mbp), which overlaps to a chromosomal segment that is not compatible with the proposed pedigree, was monomorphic in the three accessions (Žulj Mihaljević et al 2020). Microsatellite allele sizes are reported in Table 2 of the main text of the article Maletić et al (2004) and in Supplementary Table S6 of the article Žulj Mihaljević et al (2020).

References for Fig. S34

Žulj Mihaljević M, Maletić E, Preiner D, Zdunić G, Bubola M, Zyprian E, et al. Genetic diversity, population structure, and parentage analysis of Croatian grapevine germplasm. Genes. 2020. 11(7):1–35

Maletić E, Pejić I, Karoglan Kontić J, Piljac J, Dangl GS, Vokurka A, et al. Zinfandel, Dobričić, and Plavac mali: The genetic relationship among three cultivars of the Dalmatian Coast of Croatia. Am. J. Enol. Vitic. 2004. 55(2):174–180

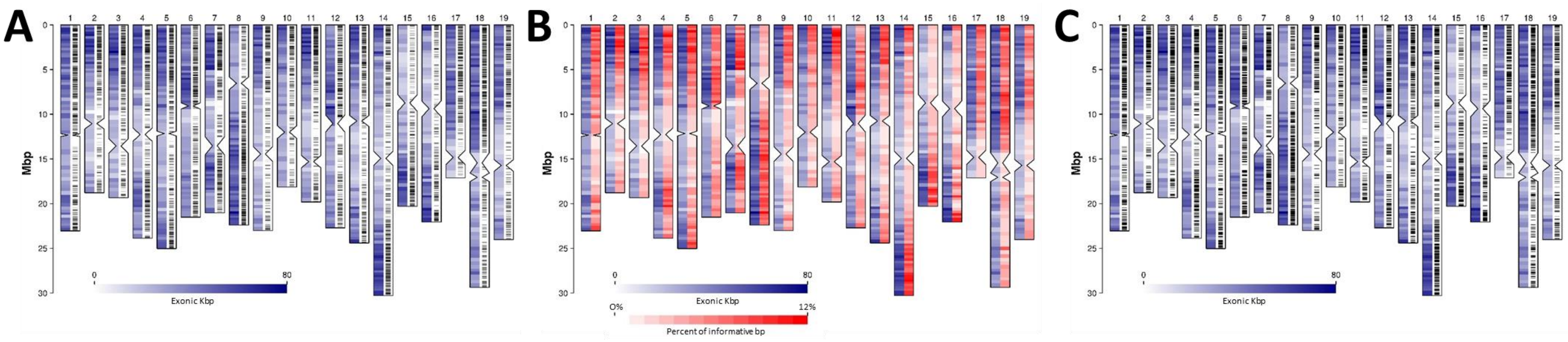

Fig. S35 Chromosomal patterns of gene density (A-C) compared to SPET primer distribution (A), percent of informative bases obtained from reduced-representation genome sequencing in non-overlapping windows of 200 Kb of non-repetitive DNA (B), and distribution of 306,965 SNPs with MAF >0.01 in a subset of 84 accessions used for GWAS (C). Vertical ideograms represent chromosomes. Constrictions indicate the location of centromeric repeats. Chromosomal plots of gene density are shown as white-to-blue heatmaps shown in the right-hand portion of each chromosome of all panels (**A-C**), reporting the number of exonic base pairs (Kbp) in non-overlapping windows of 200 Kb of non-repetitive DNA. (**A**) Black ticks in the right-hand portion of each chromosome indicate the position of 61,308 SPET primers. (**B**) The white-to-red heatmaps in the right-hand portion of each chromosome indicate the percentage of informative bases obtained with SPET sequencing. (**C**) Black ticks in the right-hand portion of each chromosome indicate SNP positions.

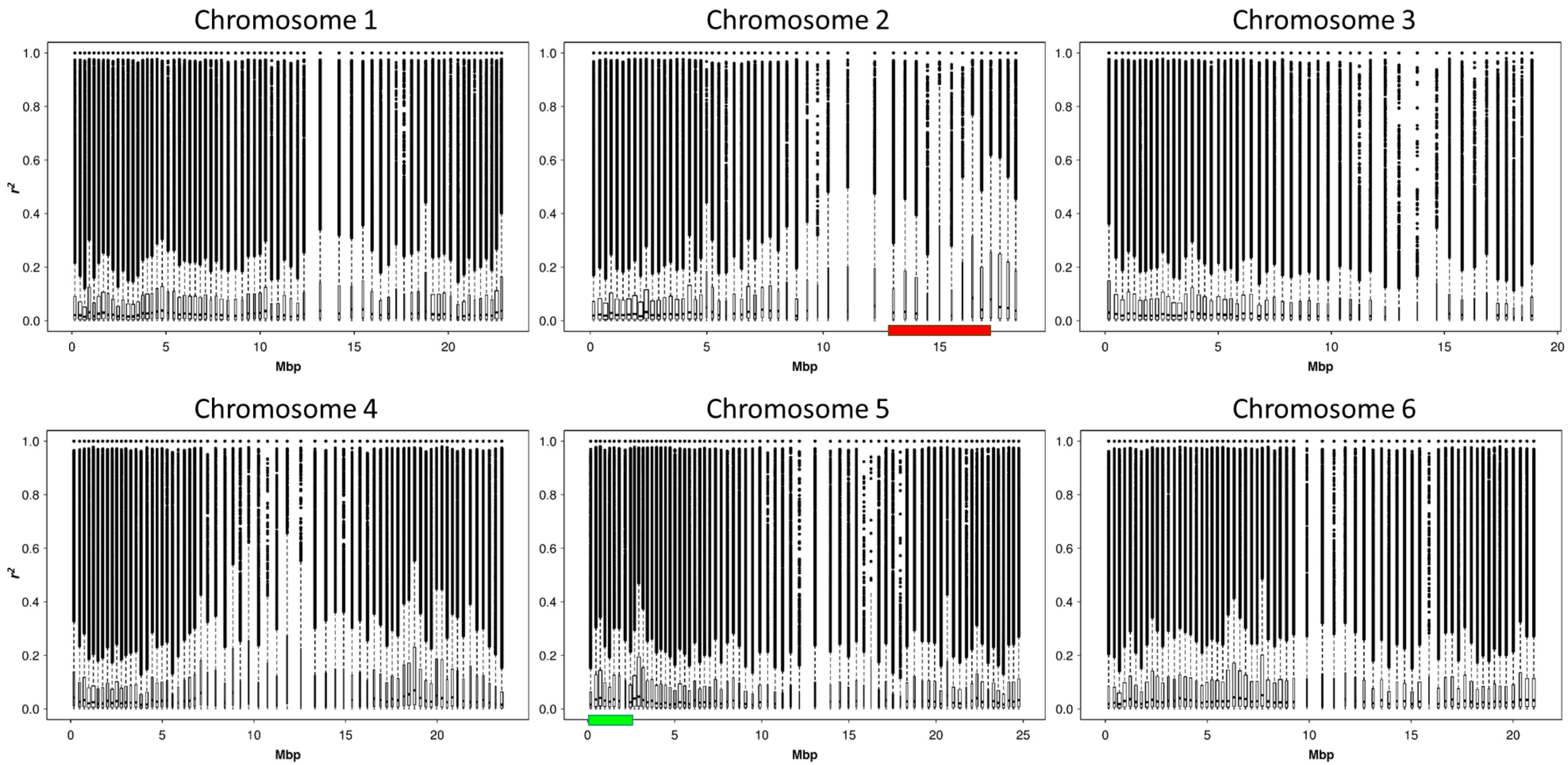

Fig. S36 Chromosomal patterns of correlation (*r^2*) between SNP genotypes in 84 accessions of the GWAS panel. r^2 was calculated between each SNP and all other downstream SNPs within a maximum distance of 200 Kbp . Local distributions of r^2 values are shown as box plots. Each box plot reports all r^2 values associated with the SNPs that reside in a window of 200 Kb of non-repetitive DNA. The width of each box is proportional to the number of SNPs in the genomic window. Each box is plotted in the middle position of the 200-Kb window. Black dots above the upper whisker represent outlier values of SNP genotypes in high linkage disequilibrium. Values of r^2 = 1 include SNP genotypes within stacks (i.e. reads obtained from DNA fragments generated by one SPET primer). The colored bars on the x-axis of chromosome 2 and 5 indicate the position of SNPs significantly associated with berry colour and leaf hairiness.

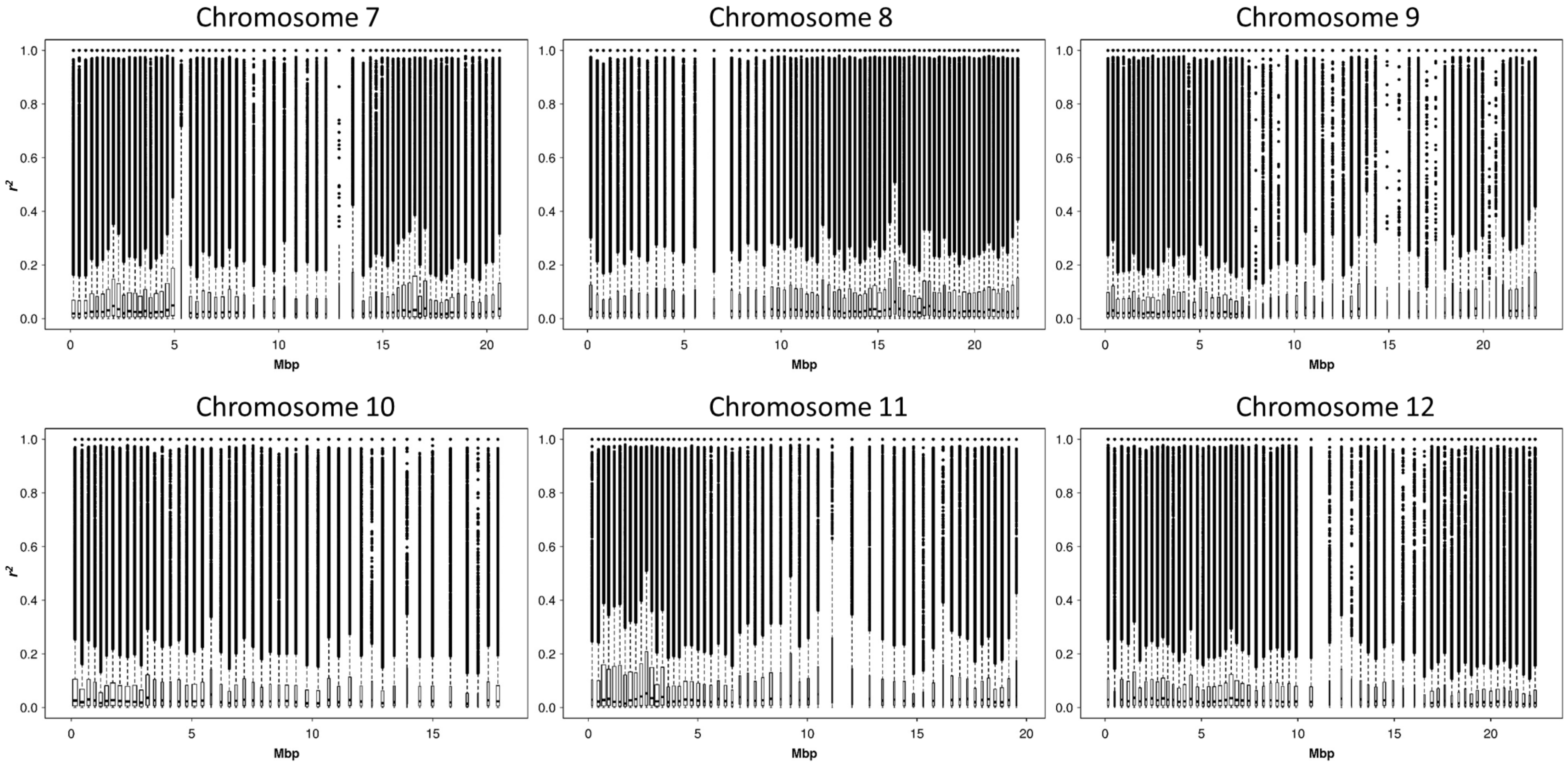

Fig. S36 (continued) Chromosomal patterns of correlation (r^2) between SNP genotypes in 84 accessions of the GWAS panel. r^2 was calculated between each SNP and all other downstream SNPs within a maximum distance of 200 Kbp. Local distributions of r^2 values are shown as box plots. Each box plot reports all r^2 values associated with the SNPs that reside in a window of 200 Kb of non-repetitive DNA. The width of each box is proportional to the number of SNPs in the genomic window. Each box is plotted in the middle position of the 200-Kb window. Black dots above the upper whisker represent outlier values of SNP genotypes in high linkage disequilibrium. Values of r^2 = 1 include SNP genotypes within stacks (i.e. reads obtained from DNA fragments generated by one SPET primer).

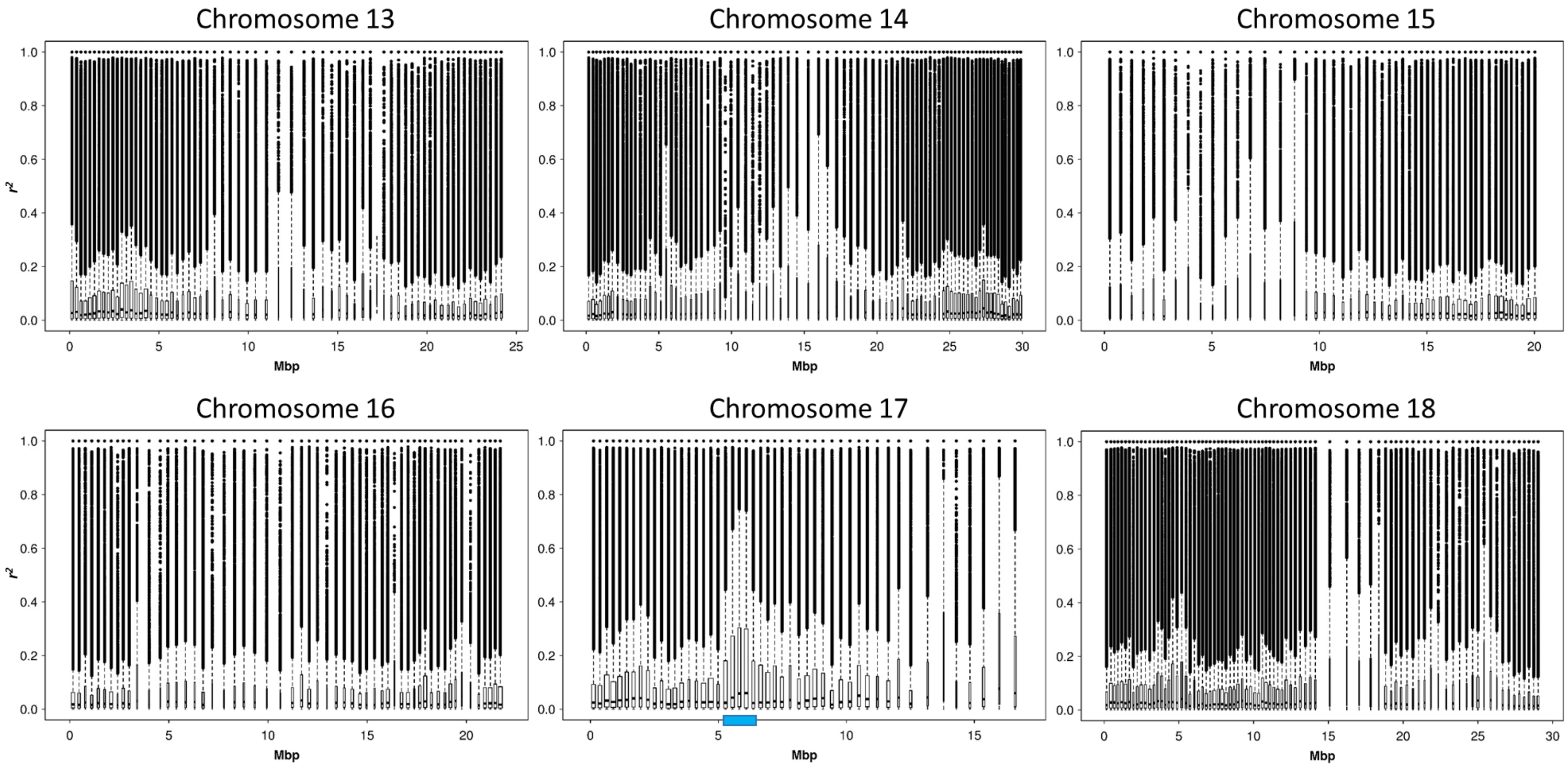

Fig. S36 (continued) Chromosomal patterns of correlation (r^2) between SNP genotypes in 84 accessions of the GWAS panel. r^2 was calculated between each SNP and all other downstream SNPs within a maximum distance of 200 Kbp. Local distributions of r^2 values are shown as box plots. Each box plot reports all r^2 values associated with the SNPs that reside in a window of 200 Kb of non-repetitive DNA. The width of each box is proportional to the number of SNPs in the genomic window. Each box is plotted in the middle position of the 200-Kb window. Black dots above the upper whisker represent outlier values of SNP genotypes in high linkage disequilibrium. Values of r^2 = 1 include SNP genotypes within stacks (i.e. reads obtained from DNA fragments generated by one SPET primer). The colored bar on the x-axis of chromosome 17 indicates the position of a known domestication locus.

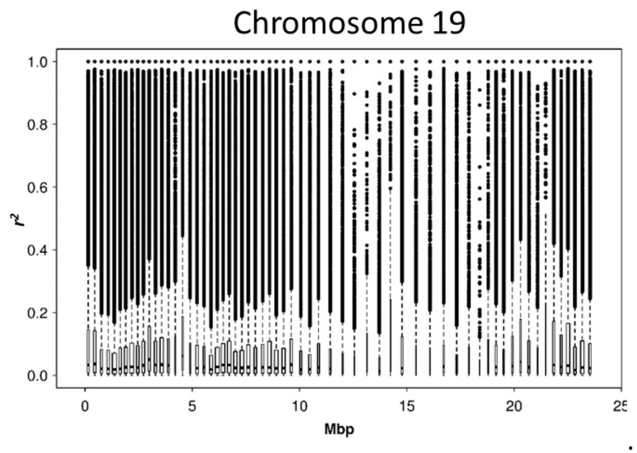

Fig. S36 (continued) Chromosomal patterns of correlation (r^2) between SNP genotypes in 84 accessions of the GWAS panel. r^2 was calculated between each SNP and all other downstream SNPs within a maximum distance of 200 Kbp. Local distributions of r^2 values are shown as box plots. Each box plot reports all r^2 values associated with the SNPs that reside in a window of 200 Kb of non-repetitive DNA. The width of each box is proportional to the number of SNPs in the genomic window. Each box is plotted in the middle position of the 200-Kb window. Black dots above the upper whisker represent outlier values of SNP genotypes in high linkage disequilibrium. Values of r^2 = 1 include SNP genotypes within stacks (i.e. reads obtained from DNA fragments generated by one SPET primer).

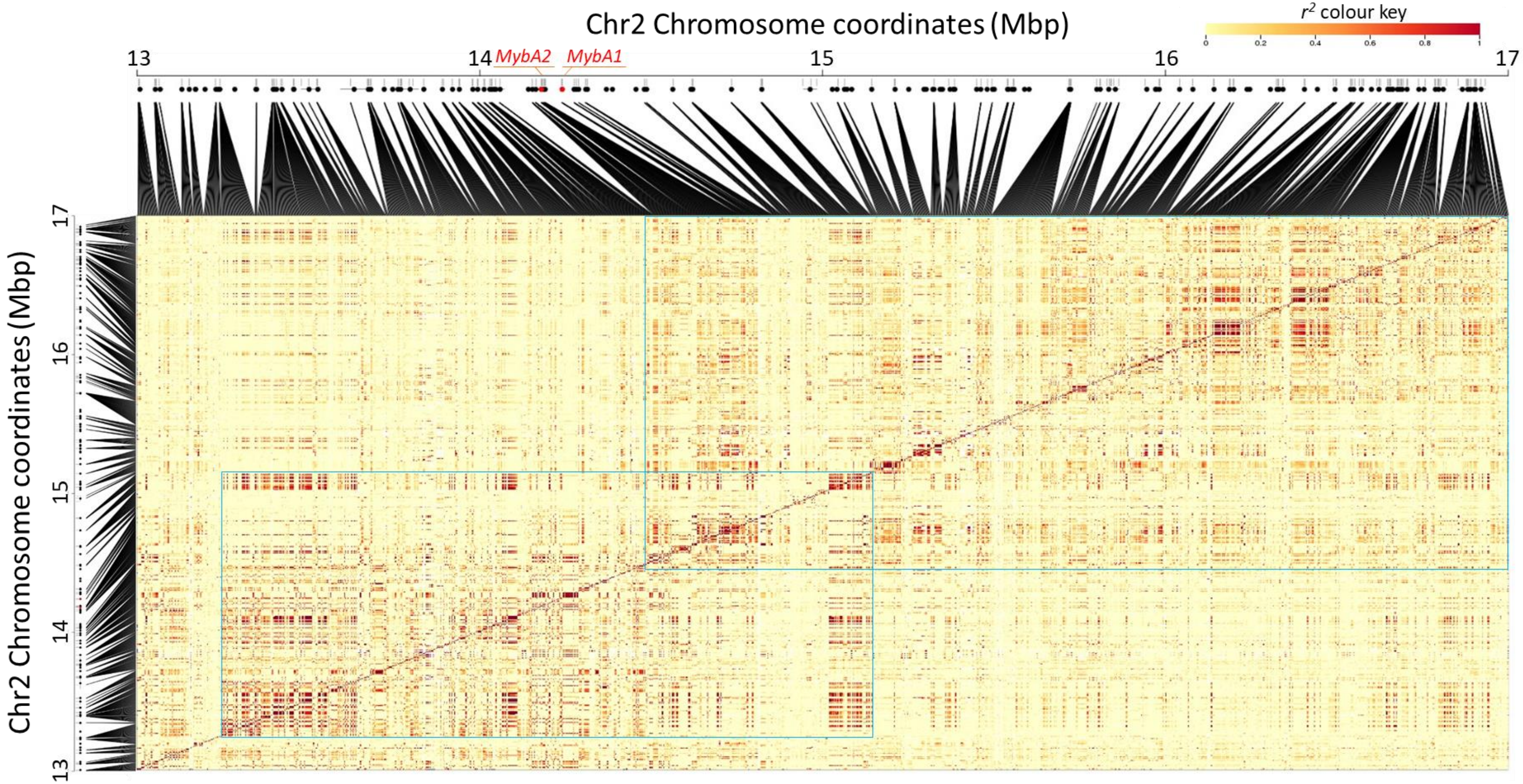

Fig. S37 Linkage disequilibrium, expressed as SNP genotype correlation (r^2) between pairs of SNPs, in a 4-Mbp region in the lower arm of Chromosome 2. The yellow-to-red heatmap indicates r^2 values. The cyan rectangles indicate two regions of long-range correlation over Mbp-scale distances forming large haplotypic blocks that include SNPs tagging *MybA* genes (Chr2:14.1-14.3 Mbp). The *MybA* copies responsible for white/red colour variation (*MybA1* and *MybA2*) are indicated in red. Black dots indicate the physical position of predicted genes. Dots are centered on the coordinate of the middle position of the gene models. If the length of the gene model was larger than the circumference of the dot, the actual gene size is indicated by a horizontal bar protruding from the dot. The black connectors connect the position of the SNPs in the r^2 matrix with the position of the gene model in which they reside.

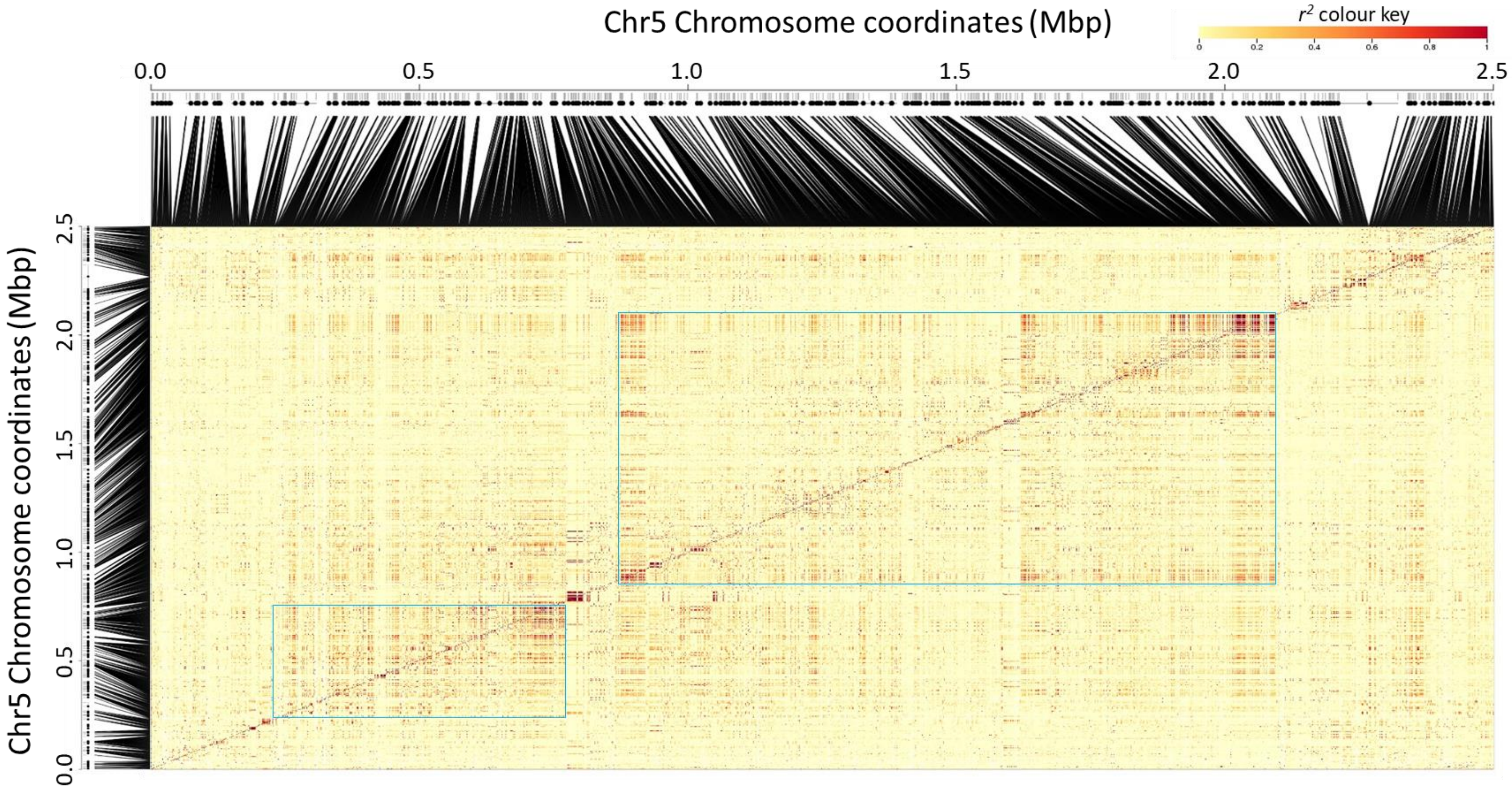

Fig. S38 Linkage disequilibrium, expressed as SNP genotype correlation (r^2) between pairs of SNPs, in a 2.5-Mbp region in the upper end of Chromosome 5, which showed association between SNP genotypes and leaf hairiness. The yellow-to-red heatmap indicates r^2 values. The cyan rectangles indicate two regions of long-range correlations over Mbp-scale distances forming large haplotypic blocks. Black dots indicate the physical position of predicted genes. Dots are centered on the coordinate of the middle position of the gene models. If the length of the gene model was larger than the circumference of the dot, the actual gene size is indicated by a horizontal bar protruding from the dot. The black connectors connect the position of the SNPs in the r^2 matrix with the position of the gene model in which they reside.
